# Tuning the antimicrobial activity of microbial glycolipid biosurfactants through chemical modification

**DOI:** 10.3389/fbioe.2024.1347185

**Published:** 2024-02-14

**Authors:** Melike Pala, Martijn G. Castelein, Camille Dewaele, Sophie L. K. W. Roelants, Wim K. Soetaert, Christian V. Stevens

**Affiliations:** ^1^ SynBioC, Department of Green Chemistry and Technology, Faculty of Bioscience Engineering, Ghent University, Ghent, Belgium; ^2^ Department of Biotechnology, Centre for Industrial Biotechnology and Biocatalysis (InBio.be), Faculty of Bioscience Engineering, Ghent University, Ghent, Belgium; ^3^ Bio Base Europe Pilot Plant (BBEPP), Ghent, Belgium

**Keywords:** microbial biosurfactants, sophorolipids, sophorosides, chemical modification, antimicrobial activity

## Abstract

Sophorolipids, glycolipid biosurfactants derived from microorganisms such as *Starmerella bombicola*, possess distinctive surface-active and bioactive properties, holding potential applications in cosmetics, pharmaceuticals and bioremediation. However, the limited structural variability in wild-type sophorolipids restricts their properties and applications. To address this, metabolic engineering efforts have allowed to create a portfolio of molecules. In this study, we went one step further by chemically modifying microbially produced sophorosides, produced by an engineered *S. bombicola.* Twenty-four new sophoroside derivatives were synthesized, including sophoroside amines with varying alkyl chain lengths (ethyl to octadecyl) on the nitrogen atom and their corresponding quaternary ammonium salts. Additionally, six different microbially produced glycolipid biosurfactants were hydrogenated to achieve fully saturated lipid tails. These derivatives, along with microbially produced glycolipids and three benchmark biosurfactants (di-rhamnolipids, alkyl polyglucosides, cocamidopropyl betaine), were assessed for antimicrobial activity against bacteria (*Bacillus subtilis, Staphylococcus aureus, Listeria monocytogenes, Escherichia coli, Pseudomonas aeruginosa*) and yeast (*Candida albicans*). Results indicated that microbially produced glycolipids, such as bola sophorosides, acidic sophorolipids and acidic glucolipids exhibit selective antimicrobial activity against the test organisms. Conversely, lactonic sophorolipids, sophoroside amines and quaternary ammonium salts display a broad antimicrobial activity. *N-octyl, N-dodecyl* and *N-octadecyl* derivatives exhibit the lowest minimal inhibitory concentrations, ranging from 0.014 to 20.0 mg mL^−1^. This study demonstrates the potential synergy of thoughtful biotechnology and targeted chemistry to precisely tailor glycolipid biosurfactants to meet specific requirements across applications.

## 1 Introduction

**FIGURE 1:**
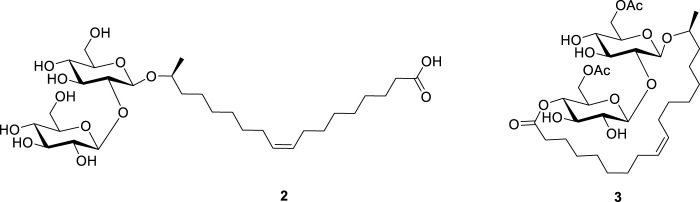
The wild-type mixture of sophorolipids (1) consisting of nonacetylated sophorolipid acid (2) and diacetylated sophorolipid lactone (3).

Sophorolipids (SLs) are a class of microbial glycolipid biosurfactants produced by different non-pathogenic yeasts such as *Starmerella bombicola* using renewable carbon sources that are derived from first generation biomass (Jezierska et al., 2018; Rau et al., 2001; Van Bogaert et al., 2011) as well as from side- or waste streams (Takahashi et al., 2011; Kaur et al., 2019; Wang et al., 2019)*.* The major glycolipid species produced by the wild type *S. bombicola* strain (i.e., the wild-type mixture **1**) consist of open-chain acidic **2** and lactonic SLs **3** with various degrees of acetylation on the sophorose head (Figure 1). The combination of the hydrophilic sophorose head and the hydrophobic lipid tail makes these glycolipids surface-active compounds. Additionally, they show beneficial bioactive properties such as antimicrobial (Shah et al., 2007), anti-cancer (Chen et al., 2006; Nawale et al., 2017), dermatological, immunoregulatory (Shah et al., 2005) and antiviral activities. With these favorable properties SLs are currently one of the most represented microbial biosurfactants in the market world-wide (Hayes and Smith, 2019).

Nevertheless, their application is currently mostly limited to ecological cleaning and personal care products (Develter and Lauryssen, 2010; Dierickx et al., 2022). Expanding their structural variety is needed to pave the way towards further uses in pharmaceuticals and personal care products, etc. To this end, metabolic engineering efforts have already led to the development of a portfolio of new-to-nature glycolipids, although it still has a rather narrow range of structural variation and focus was placed on uniform production (Van Renterghem et al., 2018a; Castelein et al., 2023). The current production efficiencies of the new-to-nature glycolipids are compound-specific, still quite variable and generally lower when compared to wild type SLs. The combination of genetic engineering and chemical modification of both wild-type and new-to-nature glycolipids allows the generation of a wider range of novel structures. Indeed, SLs and new-to-nature glycolipids, especially the ones that are already produced with high productivities, like sophorosides (SSs), are ideal candidates as building blocks for chemical modification because of their rather complex structure. Moreover, the biological activity of the biosurfactants could be altered by generating such a new set of derivatives. Literature shows that modified SLs have high potential as antimicrobial agents (Azim et al., 2006; Totsingan et al., 2021), in particular the SL derivatives with a cationic charge (Tang et al., 2021; Delbeke et al., 2018; Delbeke et al., 2015a; Delbeke et al., 2019; Speight et al., 2022). These studies revealed that the balance between the cationic charge and the hydrophobic group plays a key role in the lysis of bacterial cells. The cationic charge is necessary to create an electrostatic interaction between the surfactant and the cell surface while the hydrophobic group facilitates the insertion into the lipid layer of the bacterial cell membrane. For example, in a recent study of Delbeke et al., the quaternary ammonium SLs with a long alkyl chain (i.e., dodecyl, pentadecyl and octadecyl) attached on the nitrogen atom displayed better antimicrobial activities compared to lactonic SLs against Gram-negative (*E. coli* LMG 8063, *Klebsiella pneumoniae* LMG 2095 and *Pseudomonas aeruginosa* PAO1) and Gram-positive (*Staphylococcus aureus* ATCC 6538 and *S. aureus* Mu50) bacteria (Delbeke et al., 2019). Similarly, arginine conjugated SL derivatives broaden the antimicrobial spectrum by showing a high potency against Gram-positive (*Bacillus subtilis, Bacillus cereus, S. aureus*) and Gram-negative (*Escherichia coli*) bacteria and two fungal strains (*Moesziomysces sp*. and *Candida albicans*), with improved antimicrobial activity compared to acidic SLs (Tang et al., 2021). On the other hand, the hydrophobic leucine conjugated SL derivatives did not inhibit the growth of any bacterium or fungus, indicating that electrostatic interaction with the cell membrane also plays a major role in the inhibition of pathogens.

**FIGURE 2:**
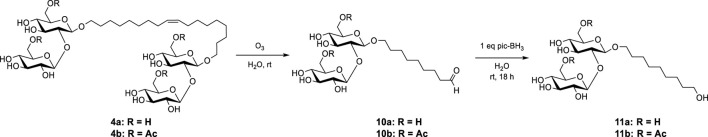
Ozonolysis of (tetra acetylated) bola sophoroside 4 towards (di acetylated) C9 sophoroside aldehyde 10 and its subsequent reduction to (di acetylated) sophoroside alcohol 11.

In this study, we aimed to diversify the class of SSs by synthesizing a new set of SS amines (SS amines) with increasing alkyl chain length on the nitrogen atom and their corresponding sophoroside quaternary salts (SS quats), starting from novel microbial biosurfactants, i.e., bola SSs (Figure 2). The incorporation of an amine functionality into SSs renders them pH-responsive, allowing them to change their charge in solution depending on the acidity of the environment. The pH responsiveness of amine-functionalized SSs makes them particularly interesting for use in drug delivery, as they can be designed to release their payload in response to changes in the pH of the surrounding tissue. Additionally, the ability to change their charge as a function of the pH could tune the effectiveness of penetrating cell membranes. On the other hand, the quaternization of SSs is useful in applications where a permanent positive charge is needed, such as in the creation of antimicrobial coatings or in the stabilization of emulsions in the food industry (Bai et al., 2018). By adding a positively charged group to the molecule, the resulting SS becomes attracted to negatively charged surfaces or particles, allowing the compound to form strong ionic bonds and improve the overall stability of the system.

Furthermore, a second set of chemically modified glycolipids was obtained via the hydrogenation of six different microbially produced glycolipids. Such hydrogenation allows the synthesis of fully saturated hydrophobic tails, which in turn allows to study the effect of increased hydrophobicity.

Subsequently, the antimicrobial activities of the new sets of glycolipid derivatives were investigated, next to the microbially produced glycolipids for comparison, against Gram-positive bacteria (*B. subtilis*, *S*. *aureus*, *Listeria monocytogenes*), Gram-negative bacteria (*E. coli*, *P*. *aeruginosa*) and one yeast strain (*C*. *albicans*).

This study can serve as a foundation for the development of tailored glycolipids or derivatives possessing targeted physicochemical and biological characteristics.

## 2 Materials and methods

The list of tested microbially produced glycoplipids, chemically derived SSs, benchmark surfactants and their accompanying specifications can be found in Table 1.

**TABLE 1:** List of compounds tested during the antimicrobial assay. The dry matter of the compound was taken into account to prepare the stock solution of 200 g L^−1^. *The dry matter of chemical derivatives was considered to be 100%.

	Code	Glycolipid	Fatty acid/fatty alcohol^b^	Acetylation	MW (g/mol)	Solvent
Microbially produced glycolipids						
	**Wild type sophorolipids**					
**1**	WT	Wild type mix SL^a^	C18:1	0–2	654 (av.)	DMSO
**2**	NonAc acidic SL (C18:1)	Non-acetylated acidic SL	C18:1	0	622	DMSO
**3**	Ac lactonic SL (C18:1)	Acetylated lactonic SL	C18:1	1–2	646	DMSO
	**New-to-nature sophorosides**					
**4a**	NonAc bola SS (C18:1)	Non-acetylated bola SS	C18:1	0	932	Milli-Q
**4b**	Ac bola SS (C18:1)	Acetylated bola SS	C18:1	1–4	1,002	Milli-Q
**5a**	NonAc acidic GL (C18:1)	Non-acetylated GL	C18:1	0	460	DMSO
**5b**	Ac acidic GL (C18:1)	Mix acetylated GL	C18:1	0–1	470	Milli-Q
Chemically derived glycolipids						
	**Hydrogenated glycolipids**					
**6**	NonAc acidic SL (C18:0)	Non-acetylated acidic SL	C18:0	0	934	DMSO
**7**	Ac lacton SL (C18:0)	Acetylated lactonic SL	C18:0	1–2	648	DMSO
**8a**	Nonac bola SS (C18:0)	Non-acetylated bola SS	C18:0	0	934	DMSO
**8b**	Ac bola SS (C18:0)	Acetylated bola SS	C18:0	1–4	1,004	DMSO
**9a**	NonAc acidic GL (C18:0)	Non-acetylated GL	C18:0	0	462	DMSO
**9b**	Ac acidic GL (C18:0)	Mix acetylated GL	C18:0	0–2	472	DMSO
	**Short chain sophorosides**					
**10a**	NonAc aldehyde SS (C9:0)	Non-acetylated aldehyde	C9:0	0	482	Milli-Q
**10b**	Ac aldehyde SS (C9:0)	Acetylated aldehyde	C9:0	2	566	Milli-Q
**11a**	NonAc alcohol SS (C9:0)	Non-acetylated alcohol	C9:0	0	484	DMSO
**11b**	Ac alcohol SS (C9:0)	Acetylated alcohol	C9:0	2	568	Milli-Q
	**Sophoroside Amines**					
**12a**	NonAc SS amine (C2)	Non-acetylated Amine	C9:0-C2:0	0	525	Milli-Q
**13a**	NonAc SS amine (C4)	Non-acetylated Amine	C9:0-C4:0	0	553	Milli-Q
**14a**	NonAc SS amine (C6)	Non-acetylated Amine	C9:0-C6:0	0	581	Milli-Q
**15a**	NonAc SS amine (C8)	Non-acetylated Amine	C9:0-C8:0	0	609	Milli-Q
**16a**	NonAc SS amine (C12)	Non-acetylated Amine	C9:0-C12:0	0	665	Milli-Q
**17a**	NonAc SS amine (C18)	Non-acetylated Amine	C9:0-C18:0	0	749	Milli-Q
**12b**	Ac SS amine (C2)	Non-acetylated Amine	C9:0-C2:0	0	609	Milli-Q
13b	Ac SS amine (C4)	Non-acetylated Amine	C9:0-C4:0	0	637	Milli-Q
14b	Ac SS amine (C6)	Non-acetylated Amine	C9:0-C6:0	0	665	Milli-Q
15b	Ac SS amine (C8)	Non-acetylated Amine	C9:0-C8:0	0	693	Milli-Q
16b	Ac SS amine (C12)	Non-acetylated Amine	C9:0-C12:0	0	749	Milli-Q
17b	Ac SS amine (C18)	Non-acetylated Amine	C9:0-C18:0	0	833	Milli-Q
	**Sophoroside quaternary ammoniums salts**					
**18a**	NonAc SS quat (C2)	Non-acetylated quat	C9:0-C2:0	0	666	Milli-Q
**19a**	NonAc SS quat (C4)	Non-acetylated quat	C9:0-C4:0	0	694	Milli-Q
**20a**	NonAc SS quat (C6)	Non-acetylated quat	C9:0-C6:0	0	722	Milli-Q
**21a**	NonAc SS quat (C8)	Non-acetylated quat	C9:0-C8:0	0	750	Milli-Q
**22a**	NonAc SS quat (C12)	Non-acetylated quat	C9:0-C12:0	0	806	Milli-Q
**23a**	NonAc SS quat (C18)	Non-acetylated quat	C9:0-C18:0	0	890	Milli-Q
**18b**	Ac SS quat (C2)	Non-acetylated quat	C9:0-C2:0	0	750	Milli-Q
**19b**	Ac SS quat (C4)	Non-acetylated quat	C9:0-C4:0	0	778	Milli-Q
**20b**	Ac SS quat (C6)	Non-acetylated quat	C9:0-C6:0	0	806	Milli-Q
**21b**	Ac SS quat (C8)	Non-acetylated quat	C9:0-C8:0	0	834	Milli-Q
**22b**	Ac SS quat (C12)	Non-acetylated quat	C9:0-C12:0	0	890	Milli-Q
**23b**	Ac SS quat (C18)	Non-acetylated quat	C9:0-C18:0	0	974	Milli-Q
**BM1**		Di-Rhamnolipid			651	Milli-Q
**BM2**		Alkyl polyglucosides			320	Milli-Q
**BM3**		Cocamidopropyl betaine			342	Milli-Q

^a^ Consisting of 60% lactonic sophorolipids and 40% acidic sophorolipids.

^b^ The first carbon value refers to the chain length between the sophorose head group and the nitrogen while the second carbon value refers to the chain length of the alkyl chain on the nitrogen atom. The number following the colon (:) shows the saturation degree of the compound (e.g., C9:0—C2:0).

SS, sophoroside; SL, sophorolipid; GL, glucolipid; Quats, quaternary ammonium salt; DMSO, dimethyl sulfoxide.

### 2.1 Microbially produced glycolipids (1–5), benchmark surfactants and chemicals

Synthesis of the different (acetylated) C18:1 sophorolipids (SL)/sophorosides (SS) has been previously reported through aerobic fermentation with different strains of the yeast *S. bombi*cola as discussed in (Castelein et al., 2021; Baccile et al., 2016; Van Renterghem et al., 2018b). Synthesis of the bola SS precursor has been extensively described in (Van Renterghem et al., 2018b). The wild type mixture of SL **1** (acidic and lactonic SL), non-acetylated acidic SL **2**, acetylated lactonic SL **3**, non-acetylated bola SS **4a**, acetylated bola SS **4b**, non-acetylated glucolipid (GL) **5a** and mix acetylated GL **5b** were obtained from Bio Base Europe Pilot Plant (BBEPP, Ghent) and used without further purification (purity above 97.6%). The non-acetylated acidic SL **2**, non-acetylated **4a** and acetylated bola SS **4b** and non-acetylated glucolipids **5a** were provided in solid powder form with a dry matter content of ca. 100%, while the wild type mixture **1** (dry matter of 55.0%), acetylated lactonic SL **3** (dry matter of 64.8%) and mix acetylated GL **5b** (dry matter of 55.9%) were provided as a viscous aqueous solution. The dry matter (DM) percentage was determined using an Infrared Balance (105 °C) and was taken into account to obtain the correct biosurfactant concentrations for the stock solutions. The purity of the glycolipids was determined using ultra high-performance liquid chromatography (UPLC) coupled to an evaporative light scattering detector (ELSD) whereby residual glucose, glycerol, free fatty acid and oil were taken into account. These UPLC-ELSD analyses were performed on a Acquity H-Class UPLC (Waters) and Acquity ELSD Detector (Waters) with an Acquity UPLC CSH C18 column (130 Å, 1.7 μm, 2.1 mm × 50 mm) (Waters) and a gradient elution system based on 0.5% acetic acid in milliQ (A) and 100% acetonitrile (B) at a flow rate of 0.6 mL/min and the following method: initial concentration of 5% B (95% A), linear increase for 6.8 min until 95% B (5% A) and subsequent linear decrease to 5% B (95% A) during 1.8 min and finally maintaining these concentrations until the end of the run (10 min). Three commercially available benchmark non-ionic biosurfactants (i.e., di-rhamnolipid **BM1**, alkyl polyglucosides **BM2** and cocamidopropyl betaine **BM3)** with antimicrobial activity (Müller et al., 2017), (El-Sukkar et al., 2009) which are derived from palm oil and/or produced through chemical synthesis were chosen for comparative purposes. The benchmark surfactants were provided to in the context of the Applisurf project. An aqueous 25% (m/v) di-rhamnolipid solution was provided by the Pharmaceutical Science Research Group of the Biomedical Science Research Institute, Ulster University, UK. Liquid cocamidopropyl betaine (Euroquat HC47 VG) was provided by EOC Surfactants and liquid alkyl polyglucoside was provided by Flamac (Flanders Materials Centre). Ethanol, secondary amines, MeI, and Pd/C were purchased from Merck and Fisher Scientific and used without further purification. Dry acetonitrile was obtained using the MBraun SPS-800 solvent purification system.

### 2.2 Synthetic procedures

#### 2.2.1 Hydrogenated glycolipids (6–9)

Hydrogenation reactions were performed in a hydrogenator that *in-situ* generated H_2_ pressure of 5 bar. The C18:1 glycolipid was dissolved in 50 mL of methanol and (5% w) Pd/C was added under a N_2_ atmosphere. The reaction mixture was stirred under 5 bar H_2_ atmosphere for 18 h. Subsequently, the reaction mixture was filtered over Celite and concentrated under reduced pressure.

#### 2.2.2 Sophoroside alcohols (11)

In a 50 mL flask, 1 eq sophoroside aldehyde **10** was dissolved in demineralized water (15 mL). To this solution 1 eq of 2-picoline-borane was added. The mixture was acidified to pH 6 with acetic acid and stirred overnight at room temperature. The aqueous solution was washed three times with 15 mL of toluene and concentrated under reduced pressure. The sophoroside alcohols **11** were obtained as white powder.

#### 2.2.3 Sophoroside amines (12a–17a)

In a 300 mL Parr reactor, non-acetylated sophoroside aldehyde **10a** was dissolved in 40 mL of ethanol and 1 eq of the corresponding secondary amine (i.e., *N*-ethylmethylamine, *N*-butylmethylamine, *N*-hexylmethylamine, *N*-methyloctylamine, *N*-dodecylmethylamine and *N*-methyloctadecylamine) was added subsequently to obtain the sophoroside amines with the desired chain lengths. The reaction mixture was stirred for 30 min at room temperature. To this solution 5 w% Pd/C was added and the reaction mixture was stirred overnight (18 h) under 5 bar H_2_. The reaction mixture was filtered over celite and concentrated under reduced pressure. The non-acetylated sophoroside amines were obtained in high purity without further purification.

#### 2.2.4 Sophoroside quaternary salts (18a–23a)

In a 10 mL flame dried pressure resistant vial, non-acetylated sophoroside amine **12** was dissolved in dry acetonitrile. The solution was cooled down to 0°C and methyl iodide (1.2 eq.) was added. The vial was closed and heated to 80°C. After 24 h, the reaction mixture was cooled down to room temperature and the sophoroside quaternary ammonium salt was precipitated by the addition of diethyl ether. The precipitate was filtered off and dried under reduced pressure. The same procedure was repeated with each sophoroside amine (i.e. 12a to 17a) to obtain the desired sophoroside quaternary salt. In the absence of precipitation, the reaction mixture was concentrated under reduced pressure and recrystallized from diethyl ether if necessary. The sophoroside quaternary salts were obtained in high purity without further purification.

#### 2.2.5 Sophoroside amines (12b–17b) and sophoroside quaternary salts (18b–23b)

Synthesis of the acetylated sophoroside amines (**12b—17b**) and corresponding quaternary salts (**18b—23b**) were described in a previous study (Baccile et al., 2023).

### 2.3 Analytical characterization procedure

Nuclear Magnetic Resonance (NMR) spectra were recorded at 400 MHz (^1^H) and 100 MHz (^13^C) using a Bruker Avance III HD-400 MHz spectrometer at room temperature. The samples were dissolved in deuterated solvents (DMSO-d_6_). All spectra were processed using TOPSPIN 3.2 and peaks were assigned with the aid of 2D spectra (HSQC, H2BC and COSY). ^1^H and ^13^C chemical shifts (δ) are reported in parts per million (ppm) downfield of TMS and referenced to the residual solvent peak (DMSO-d_6_ δ_H_ = 2.50, δ_C_ = 39.52). High-resolution mass chromatograms were obtained with an Agilent 1,100 Series HPLC coupled to an Agilent 6,220 a time-of-flight (TOF) mass spectrometer equipped with ESI/APCI-multimode source.

### 2.4 Antimicrobial susceptibility testing

The antimicrobial activity of the compounds (listed in Table 1) was assessed against *B. subtilis* LMG 2099, *S. aureus* LMG 8224, *L. monocytogenes* LMG 23194, *E. coli* MG 1655, *P. aeruginosa* LMG 24986 and *C. albicans* SC 5314. The cultivation and testing method was described earlier by De Clercq et al. (De Clercq et al., 2021). Precultures of *E. coli, P. aeruginosa and B. subtilis* were grown in lysogeny broth (10 g L^−1^ tryptone, 5 g L^−1^ yeast extract, and 5 g L^−1^ NaCl) while *S. aureus* and *L. monocytogenes* were cultivated in brain heart infusion broth (Biokar diagnostics). *C. albicans* was cultivated in 3C medium (100 g L^−1^ glucose, 10 g L^−1^ yeast extract, and 1 g L^−1^ urea). The bacterial precultures were grown in test tubes containing 4 mL of broth at 37°C and 200 rpm for 24–36 h whereas *C. albicans* was grown in a test tube containing 4 mL of broth at 30°C and 200 rpm for 48–72 h.

The precultures were diluted in Mueller Hinton broth (MHB, Biokar diagnostics) to a turbidity of the 0.5 McFarland standard, which is approximately 1–2 × 10^8^ colony forming units (CFU) mL^−1^. For *L. monocytogenes*, BHI broth was used instead of MHB due to poor growth in MHB. Stock solutions of each compound were prepared at concentration of 200 g L^−1^ in MilliQ water or in dimethyl sulfoxide (DMSO) in case of poorly water soluble compounds. Therefore, a preliminary DMSO serial dilution test (up to 10% v/v) was used to evaluate microbial viability upon DMSO addition. The maximum concentration of the compounds tested per microbial strain was chosen based on the highest DMSO concentration that did not affect the growth of that strain. This maximum DMSO concentration was also included in the assay in parallel as an additional control for each strain. The minimum inhibitory concentration (MIC) that inhibits the growth of the microorganisms completely and the minimum lethal concentration (MBC) at which no growth could be observed after reinoculation were used as the measures of the antimicrobial activity. MIC and MBC values were determined in duplicates (n = 2) using a serial dilution method in 96-well microtiter plates (MTPs) based on Clinical and Laboratory Standards Institute (CLSI) guidelines (Weinstein et al., 2018; Elshikh et al., 2016; Elshikh et al., 2017). In case of a different result in between the replicates, the highest concentrations were taken into account. Concentrations of the compounds (i.e., microbially produced glycolipids, hydrogenated glycolipids, alcohol and aldehyde sophorosides) tested during the first screening ranged from 0.5 to 20 mg mL^−1^ while during the screening of the second set of compounds (i.e., sophoroside amines and sophoroside quaternary salts) the concentrations ranged from 5 × 10^−4^–10 mg mL^−1^. The final volume of the wells was 150 μL and 200 µL for the first and second screening, respectively. The pH of the MHB was buffered to a pH of 4 and a pH of 7 for *C. albicans* and for bacteria respectively using a citrate-phosphate buffer. Negative controls (non-inoculated microtiter wells) and positive controls, i.e., addition of antibiotics in known inhibition concentrations were also included in the screening: kanamycin (50 mg L^−1^) for *E*. *coli, B. subtilis, P*. *aeruginosa* and *S*. *aureus,* nystatin (2 mg L^−1^) and ampicillin (100 mg L^−1^) for *C*. *albicans* and *L. monocytogenes,* respectively (Lydon et al., 2017), (Missiakas and Schneewind, 2013), (Nenoff et al., 2016), (Roedel et al., 2019).

The plates were incubated for 24 h on a MTP shaker at 700 rpm at 30°C for yeasts and 37°C for bacteria. Before and after cultivation (24–36 h), the growth was monitored by optical density measurements at 600 nm by using a multilabel microtiter plate reader (FLUOstar OPTIMA FL, BMG Labtech). As (lactonic) SLs interfere with OD600 measurements, and some biosurfactants resulted in slightly turbid solutions at the highest concentrations, growth was also assessed using a resazurin assay. This fluorometric/colorimetric assay is based on the reduction of the blue dye resazurin (absorbance at 600 nm) with a formation of the red fluorescent dye resofurin (ex/em of 530–560 nm/590 nm) by metabolically active cells. In practice, 30 µL of a 0.2 g L^−1^ filter sterilized resazurin solution (Sigma-Aldrich) was added to the cell cultures in the MTP after 24 or 48 h of cultivation and was incubated for an additional 1–2 h. Microbial growth was verified by fluorescence measurements of resorufin at 560 nm/590 nm a multilabel microtiter plate reader (FLUOstar OPTIMA FL, BMG Labtech).

## 3 Results and discussion

### 3.1 Synthesis of new sophoroside derivatives

The C9 sophoroside aldehyde **10** was obtained according to the previously described procedure for ozonolysis of bola sophoroside **4** in water (Pala et al., 2022). The subsequent reduction of the SS aldehyde **10** with 2-picoline-borane yielded the SS alcohol **11** (Figure 2).

**FIGURE 3:**
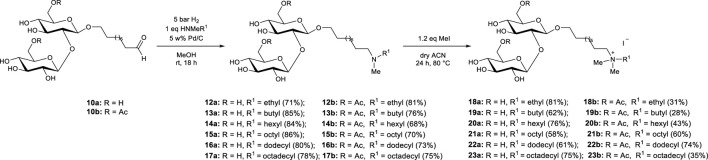
Synthesis of sophoroside amines 12—17 and sophoroside quaternary ammonium salts 18—23 starting from sophoroside aldehyde 10 obtained from bola sophorosides. The “a” suffix in the compound numbers represents nonacetylated analogues whereas the “b” suffix indicates the diacetylated analogues.

The set of SS amines **12–17** was synthesized by catalytic reductive amination of C9 SS aldehyde **10** with selected secondary amines using a reducing agent (Figure 3). To ensure the success of the reductive amination process an appropriate reducing agent that can selectively reduce imines over the SS aldehyde (Sato et al., 2004) was carefully selected. NaBH_3_CN has been commonly employed as a reducing agent for reductive amination because it can selectively reduce the imine in the presence of an aldehyde functionality (Delbeke et al., 2019). However, it is toxic and significant health risks associated with using it necessitate careful handling and disposal procedures. Moreover, the work-up stream may contain highly toxic cyanide salts. Thus, the toxicity and environmentally hazardous/unfriendly nature of this reducing agent makes it less attractive for this study in the context of a green synthesis. In contrast, molecular hydrogen is readily available, cost-effective and atom-economical as a reagent, producing only water as a by-product. Additionally, reduction with catalytic hydrogenation is simple, less toxic and more sustainable in comparison to reduction with agents such as borohydrides (Nishimura, 2001). Therefore, in this study, catalytic hydrogenation with H_2_ gas was preferred to obtain the desired SS amines. Six different secondary amines with ethyl, butyl, hexyl, octyl, dodecyl and octadecyl groups were selected to synthesize the desired SS amines **12–17**. As such, the influence of the alkyl chain length on the nitrogen atom on the antimicrobial properties of the obtained compounds could be evaluated. Initially, the SS aldehyde **10** and the secondary amine were mixed in ethanol to allow the formation of the intermediate SS imine. Subsequently, the SS imine was reduced to SS amine via catalytic hydrogenation. Next, quaternary ammonium sophorosides (SS quats) **18–23** were obtained via quaternization of the SS amine **12–17** with 1.2 eq. methyl iodide (Figure 3). Even though the synthesized SS amines showed low solubility in dry acetonitrile, the complete conversion was observed after 24 h. Methanol was also tested as an alternative reaction medium, but resulted in a significantly lower reaction rate, requiring subsequent addition of methyl iodide to achieve a complete conversion.

### 3.2 Evaluation of the antimicrobial activity

**FIGURE 4:**
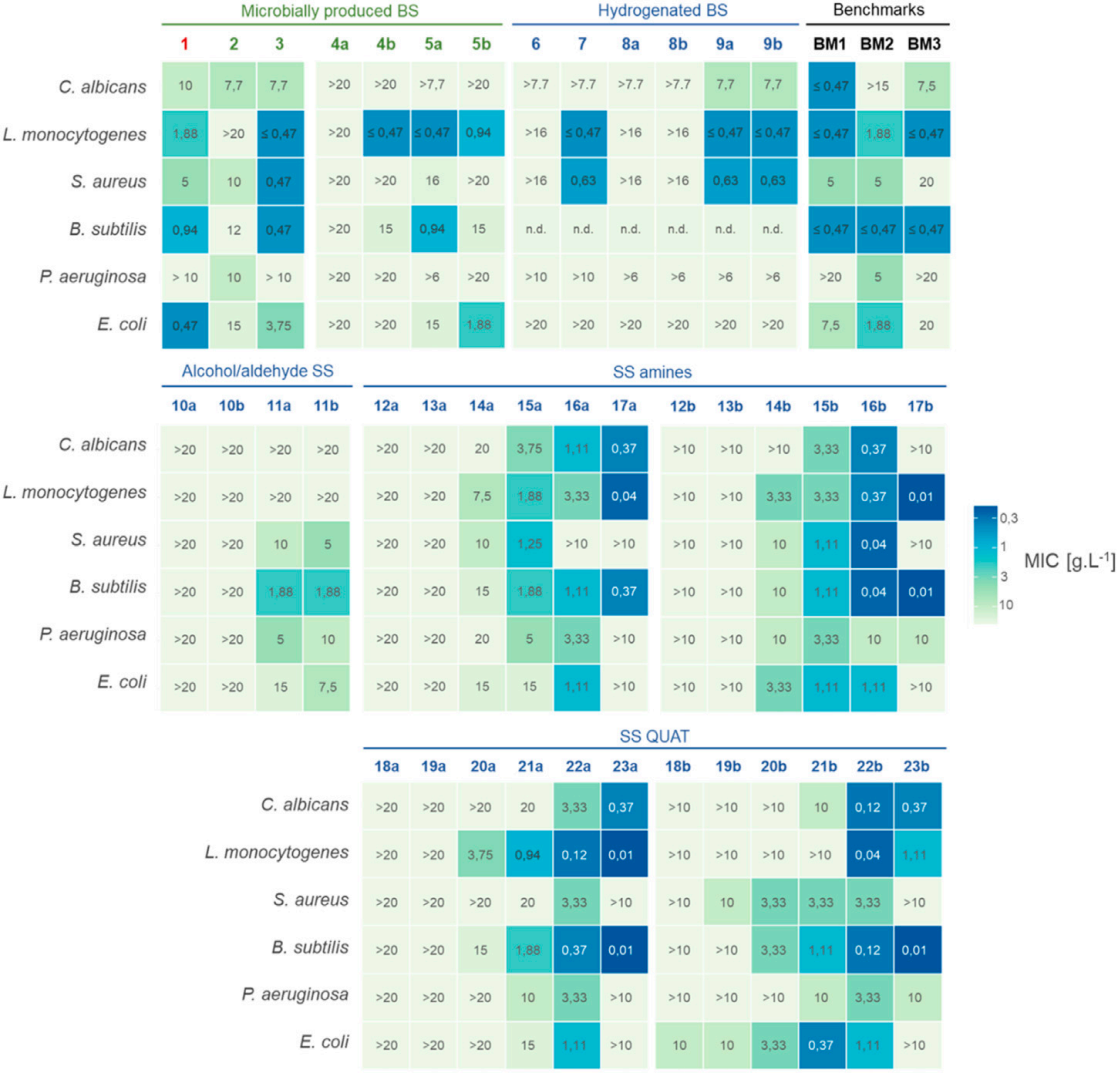
The figure depicts a heatmap showing the minimum inhibitory concentrations (MIC) in mg mL^−1^ for various mixtures, including a wild type mixture 1, microbially produced biosurfactants 2–5, hydrogentated glycolipids 6–9, chemically modified sophorosides 10—23 (a: nonacetylated, b: acetylated) and benchmark biosurfactants. The colors represent the level of antimicrobial activity, with darker blue indicating a lower MIC and thus higher activity. Additional information about the hydrophobic chain length of the biosurfactants can be found in Table 1. The values reported were observed for each replicate (n = 2). See Supplementary Table S1 for minimal bactericidal concentrations (MBC) in mg mL^−1^. Values marked with a “>” show combinations for which no exact MIC value could be determined within the tested concentration range (i.e., maximum tested concentration did not cause antimicrobial effects). Values marked with a “<” show combinations for which the MIC value is lower than the lowest tested concentration. Combinations where no conclusive MIC value could be determined are shown by “n.d”.

The antimicrobial activity of the microbially produced biosurfactants **1–5** and their derivatives **6–23** was evaluated against clinically relevant test organisms including the Gram-positive bacteria *B. subtilis*, *S. aureus* and *L. monocytogenes*, the Gram-negative bacteria *E. coli* and *P. aeruginosa*, and the yeast species *C. albicans*. Both minimum inhibitory concentrations (MICs) and minimum bactericidal/lethal concentrations (MBCs) were determined for all the compounds listed in Table 1 and compared to three different benchmark surfactants (**BM1–BM3**). The MIC and MBC values are provided in Supplementary Tables S1, S2 in the Supplementary Material. Additionally, a heatmap showing MICs in mg mL^−1^ is displayed in Figure 4. No microbial growth was observed after 24 h in the negative controls (non-inoculated MTP wells). The results of the antimicrobial activity tests will be discussed for two different groups (i.e., compounds **1–9** and compounds **10–23**) in the following sections.

### 3.3 Inhibitory activity (MIC) of (hydrogenated) microbially produced surfactants (1–9)

MIC values of the wild-type SLs range between 0.47 mg mL^−1^ and 10 mg mL^−1^ for all the test organisms, except for the wild type mixture **1** and lactonic SL **3** against *P. aeruginosa* (i.e., no MIC value could be determined up to 10 mg mL^−1^) (Supplementary Table S1, ESI). The latter is in accordance with literature, where the possibility of SLs inhibiting *P. aeruginosa* at concentrations of 1% v/v and higher is shown (Díaz De Rienzo et al., 2016). These observations also fit well with previously reported antimicrobial activity of the wild type SL mixture **1** against a wide range of micro-organisms and with the natural antimicrobial action of SLs as secondary metabolites in the *Starmerella* niche (De Clercq et al., 2021). The wild-type *S. bombicola* mixture contains both acidic **2** and lactonic **3** SLs. It is generally recognized that cell death is caused by integration of the hydrophobic chain of the surfactant into the cell membrane, leading to solubilization thereof (Dusane et al., 2012), (Dengle-Pulate et al., 2014). This theory supports the motivation that the more hydrophobic lactonic compound **3** possesses the highest antimicrobial activity of the two, in accordance with our results. The MIC values for the lactonic SL **3** for *L. monocytogenes* (0.47 mg mL^−1^)*, S. aureus* (0.47 mg mL^−1^)*, B. subtilis* (<0.47 mg mL^−1^) and *E. coli* (3.75 mg mL^−1^) are lower, or similar in the case of *C. albicans* (7.70 mg^−1^) than for the acidic sophorolipid **2**. The critical micellar concentration (CMC) (Supplementary Table S3, ESI) of lactonic SL **3** (45.1 mg L^−1^) is much smaller than for the acidic SL **2** (312 mg L^−1^). Therefore, the amount of free surfactants is expected to be higher for acidic SL **2** compared to lactonic SL **3**. The latter could explain the fact that antimicrobial activity is observed for the acidic SL **2** despite of its high hydrophilicity. The lower MIC value of the wild-type mixture against *E. coli* (0.47 mg mL^−1^), compared to the isolated acidic **2** or lactonic SL **3**, does not rule out more complex, synergistic effects when multiple surfactant species co-exist in solution. For example, we have observed that the solubility of lactonic SLs can be increased in the presence of highly water-soluble acidic SLs during our preliminary tests (in-house data). This would suggest higher MIC values for wild-type mixtures. However, for *E. coli*, lower MICs are observed for the wild-type mixture. It could also be hypothesised that acidic SL, consisting of a hydrophilic sophorose head and a hydrophilic carboxyl function at the other end of its structure, thus resembling a bola form, work as a delivery system of lactonic SLs to the membrane structure, explaining the lower MICs observed. It is however clear that further research is warranted to fully elucidate these phenomena.

A negative correlation between water solubility and antimicrobial activity is also noticeable when bola SSs **4** are considered. These compounds are extremely water-soluble (>500 mg mL^−1^ in H_2_O in a pH range from 2 to 10, (Supplementary Table S3, ESI)), while at the same time they are inactive against all test organisms in the concentration ranges tested, except for the acetylated bola SS **4b** against *L. monocytogenes* (0.47 mg mL^−1^) and *B. subtilis* (15 mg mL^−1^). Considering the difference in the CMC of the non-acetylated bola SS **4a** (224 mg L^−1^) and acetylated bola SS **4b** (63 mg L^−1^), it seems that the difference in the number of free molecules in solution appears to be of secondary importance for the antimicrobial effect for bola SSs.

The acidic glucolipids (**5a** and **5b**) showed antimicrobial activity against most of the tested organisms in the concentration range tested. Their lower water solubility (due to the presence of only one saccharide unit) and high CMC values (2,245 mg L^−1^) could support the antimicrobial activity. However, when the acidic GL **5a** and acidic SL **2** are compared, the removal of the glucose moiety does not result in a consistent increased microbial activity for all tested microorganisms. In another study, the acidic GLs even displayed lower killing efficacy compared more hydrophilic acidic SL due to less effective interactions with their molecular environment, such as glucose and protein moieties in the cytoplasmatic membrane (Valotteau et al., 2015).

Saturation of the hydrophobic aliphatic chain of microbially produced biosurfactants, to form hydrogenated compounds (**6–9**), can result in altered MIC values, but not for all compounds. Saturated acidic **6** and lactonic **7** SLs did not have better antimicrobial properties compared to their unsaturated analogues (**2** and **3**), respectively. Hydrogenation resulted in higher antimicrobial activity when considering C18:0 acidic glucolipids **9a** and **9b** compared to **5a** and **5b**, but this is not the case for *E. coli*. Clearly, and as already indicated above and as repeated below, the complex nature of amphiphilic compounds and the macromolecular structures of surfactants together with complex interactions with cellular systems necessitate a more in-depth investigation to reveal the structural features that mainly determine antimicrobial activity.

In this study, both wild-type SLs (**1–3**) and novel microbially produced glycolipids (**4–5**) together with their hydrogenated counterparts showed selective behaviour, implied by different MIC values against different microorganisms. For example, the mix of acetylated glucolipid **5b** inhibited *E. coli* and *L. monocytogenes* in a 1 mg mL^−1^ concentration, while the MIC values for other test organisms were rather high (e.g., 15 mg mL^−1^ for *B. subtilis*) or even above the highest concentration tested in this study (>20 mg mL^−1^). Similar observations can be made for the acetylated bola SS **4b**. In the case of hydrogenated lactonic **7** and acidic SLs **9**, a higher antimicrobial effect was observed for *L. monocytogenes* and *S. aureus* compared to the other test organisms. This selective activity is undesired for food, cosmetic and pharmaceutical applications where broad spectrum additives that inhibit a wide range of microorganisms are required to extend the shelf-life. On the other hand, this selective activity is an advantage in products where viable microorganisms are added as an active ingredient, such as probiotics for skin and gut health. It may also be preferred in cases where certain opportunistic pathogens need to be inhibited without disturbing the balance of a healthy microbiome. The selective behaviour of the wild-type SLs **1** and **3** and novel biosurfactants **4b, 5a, 5b, 7** and **9** sets these compounds apart from other biosurfactants such as di-rhamnolipids (**BM1**), alkyl polyglucosides (**BM2**) and cocamidopropyl betaine (**BM3**). While the antimicrobial activity of microbially produced surfactants is mainly selective and needs to be evaluated from one organism to another, **BM1** and **BM2** are perhaps more suited to be used as broad spectrum antimicrobials.

### 3.4 Inhibitory activity (MIC) of chemical derivatives SS amines and quats (10a–23a and 10b–23b)

The chemical modification of glycolipids provides an opportunity to substantially expand the existing glycolipid portfolio and to improve their biological activities (Delbeke et al., 2015b), (Pala et al., 2023). In this context, the chemically derived C9 SS alcohol and aldehyde (**10a–11a** and **10b–11b**), SS amines (**12a–17a** and **12b–17b**) and corresponding SS quats (**18a–23a** and **18b–23b**) were synthesized.

A significant difference in properties can already be noticed between the SS aldehyde (**10**) and SS alcohol (**11**). While no MIC values (MIC >20 mg mL^−1^) could be determined in the tested concentration range for SS aldehyde **10a - 10b**, the more polar SS alcohols **11a** (acetylated) and **11b** (non-acetylated) showed clear antimicrobial activities with MIC values against *S. aureus* of 10 and 5 mg mL^−1^ *,* against *P. aeruginosa* of 5 and 10 mg mL^−1^, against *E. coli* of 15 and 7.5 mg mL^−1^, respectively and against *B. subtilis* of 1.88 mg mL^−1^ for both **11a** and **11b**.

Among the SS amines, derivatives with long alkyl chains (**14a–17a, 14b–17b**) on the nitrogen atom show modest to high antimicrobial activities against all the tested microorganisms (Figure 4). High antimicrobial activities were obtained with *N*-octyl (**15a, 15b**), *N*-dodecyl (**16a, 16b**) and *N*-octadecyl (**17a**) derivatives against *C. albicans,* with the lowest MIC value of 0.37 mg mL^−1^ for the later. While *N*-hexyl to *N-*octadecyl (**14a–17a, 14b–17b**) SS amine derivatives inhibited the growth of *L. monocytogenes* and *B. subtilis*, *N-*octadecyl (**17a and 17b**) derivatives displayed the lowest MIC values in this group of SS derivatives with the MIC of 0.04 and 0.37 mg mL^−1^ (**17a** respectively) and of 0.01 mg mL^−1^ (**17b**) for both microorganisms. Regarding *S. aureus*, the *N*-dodecyl derivative (**16b**) showed the lowest MIC value (0.04 mg mL^−1^), not only in the SS amine group, but among all tested compounds in this study. It seems that the length of the alkyl chain on the nitrogen atom plays an important role in the inhibition of *S. aureus,* which could also be seen in literature (Delbeke et al., 2019), where *N*-dodecyl and *N*-pentadecyl derivatives of SS quats also had the lowest MIC values (1.71 μmol mL^−1^) of all tested derivatives. The highest activities within the SS amines against the Gram-negative bacteria (i.e., MIC values in between 10 and 1.11 mg mL^−1^) were also obtained with *N*-hexyl (**14b**)*, N*-octyl (**15a** and **15b**) and *N*-dodecyl (**16a** and **16b**) derivatives. Moreover, it was observed that slightly lower MIC values were obtained with the acetylated amines compared to non-acetylated SS amines.

It was expected that SS quats would display good antimicrobial activity at concentrations similar to the antibiotic gentamicin sulphate (Delbeke et al., 2015c). Indeed, several of the SS quats derivatives showed significant growth reduction compared to the control. MIC values equal or below 20 mg mL^−1^ were obtained for all tested microorganisms. Particularly, *N-*dodecyl (**22a** and **22b**) and *N-*octadecyl (**23a** and **23b**) SS quats for *C. albicans*; *N*-octyl (**21a**), *N-*dodecyl (**22a** and **22b**) and *N-*octadecyl (**23a** and **23b**) SS quats for *L. monocytogenes* and *B. subtilis* and *N*-octyl (**21b**) and (**22a** and **22b**) *N-*dodecyl for *E. coli* performed the best in between the SS quats. A comparison of the SS quats with the SS amines that have the same alkyl chain on the nitrogen atom indicates that the activities of the SS amines are similar to the SS quats, which was not yet described in literature. This is promising, as the tertiary amines are not often as toxic as the quaternary ammonium salts (Zhang et al., 2015).

Although a comparison remains delicate given the fact that microbially produced or hydrogenated glycolipids (**1**–**9**) were tested at rather limited concentration range, this does not undermine the encouraging results of high antimicrobial activity observed for some long chain SS amines (**16b, 17a** and **17b**) and quats (**22b, 23a** and **23b**), for which better antimicrobial activity compared to microbially produced glycolipids is certainly plausible. The antimicrobial activity of compounds **11a**, **11b**, **17a**, **17b** and **23a** is still microorganism-dependent. The other chemically modified SSs however could be classified as either being biologically active against the majority or even all of the test organisms (**14a**, **15a**, **15b**, **16a**, **16b**, **21a**, **21b**, **22a**, **22b**, **23b**) or as being mainly inactive SS derivatives (**10a**, **10b**, **12a**, **12b**, **13a**, **13b**, **18a**, **18b**, **19a**, **19b**). Thus, for the microbially SS derivatives where a certain antimicrobial activity was observed, this activity is exhibited against the majority, if not all, of the test organisms in the tested concentrations. Therefore and unlike the microbially produced glycolipids, where none of the compounds showed activity against all test organisms, some of the modified SS derivatives seem to display a trend of more broad range antimicrobial activity. Tang et al. reported a similar trend with the arginine SL conjugates (Tang et al., 2021). For instance, the arginine conjugated SL displayed efficient antimicrobial activities against all eight tested microorganisms (i.e., multiple strains of *B. subtilis*, *B. cereus*, *S. aureus*, *C. albicans* and *Moesziomyces* sp.) with MIC values between 31 and 250 mg L^−1^, while acidic SL inhibited the growth of only three (i.e., two *B. subtilis* strains and *B. cereus*) with MICs between 250 and 500 mg L^−1^.

Compounds **15a, 15b, 16a, 16b, 17a, 17b, 21a, 21b, 22a, 22b, 23a, 23b** distinguish themselves from the tested benchmark surfactants (BM1—BM3) due to their particularly high biological activity combined with the outspoken broad spectrum effects. In terms of absolute MIC values, minimum four and maximum nine (i.e., against *C. albicans* and *S. aureus*, respectively) of the ten aforementioned derivatives show an equal or even lower MIC value compared to the benchmark with the lowest MIC for each test organism. Furthermore, the SS amine and SS quaternary derivatives with *N*-octyl, *N*-dodecyl and *N*-octadecyl alkyl chains show comparable MIC values with benzalkonium chloride, a well-known synthetically produced quaternary ammonium surfactant used as preservative in pharmaceuticals. Perinelli et al. reported MIC values of 1, 4, 32 and 4 mg mL^−1^ against *S. aureus*, *E. coli*, *P. aeruginosa* and *C. albicans*, respectively (Perinelli et al., 2019). On the other hand, SS quats exhibited lower microbial activities against above-mentioned microorganisms in comparison to cetyltrimethylammonium bromide (Maneedaeng et al., 2018).

As explained above, it is generally recognized that cell death is caused by integration of the hydrophobic chain of the surfactant into the cell membrane, leading to altered membrane permeability and leakage of ions, ATP and other vital cytoplasmatic components, but also loss of the pH gradient and inactivation of membrane enzymes, eventually causing rupture of membrane and lysis of the cells (Dusane et al., 2012), (Dengle-Pulate et al., 2014), (Carmona-Ribeiro and Carrasco, 2013). This theory can also be supported here, by the lower absolute MIC values of the best performing -surfactants (**16b, 17a, 17b, 22a, 22b, 23a and 23b)** against the three Gram-positive bacteria tested (*B. subtilis, L. monocytogenes, S. aureus*) compared to the Gram-negative bacteria (*E. coli, P. aeruginosa*). We hypothesize that the outer membrane of the Gram-negative bacteria, which mainly consists of lipopolysaccharides and lipids, may act as a defensive barrier that withholds the amphiphilic molecules before they can drastically disrupt the cell-strengthening cytoplasmic membrane. Gram-positive organisms do not dispose of such an outer membrane, hence accumulation of the amphiphilic molecules will take place at the cytoplasmic membrane. Still, the cell envelope remains complex and next to the potential solubilization of the phospholipid cell membrane, the (periplasmatic) peptidoglycan layer will also affect translocation of surfactants towards the inner membrane. Few studies have explored surfactant interaction with the bacterial cell wall. Recent molecular dynamic simulations highlight the significance of a surfactant’s CMC and the associated number of free surfactants or small aggregates, influencing their translocation frequency and survival time (Sharma et al., 2022). The peptidoglycan cell wall serves more as a passive filter, more effectively controlling the translocation of surfactant aggregates than single surfactant molecules due to size differences. As indicated above, further exploration of supramolecular structures of the modified surfactants and their interaction with peptidoglycan and other cellular systems could enhance the preliminary retention models.

The increasing acetylation degree of the SS amines and quats generally has a positive impact on inhibitory concentrations. Additionally, a clear trend can be observed in the antimicrobial activity of the SS amines (**12a–17a and 12b–17b**) and SS quats (**18a–23a and 18b–23b**) with respect to the alkyl chain length attached to the nitrogen atom. The antimicrobial activity of both SS amines and quats were positively affected by the increased alkyl chain length. For example, SS derivatives with the *N-*octyl, *N-*dodecyl and *N-*octadecyl alkyl chains on the nitrogen atom showed higher antimicrobial activities against the Gram-positive and Gram-negative bacteria tested as well as the yeast (i.e., *C. albicans*)*.* The latter is consistent with what has been reported regarding the parabolic effect of MIC values as a function of increasing alkyl chain length (Delbeke et al., 2019), (Birnie et al., 2000). The hydrophobicity of the chain length affects the penetration into the lipid layer and therefore increases the free volume that is created in the lipid layer as well as the partitioning coefficient between the lipid and aqueous layers. Therefore, the aliphatic chain should be neither too long nor too short. The optimal chain length requires a balance between the ability to penetrate the membrane, the solubility and the free volume created into the lipid layer after penetration causing membrane thinning. This optimum chain length varies between different bacteria due to different properties of bacterial membrane lipids, such as different lipid chain length, degree of unsaturation and fluidity. However, from these results it could be concluded that at least a range of the alkyl chain length can be selected (i.e. 8 < n < 18) with optimal antimicrobial activity for all organisms tested. More specifically, based on the results of this study, the suggested alkyl chain length range could be 12 < n < 18 for *C. albicans*, 8 < n < 18 for *L. monocytogens* and 8 < n < 12 for *S. aureus*, *P. aeruginosa* as well as *E. coli*. Besides the hydrophobicity, the electrostatic interaction between the positively charged derivatives and negatively charged lipid layer plays a prominent role in the disruption of the cell membranes. Therefore, a balance should be found between the positive charge and the hydrophobicity as well.

### 3.5 Lethal activity (MBC)

Lethality of the compounds was investigated through determination of the minimum bactericidal concentration (MBC), for which values are shown in Supplementary Table S1 (ESI). *P. aeruginosa,* which was not lethally affected by any of the components at tested concentrations, is not represented in this table. In the case of *S. aureus*, lethal effects could only be observed for SS amine **16b** and SS quats **22a, 21b, 22b** with an MBC of at 10 mg mL^−1^. These compounds are the only ones able to approximate the lethal activity of the benchmarks (5 mg mL^−1^). In contrast to *P. aeruginosa* and *S. aureus*, *B. subtilis* has MBC values as low as 0.94 mg mL^−1^ for microbially produced SLs (**1, 3, 5a**) and 0.014—0.04 mg mL^−1^ in the case of SS amines (**16b, 17b**) and SS quats (**23a, 23b**). For the organisms tested, the MBC values are in the same concentration range with the MIC values. For the other test organisms (*C. albicans, E. coli, L. monocytogenes*) the MBC values of the microbially produced glycolipids were observably similar to or higher than the MIC values. For example, for *L. monocytogenes* the MBC values equal the MIC values (**1, 21a, 23b**), but typically the MBC amounted to values up to a factor of 10–100 (e.g., **17b**) higher than the MIC values. This corresponds to the higher MBC values also for *E. coli* and *C. albicans.* For *E. coli*, the lowest MBC values of 7–10 mg mL^−1^ could be observed for the aldehyde SS (**9b**) or long chain amine and quat SS (**15b, 16a, 16b, 22a**). For C. albicans, the lowest MBC values even reached 0.37—1.88 mg mL^−1^ with SS amines (**15a, 16b**) and SS quats (**23a, 22b, 23b**). The results show the ability of microbially produced glycolipids and especially chemical derivatives such as SS amines and quats, not only to inhibit growth, but also lethally affect subjected microorganisms. Although bacteriostatic activity will be sufficient in most applications to prevent starting or ongoing growth of food spoiling microorganisms, it is generally not satisfactory in agricultural and (bio)medical applications where sterilizing effects are crucial (Naughton et al., 2019). For these circumstances, the potential bactericidal effect of glycolipids shown here can be very useful.

## 4 Conclusion

The useful surface-active features and the green nature (high biodegradability, biobased production and mild properties) of wild-type sophorolipids have paved the way for the generation of green business-to-consumer products for cleaning. Although this green transition has already been initiated, the sophorolipid industry is still subject to relatively high prices (20–30 €/∙kg in Europe). Therefore, it is of utmost importance to discover promising and especially effective compounds that allow lower dosages to minimize their application costs. Also, the current production costs could be justified by increasing the structural diversity to enhance their properties and to pave the way towards further uses in high-value applications such as pharmaceuticals, personal care products, etc. In this regard, the structural variation of the sophorolipid platform has been increased via metabolic engineering efforts, chemical derivation or the successful combination thereof. In this study, microbially produced bola sophorosides were chemically modified into novel sophoroside amines and sophoroside quaternary ammonium salts via an intermediate sophoroside aldehyde. Additionally, the double bond on the microbial surfactants was saturated via catalytic hydrogenation. In the end, all available SS derivatives and microbially produced biosurfactants were evaluated in terms of antimicrobial properties.

In this study, both wild-type SLs and novel microbially produced glycolipids and the latter’s hydrogenated counterparts showed selective antimicrobial behaviour, implied by different MIC values against different microorganisms. Interestingly, the antimicrobial activity can considerably be altered after chemical modification, where a general trend towards broad-spectrum activity was observed. However, elucidating the structure-function relations is a complex process. Still, a trend can be seen among the SS amines and quaternary salts with increasing alkyl chain length, which might be due to its influence on the partitioning of the compound into the microbial membrane bilayer. Finding the optimal balance between the cationic charge and the hydrophobic chain length of SS amine and SS quats results in chemical derivatives that are effective towards all tested organisms, in concentrations as low as 0.0137 mg mL^−1^. Future research opportunities exist in fully complementing the existing models regarding antimicrobial mode of action and subsequently revealing the observed specificity of compounds. Factors such as steric hindrance due to surfactant or aggregate size, phase behaviour and interaction of supramolecular structures with the cell environment, the organism-specific cell envelope and the extracellular metabolome of test organisms may all impact the efficacy of these compounds in terms of biological activity. Following the determination of the end-application area, a techno-economic analysis would be required to clarify the necessity of the chemical modification of SLs/SSs. Nonetheless, the combination of the observed selective antimicrobial nature of the microbially produced glycolipids and the broad-spectrum activity of novel chemical derived surfactants has potential to be applied in a wide range of applications where specific inhibition or total inhibition is required. Even the applications where only physicochemical parameters are important while the antimicrobial activity needs to be avoided can be achieved by, for example, using the (un)saturated bola sophorosides or short-chain SS amines and SS quats (i.e., *N-*ethyl and *N-*butyl derivatives). In conclusion, the use of selective or broad-spectrum antimicrobial glycolipids with their beneficial physicochemical characteristics in various formulations such as pharmaceutical, medical, food and crop protective products cannot only reduce the number of required ingredients, but also lower the production costs, thus bringing sophorolipid containing products closer to commercialization. This research highlights that the strategic integration of biotechnology and chemistry enables precise customization of biosurfactants within the glycolipid portfolio, rendering them highly adaptable to meet diverse application needs.

## Data Availability

The original contributions presented in the study are included in the article/Supplementary Material, further inquiries can be directed to the corresponding authors.
