# Supplementary material for: Tuning the antimicrobial activity of microbial glycolipid biosurfactants through chemical modification
Source: Front Bioeng Biotechnol. 2024 Feb 14;12:1347185. doi: 10.3389/fbioe.2024.1347185 (PMC10900251; doi:10.3389/fbioe.2024.1347185)
Supplement: Supplementary file 1 [file DataSheet1.PDF]

Supporting information for:

Tuning the antimicrobial activity of microbial glycolipid biosurfactants through chemical modification

Melike Pala, [1,†] *Martijn Castelein* [2,†] *, Camille Dewaele*, *Sophie L. K. W. Roelants,* [2,3,*] *Wim Soetaert,* [2,3] *and Christian V. Stevens* [1,*]

[1]SynBioC, Department of Green Chemistry and Technology, Ghent University, Coupure Links 653, Ghent, Belgium

[2]InBio, Department of Biotechnology, Ghent University, Coupure Links 653, Ghent, Belgium

[3]Bio Base Europe Pilot Plant (BBEPP), Rodenhuizenkaai 1, 9042 Ghent (Desteldonk), Belgium

[†] These two authors contributed equally to this work and share first authorship.

Corresponding authors: Sophie L. K. W. Roelants, Christian V. Stevens

Study of minimum inhibitory concentrations (MICs) and minimal bactericidal concentrations (MBCs) of the test surfactants against the test organisms

Table 1. Minimum inhibitory concentrations (MIC) in mg mL^{-1} of microbially produced glycolipids 1 – 5, hydrogenated glycolipids 6 – 9, chemically modified sophorosides 10 – 23 and benchmark biosurfactant (i.e. BM1: Di-Rhamnolipids, BM2: Alkyl polyglucosides, BM3: Cocamidopropyl betaine) against *E. coli*, *P. aeruginosa*, *S. aureus*, *L. monocytogenes*, *B. subtilis*, *C. albicans*. Values marked with a ">" show combinations for which no exact MIC value could be determined within the tested concentration range (i.e. maximum tested concentration did not cause antimicrobial effects). Values marked with a "<" show combinations for which the MIC value is lower than the lowest tested concentration. Combinations where no conclusive MIC value could be determined are shown by "n.d".

#	Code	E. Coli	P. Aeruginosa	S. Aureus	L. Monocytogenes	B. Subtilis	C. Albicans
1	Wild type mix SL	0,47	>10	5	1,88	0,94	10
2	Nonac acidic SL C18:1	15	10	10	>20	>12	7,7
3	Ac lactonic SL (C18:1)	3,75	>10	0,47	0,47	0,5	7,7
4a	NonAc bola SS (C18:1)	>20	>20	>20	>20	>20	>20
4b	Ac bola SS (C18:1)	>20	>20	>20	0,47	15	>20
5a	NonAc acidic GL (C18:1)	15	>6	16	0,47	0,94	>7,7
5b	Ac acidic GL (C18:1)	1,88	>20	>20	0,94	15	>20
6	NonAc acidic SL (C18:0)	>20	>10	>16	>16	nd	>7,7
7	Ac lacton SL (C18:0)	>20	>10	0,63	0,47	nd	>7,7
8a	Nonac bola SS (C18:0)	>20	>6	>16	>16	nd	>7,7
8b	Ac bola SS (C18:0)	>20	>6	>16	>16	nd	>7,7
9a	NonAc acidic GL (C18:0)	>20	>6	0,63	0,47	nd	>7,7
9b	Ac acidic GL (C18:0)	>20	>6	0,63	0,47	nd	>7,7
10a	NonAc aldehyde SS (C9:0)	>20	>20	>20	>20	>20	>20
10b	Ac aldehyde SS (C9:0)	>20	>20	>20	>20	>20	>20
11a	NonAc alcohol SS (C9:0)	15	5	10	>20	1,88	>20
11b	Ac alcohol SS (C9:0)	7,5	10	5	>20	1,88	>20
12a	Nonac SS amine C2	>20	>20	>20	>20	>20	>20
13a	Nonac SS amine C4	>20	>20	>20	>20	>20	>20
14a	Nonac SS amine C6	15	20	20	20	>20	>20
15a	Nonac SS amine C8	15	5	1,25	7,5	1,88	3,75
16a	Nonac SS amine C12	1,11	3,33	3,33	3,33	1,11	1,11
17a	Nonac SS amine C18	>10	>10	>10	0,04	0,37	0,37
18a	Nonac SS quat C2	>20	>20	>20	>20	>20	>20
19a	Nonac SS quat C4	>20	>20	>20	>20	>20	>20
20a	Nonac SS quat C6	>20	>20	>20	3,75	15	>20
21a	Nonac SS quat C8	15	10	20	0,94	1,88	20
22a	Nonac SS quat C12	1,11	3,33	3,33	0,12	0,37	3,33
23a	Nonac SS quat C18	>10	>10	>10	0,0	0,0	0,37
12b	Ac SS amine C2	>10	>10	>10	>10	>10	>10
13b	Ac SS amine C4	>10	>10	>10	>10	>10	>10
14b	Ac SS amine C6	3,33	10	10	3,33	10	>10
15b	Ac SS amine C8	1,11	3,33	1,11	3,33	1,11	3,33
16b	Ac SS amine C12	1,11	10	0,04	0,37	0,04	0,37
17b	Ac SS amine C18	>10	10	>10	0,01	0,01	>10
18b	Ac SS quat C2	10	>10	>10	>10	>10	>10
19b	Ac SS quat C4	10	>10	10	>10	>10	>10
20b	Ac SS quat C6	3,33	>10	3,33	>10	3,33	>10
21b	Ac SS quat C8	0,37	10	3,33	>10	1,11	10
22b	Ac SS quat C12	1,11	3,33	3,33	0,04	0,12	0,12
23b	Ac SS quat C18	>10	10	>10	1,11	0,01	0,37
BM1	Di-Rhamnolipid	7,50	>20	5	0,47	0,47	0,47
BM2	Alkyl polyglucosides	1,88	5,00	5	1,88	0,47	>15
BM3	Cocamidopropyl betaine	20	>20	20	0,47	0,47	7,50

Table 2. Minimal bactericidal concentrations (MBC) in mg mL^{-1}. *Pseudomonas aeruginosa* is not included in this table as no lethal concentrations were detected. Components where no lethal concentrations were detected are also not presented in this table. N.d. = no data available, SS = sophoroside, QUAT = quaternary ammonium salt, SL = sophorolipid, GL = glucolipid.

	Code	Biosurfactant	*Escherichia coli*	*Bacillus subtilis*	*Staphylococcus aureus*	*Listeria monocytogenes*	*Candida albicans*
**Microbially produced glycolipids**							
	Wild type sophorolipid compounds						
1	WT	Wild type mix SL (acidic and lactonic SL) [1]	15	0.94	>16	1.88	>7
2	NonAc acidic SL (C18:1)	Non-acetylated acidic SL	15	7.5	>16	1.88	>7
3	Ac lacton SL (C18:1)	Acetylated lactonic SL	15	0.94	n.d.	15	>7
	New-to-nature sophoroside compounds						
5a	NonAc acidic GL (C18:1)	Non-acetylated GL	>15	0.94	>16	7.5	>7.7
5b	Ac acidic GL (C18:1)	Mix acetylated GL	>15	15	>20	15	>20
**Chemical derivatives**							
	Hydrogenated BS						
7	Ac lacton SL (C18:0)	Acetylated lactonic SL	>20	>20	>20	0.94	>20
9a	NonAc acidic GL (C18:0)	Non-acetylated GL	>20	>20	>20	1.88	>20
9b	Ac acidic GL (C18:0)	Mix acetylated GL	>20	>20	>20	1.88	>20
	Short chain intermediates						
10a	NonAc aldehyde SS (C9:0)	Non-acetylated aldehyde	>15	7.5	>20	>20	15
10b	Ac aldehyde SS (C9:0)	Acetylated aldehyde	7.5	1.88	>20	>20	15
	Sophoroside amine derivatives						
14a	NonAc SS amine (C6)	Non-acetylated Amine	>15	15	>20	>15	7.5
15a	NonAc SS amine (C8)	Non-acetylated Amine	15	1.88	>20	15	1.88
16a	NonAc SS amine (C12)	Non-acetylated Amine	10	1.11	>20	10	>10
17a	NonAc SS amine (C18)	Non-acetylated Amine	15	0.37	>20	0.37	>10
12b	Ac SS amine (C2)	Non-acetylated Amine	>10	>10	>10	>10	>10
13b	Ac SS amine (C4)	Non-acetylated Amine	>10	>10	>10	>10	>10
14b	Ac SS amine (C6)	Non-acetylated Amine	>10	10	>10	>10	>10
15b	Ac SS amine (C8)	Non-acetylated Amine	10	1.11	>10	>10	10
16b	Ac SS amine (C12)	Non-acetylated Amine	10	0.04	10	10	1.11
17b	Ac SS amine (C18)	Non-acetylated Amine	>10	0.014	>10	10	>10
	Sophoroside quaternary ammonium salt derivatives						
21a	NonAc SS quat (C8)	Non-acetylated QUAT	>20	>20	>20	0.94	>20
22a	NonAc SS quat (C12)	Non-acetylated QUAT	10	0.37	10	10	>10
23a	NonAc SS quat (C18)	Non-acetylated QUAT	>20	0.014	>20	3.33	0.37
18b	Ac SS quat (C2)	Non-acetylated quat	>10	>10	>10	>10	>10
19b	Ac SS quat (C4)	Non-acetylated quat	>10	>10	>10	>10	>10
20b	Ac SS quat (C6)	Non-acetylated quat	>10	3.33	>10	>10	>10
21b	Ac SS quat (C8)	Non-acetylated quat	>10	1.11	10	>10	>10
22b	Ac SS quat (C12)	Non-acetylated quat	>10	0.12	10	10	0.37
23b	Ac SS quat (C18)	Non-acetylated quat	>10	0.014	>10	0.37	1.11
Benchmarks							
BM1		Di-Rhamnolipid	>15	0.47	>20	15	>20
BM2		Alkyl polyglucosides	>15	0.47	5	1.88	3.75
BM3		Cocamidopropyl betaine	15	0.47	5	>15	0.47

[1] Consisting out of 60% lactonic sophorolipids and 40% acidic sophorolipids.

Table 3: Overview of solubility (g L^{-1}) and critical micellar concentrations CMC (in mg L-1) of the microbial glycolipids for non-controlled (NC), or pH controlled in between brackets. Solubility of the various glycolipids from the available portfolio in water and buffered water (pH 2, 6 and 10). The numbers indicate the highest concentration that was soluble, and the lowest concentration that was insoluble. CMC was determined based on surface tension measurements: Surface tension γ was measured by the Pendant Drop method using an optical tensiometer Theta Attension (Biolin Scientific) and the OneAttension software. The surface tension γ (mN/m) was plotted as a function of the logarithm of the surfactant concentration (log M). To determine the CMC, the slopes of the curve before and after the break occurring to the CMC are taken, and their intersection pinpoints the CMC and surface tension at CMC (γCMC). From CMC onwards, the surface tension γ remains constant. The GL characteristics were only determined at pH 9, due to poor solubility.

	**Code**	**Glycolipid**	**CMC (mg/L)**	**Solubility (g/L)**			
				H_2O	pH 2	pH 6	pH 10
1	WT	Wild type mix SL[2]	-	-	-	-	-
2	NonAc acidic SL (C18:1)	Non-acetylated acidic SL	312.2 (pH 5: 557.1)	> 500	> 500	> 500	> 500
3	Ac lactonic SL (C18:1)	Acetylated lactonic SL	45.1	1 – 2	1 – 5	1 – 5	0.1 – 1
4a	NonAc bola SS (C18:1)	Non-acetylated bola SS	224.5	> 500	> 500	> 500	> 500
4b	Ac bola SS (C18:1)	Acetylated bola SS	62.54	> 500	> 500	> 500	> 500
5a	NonAc acidic GL (C18:1)	Non-acetylated GL	(pH 9: 2245.2)	0.1 - 1	0.1 - 1	0.1 - 1	30 - 50
5b	Ac acidic GL (C18:1)	Mix acetylated GL	(pH 9: 58.9)	0.1 - 1	0.1 - 1	0.1 - 1	50 - 60

1. Consisting of 60% lactonic sophorolipids and 40% acidic sophorolipids.

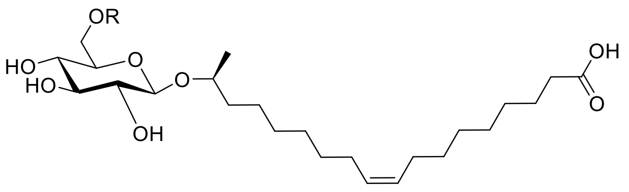

5a : R = H
5b : R = Ac

Figure 1. Chemical structures of nonacetylated (5a) and acetylated (5b) acidic glucolipids

NMR Characterization and spectra of the compounds

Non-acetylated acidic sophorolipid C18:0 (6):

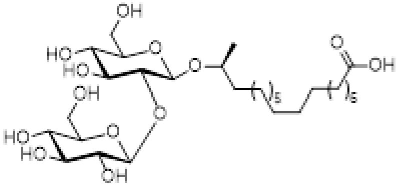

^{1}H NMR (400 MHz, MeOD): δ_H 1.15 (3H, d, J = 6.3 Hz, $C\underline{H}_3CH$), 1.16-1.31 (25H, m, 12x$C\underline{H}_2(CH_2)_2$, $C\underline{H}_aH_bCHCH_3$), 1.42-1.59 (3H, m, $C\underline{H}_2CH_2COOH$, $CH_a\underline{H}_bCHCH_3$), 2.17 (2H, t, J = 7.3 Hz, $C\underline{H}_2COOH$), 3.09-3.23 (3H, m, $C^{2''}\underline{H}$, 2x$C\underline{H}OC$), 3.23-3.39 (2H, m, $C\underline{H}OC$, $C^{3''}\underline{H}$), 3.32-3.40 (1H, $C^{2'}\underline{H}$), 3.40-3.49 (2H, m, $C\underline{H}OC$, $C^{3'}\underline{H}$), 3.51-3.61 (2H, m, $C\underline{H}_aH_bOH$), 3.69-3.80 (3H, m, $CH_3\underline{C}HCH_2$, 2x$CH_a\underline{H}_bOH$), 4.35 (1H, d, J = 7.9 Hz, $C^1\underline{H}$), 4.54 (1H, d, J = 7.8 Hz, $C^{1''}\underline{H}$). **^{13}C NMR** (100 MHz, MeOD): δ_C 20.5 ($\underline{C}H_3CH$), 24.7 ($\underline{C}H_2CH_2(C{=}O)OH$), 24.9 ($\underline{C}H_2(CH_2)_2$), 28.8-29.5 (11x$\underline{C}H_2(CH_2)_2$), 33.6 ($\underline{C}H_2COOH$), 36.4 ($\underline{C}H_2CHCH_3$), 61.3 ($\underline{C}H_2OH$), 61.6 ($CH_2OH$), 70.1 ($\underline{C}HOC$), 70.4 ($\underline{C}HOC$), 74.4 ($\underline{C}^{2''}H$), 76.3 ($\underline{C}HOC$), 76.4 ($\underline{C}^{3''}H$), 76.8 ($\underline{C}^{3'}H$), 76.9 ($\underline{C}HOC$), 77.4 ($CH_3\underline{C}HCH_2$), 80.5 ($\underline{C}^{2'}H$), 101.3 ($\underline{C}^{1'}H$), 103.2 ($\underline{C}^{1''}H$), 176.4 ($\underline{C}OOH$).

Acetylated lactonic sophorolipid ω-1 C18:0 (7):

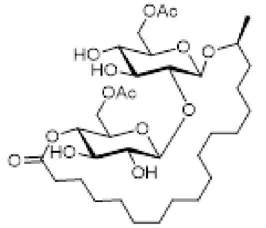

^{1}H NMR (400 MHz, DMSO): δ_H 1.11 (3H, d, J = 5.9 Hz, $C\underline{H}_3CH$), 1.16-1.32 (24H, m, 11x$CH_2(CH_2)_2$, $C\underline{H}_2CH_2CHCH_3$), 1.33-1.41 (1H, m, $C\underline{H}_aH_bCHCH_3$), 1.40-1.52 (2H, m, $C\underline{H}_aH_bCH_2C{=}O$, $CH_a\underline{H}_bCHCH_3$), 1.45-1.65 (1H, m, $CH_a\underline{H}_bCH_2C{=}O$), 2.00 (6H, s, 2x$C\underline{H}_3C{=}O$), 2.32 (2H, t, J = 6.3 Hz, $C\underline{H}_2C{=}O$), 3.05-3.16 (2H, m, $C\underline{H}OC$, $C^{3'}\underline{H}$), 3.17-3.26 (1H, m, $C^{2'}\underline{H}$), 3.34-3.49 (3H, m, 2X$C\underline{H}OC$, $C^{3''}\underline{H}$), 3.56-3.66 (2H, m, $C\underline{H}OC$, $C\underline{H}CH_3$), 3.98 (2H, d, J = 3.6 Hz, $C\underline{H}_2OAc$), 4.05 (1H, dxd, J = 11.9 Hz, J = 7.2 Hz, $C\underline{H}_aH_bOAc$), 4.23 (1H, m, $CH_a\underline{H}_bOAc$), 4.37 (1H, d, J = 7.9 Hz, $C^{1'}\underline{H}$), 4.52 (1H, d, J = 7.8 Hz, $C^{1''}\underline{H}$), 4.67 (1H, t, J = 9.7 Hz, $C\underline{H}OC$). **^{13}C NMR** (100 MHz, DMSO): δ_C 20.9 ($\underline{C}H_3C{=}O$), 21.1 ($\underline{C}H_3C{=}O$), 21.7 ($\underline{C}H_3CH$), 24.1 ($\underline{C}H_2CH_2C{=}O$), 25.0 ($\underline{C}H_2CH_2CHCH_3$), 27.5 ($\underline{C}H_2(CH_2)_2$), 27.6 ($\underline{C}H_2(CH_2)_2$), 27.8 (2X$\underline{C}H_2(CH_2)_2$), 28.0 ($\underline{C}H_2(CH_2)_2$), 28.6 ($\underline{C}H_2(CH_2)_2$), 28.8 ($\underline{C}H_2(CH_2)_2$), 29.0 ($\underline{C}H_2(CH_2)_2$), 29.5 (2x$\underline{C}H_2(CH_2)_2$), 29.7 ($\underline{C}H_2(CH_2)_2$), 30.2 ($\underline{C}H_2(CH_2)_2$), 33.7 ($\underline{C}H_2(C{=}O)OH$), 37.4 ($\underline{C}H_2CHCH_3$), 62.8 ($\underline{C}H_2OAc$), 64.0 ($\underline{C}H_2OAc$), 70.4 ($CHO\underline{C}$), 71.0 ($\underline{C}HOC$), 71.7 ($\underline{C}HOC$), 73.4 ($\underline{C}HOC$), 73.7 ($\underline{C}HOC$), 75.6 ($\underline{C}^{3'}H$), 76.5 ($C^{3''}H$), 77.8 ($\underline{C}HCH_3$), 83.3 ($C^{2'}H$), 102.1 ($\underline{C}^{1'}H$), 104.7 ($\underline{C}^{1''}H$), 170.2 ($\underline{C}H_3C{=}O$), 170.6 ($\underline{C}H_3C{=}O$), 172.5 ($H\underline{C}{=}O$).

Non-acetylated bola Sophoroside C18:0 (8a):

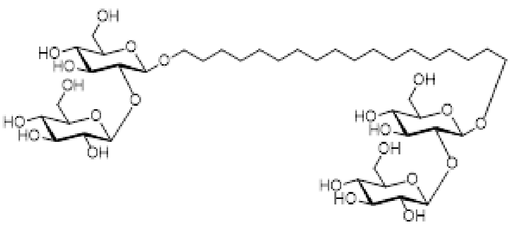

^{1}H NMR (400 MHz, DMSO): δ_H 1.12-1.36 (28H, m, $14xC\underline{H}_2(CH_2)_2$), 1.43-1.58 (4H, m, $2xCHOCH_2C\underline{H}_2$), 2.95-3.03 (2H, m, $2xC^{2''}\underline{H}$), 3.02-3.19 (10H, m, $4xC\underline{H}OC$, $4xC\underline{H}CH_2OH$, $2xC^{3''}\underline{H}$), 3.20-3.25 (2H, $2xC^{2'}\underline{H}$), 3.32-3.39 (2H, m, $2xC^{3'}\underline{H}$), 3.40-3.54 (6H, m, $4xC\underline{H}_aH_bOH$, $2xCHOC\underline{H}_a H_bCH_2$), 3.56-3.70 (4H, m, $C\underline{H}_aH_bOH$), 3.56-3.70 (4H, m, $4xCH_a\underline{H}_bOH$), 3.71-3.82 (2H, m, $2xCHOCH_a\underline{H}_bCH_2$), 4.27 (2H, d, J = 7.6 Hz, $2xC^{1'}\underline{H}$), 4.33 (2H, t, J = 5.8 Hz, $CHCH_2O\underline{H}$), 4.37 (2H, d, J = 7.5 Hz, $2xC^{1''}\underline{H}$), 4.51 (2H, t, J = 5.8 Hz, $CHCH_2OH$), 4.87 (4H, d, J = 3.9 Hz, $O\underline{H}$), 4.94 (2H, d, J = 3.1 Hz, $O\underline{H}$), 5.05 (2H, d, J = 3.4 Hz, OH), 5.17 (2H, d, J = 3.4 Hz, OH) 5.43 (2H, d, J = 3.4 Hz, $2xO\underline{H}$). **^{13}C NMR** (100 MHz, DMSO): δ_C 25.9 ($2x\underline{C}H_2(CH_2)_2$), 29.3-29.8 ($14x\underline{C}H_2(CH_2)_2$), 61.30 ($2x\underline{C}H_2OH$), 61.36 ($2x\underline{C}H_2OH$), 69.1 ($2xCHO\underline{C}H_2CH_2$), 70.20 ($2x\underline{C}HOC$), 70.22 ($2x\underline{C}HOC$), 75.3 ($2x\underline{C}^{2''}H$), 76.4 ($2x\underline{C}^{3'}H$), 76.5 ($2x\underline{C}^{3''}H$), 77.0 ($2x\underline{C}HOC$), 77.4 ($2x\underline{C}HOC$), 82.7 ($2x\underline{C}^{2'}H$), 101.7 ($2x\underline{C}^{1'}H$), 104.5 ($2x\underline{C}^{1''}H$).

Tetra-acetylated bola sophoroside C18:0 (8b):

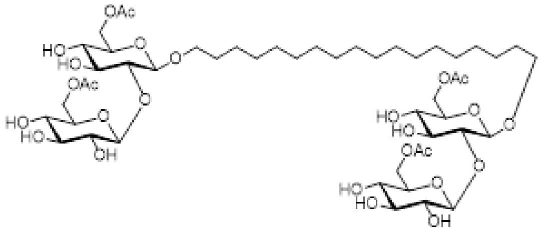

^{1}H NMR (400 MHz, DMSO): δ_H 1.15-1.33 (28H, m, $14xC\underline{H}_2(CH_2)_2$), 1.43-1.54 (4H, m, $2xCHOCH_2C\underline{H}_2$), 2.01 (6H, s, $2xC\underline{H}_3C{=}O$), 2.02 (6H, s, $2xC\underline{H}_3C{=}O$), 3.02 (2H, txd, J = 8.9, 3.4 Hz, $2xC^{2''}\underline{H}$), 3.07-3.21 (6H, m, $4xC\underline{H}OC$, $2xC^{3''}\underline{H}$), 3.21-3.25 (2H, dxd, J = 7.9, 6.8 Hz, $2XC^{2'}\underline{H}$), 3.30-3.35 (2H, m, $2xC\underline{H}OC$), 3.36-3.44 (4H, m, $2xC\underline{H}OC$, $2xC^{3'}\underline{H}$), 3.44-3.48 (2H, m, $2xCHOC\underline{H}_aH_bCH_2$), 3.62-3.71 (2H, m, $2xCHOCH_a\underline{H}_bCH_2$), 3.99-4.08 (4H, m, $4xC\underline{H}_aH_bOAc$), 4.18-4.30 (4H, m, $4xCH_a\underline{H}_bOAc$), 4.32 (2H, d, J = 7.8 Hz, $2xC^{1'}\underline{H}$), 4.41 (2H, d, J = 7.7 Hz, $2xC^{1''}\underline{H}$), 5.07 (2H, d, J = 4.7 Hz, $2xO\underline{H}$), 5.16 (2H, d, J = 5.8 Hz, $2xO\underline{H}$), 5.31 (2H, d, J = 5.8 Hz, $2xO\underline{H}$), 5.33 (2H, d, J = 3.6 Hz, $2xO\underline{H}$), 5.50 (2H, d, J = 3.5 Hz, $2xO\underline{H}$). **^{13}C NMR** (100 MHz, DMSO): δ_C 21.1 ($4X\underline{C}H_3C{=}O$), 25.8 ($2x\underline{C}H_2(CH_2)_2$), 29.1-29.8 ($14x\underline{C}H_2(CH_2)_2$), 63.9 ($2x\underline{C}H_2OAc$), 64.2 ($2x\underline{C}H_2OAc$), 69.0 ($2xCHO\underline{C}H_2CH_2$), 70.1 ($2x\underline{C}HOC$), 70.2 ($2x\underline{C}HOC$), 73.8 ($2x\underline{C}HOC$), 74.3 ($2x\underline{C}HOC$), 75.2 ($2x\underline{C}^{2''}H$), 76.2 ($2x\underline{C}^{3'}H$), 76.3 ($2x\underline{C}^{3''}H$), 83.0 ($2x\underline{C}^{2'}H$), 101.5 ($2x\underline{C}^{1'}H$), 104.8 ($2x\underline{C}^{1''}H$), 170.6 ($2xCH_3\underline{C}{=}O$), 170.7 ($2xCH_3\underline{C}{=}O$).

Non-acetylated acidic glucolipid C18:0 ($C24H44O8$) (9a):

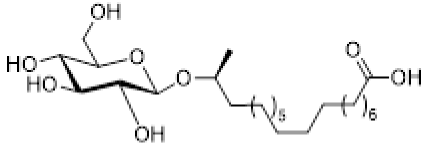

^{1}H NMR (400 MHz, MeOD): δ_H 1.12 (3H, d, J = 6.2 Hz, $C\underline{H}_3CH$), 1.15-1.27 (25H, m, $12xCH_2(CH_2)_2$, $C\underline{H}_aH_bCHCH_3$), 1.44-1.58 (3H, m, $\underline{C}H_2CH_2COOH$, $CH_a\underline{H}_bCHCH_3$), 2.17 (2H, t, J = 7.5 Hz, $C\underline{H}_2COOH$), 3.13-3.21 (1H, m, $C^{2'}H$), 3.23-3.41 (3H, m, $2xC\underline{H}OC$, $C^{3'}\underline{H}$), 3.68 (1H, dxd, J = 5.2, 5.4 Hz, $C\underline{H}_aH_bOH$), 3.67-3.72 (1H, m, $CH_3\underline{C}HCH_2$), 3.87 (1H, dxd, J = 2.2, 2.3 Hz, $CH_a\underline{H}_bOH$), 4.22 (1H, d, J = 7.7 Hz, $C^1\underline{H}$). **^{13}C NMR** (100 MHz, MeOD): δ_C 20.6 ($\underline{C}H_3CH$), 24.7 ($\underline{C}H_2CH_2COOH$), 25.0 ($\underline{C}H_2(CH_2)_2$), 28.8 ($\underline{C}H_2(CH_2)_2$), 29.0 ($\underline{C}H_2(CH_2)_2$), 29.2 ($\underline{C}H_2(CH_2)_2$), 29.4 ($7x\underline{C}H_2(CH_2)_2$), 29.5 ($\underline{C}H_2(CH_2)_2$), 33.6 ($\underline{C}H_2COOH$), 36.3 ($\underline{C}H_2CHCH_3$), 61.4 ($\underline{C}H_2OH$), 70.2 ($\underline{C}HOC$), 73.9 ($\underline{C}^{2'}H$), 76.2 ($CH_3\underline{C}HCH_2$), 76.3 ($\underline{C}HOC$), 76.7 ($\underline{C}^{3'}H$), 102.5 ($\underline{C}^{1'}H$), 176.4 ($\underline{C}OOH$).

Acetylated acidic glucolipid ω-1 C18:0 (9b):

Determined from a mixure (~50/50) of nonacetylayed and acetylated C18:0 ω-1 glucolipid together with some congeners. Only acetylated C18:0 ω sophorolipid acid is described. Acetic acid peaks are also present.

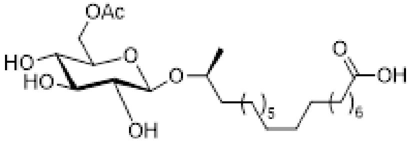

^{1}H NMR (400 MHz, MeOD): δ_H 1.23 (3H, d, *J* = 6.2 Hz, $C\underline{H}_3CH$), 1.26-1.40 (25H, m, 12x$CH_2(CH_2)_2$, $C\underline{H}_aH_bCHCH_3$), 1.39-1.47 (1H, m, $CH_a\underline{H}_bCHCH_3$), 1.56-1.67 (3H, m, $\underline{C}H_2CH_2COOH$, $CH_a\underline{H}_bCHCH_3$), 2.08 (3H, s, $C\underline{H}_3C{=}O$), 2.29 (2H, t, *J* = 7.5 Hz, $C\underline{H}_2(C{=}O)OH$), 3.18 (1H, m, $C^{2'}H$), 3.23-3.34 (2H, m, 2x$\underline{C}HOC$), 3.67-3.72 (1H, m, $CH_3\underline{C}HCH_2$), 3.72-3.79 (1H, m, $CH_3\underline{C}HCH_2$), 4.17-4.29 (1H, dxd, *J* = 11.7 Hz, 1.9 Hz, $CH_a\underline{H}_bOAc$), 4.38 (1H, dxd, *J* = 11.7 Hz, 1.9 Hz, $C\underline{H}_aH_bOAc$), 4.31 (1H, dxd, *J* = 8.0 Hz, 1.6 Hz, $C^{1'}\underline{H}$). **^{13}C NMR** (100 MHz, MeOD): δ_C 19.3 ($\underline{C}H_3OAc$), 20.6 ($\underline{C}H_3CH$), 24.7 ($\underline{C}H_2CH_2(C{=}O)OH$), 28.8 ($\underline{C}H_2(CH_2)_2$), 29.0 ($\underline{C}H_2(CH_2)_2$), 29.2 ($\underline{C}H_2(CH_2)_2$), 29.4 (8x$\underline{C}H_2(CH_2)_2$), 29.5 ($\underline{C}H_2(CH_2)_2$), 33.6 ($\underline{C}H_2(C{=}O)OH$), 36.4 ($\underline{C}H_2CHCH_3$), 63.5 ($\underline{C}H_2OH$), 70.3 ($\underline{C}HOC$), 73.6 ($\underline{C}^{2'}H$), 76.5 ($\underline{C}HOC$), 76.7 ($\underline{C}^{3'}H$), 76.8 ($CH_3\underline{C}HCH_2$), 102.7 ($\underline{C}^{1'}H$), 172.6 ($\underline{C}H_3C{=}O$), 172.7 ($\underline{C}H_3C{=}O$), 176.4 ($H\underline{C}{=}O$).

9-[(2'-*O*-β-D-glucopyranosyl-β-D-glucopyranosyl)oxy]nonaan-1-ol (11a):

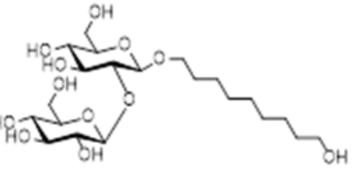

^{1}H NMR (400 MHz, DMSO-d$_6$): δ_H 1.21-1.33 (10H, m, 5x$C\underline{H}_2(CH_2)_2$), 1.36-1.44 (2H, m, $C\underline{H}_2CH_2OH$), 1.45-1.55 (2H, m, $CHOCH_2C\underline{H}_2$), 2.99 (1H, dxd, *J* = 8.3, 8.3 Hz, $C^{2''}\underline{H}$), 3.02-3.17 (5H, m, 4x$C\underline{H}OC$, $C^{3''}\underline{H}$), 3.21 (1H, dxd, *J* = 8.7, 7.9 Hz, $C^{2'}\underline{H}$), 3.30-3.40 (3H, m, $C^{3'}\underline{H}$, $CH_2C\underline{H}_2OH$), 3.43 (1H, dxt, *J* = 9.0, 6.8 Hz, $CHOC\underline{H}_aH_bCH_2$), 3.40-3.54 (2H, m, 2x$CHC\underline{H}_aH_bOH$), 3.59-3.69 (2H, m, 2x$CHCH_a\underline{H}_bOH$), 3.76 (1H, dxt, *J* = 8.9, 6.8 Hz, $CHOCH_a\underline{H}_bCH_2$), 4.27 (1H, d, *J* = 7.7 Hz, $C^{1'}\underline{H}$), 4.37 (1H, d, *J* = 7.8 Hz, $C^{1''}\underline{H}$), 4.29-4.36 (2H, m, $CHCH_2O\underline{H}$, $CH_2CH_2O\underline{H}$), 4.51 (1H, br s, $CHCH_2O\underline{H}$), 4.89 (1H, br s, $O\underline{H}$), 4.95 (1H, br s, $O\underline{H}$), 5.07 (1H, br s, $O\underline{H}$), 5.19 (1H, br s, $O\underline{H}$), 5.54 (1H, br s, $O\underline{H}$). **^{13}C NMR** (100 MHz, DMSO-d$_6$): δ_C 26.0 (2x$\underline{C}H_2(CH_2)_2$), 29.4 (2x$\underline{C}H_2(CH_2)_2$), 29.5 ($\underline{C}H_2(CH_2)_2$), 29.7 ($CHOCH_2\underline{C}H_2$), 33.0 ($\underline{C}H_2CH_2OH$), 61.2 ($CH_2\underline{C}H_2OH$), 61.3 ($CH\underline{C}H_2OH$), 61.3 ($CH\underline{C}H_2OH$), 69.1.

9-[(6',6''-di-*O*-acetyl-2'-*O*-β-D-glucopyranosyl-β-D-glucopyranosyl)oxy]nonaan-1-ol (11b):

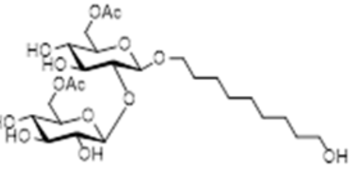

^{1}H NMR (400 MHz, DMSO-d$_6$): δ_H 1.17-1.33 (10H, m, 5x$C\underline{H}_2(CH_2)_2$), 1.35-1.44 (2H, m, $C\underline{H}_2CH_2OH$), 1.44-1.53 (2H, m, $CHOCH_2C\underline{H}_2$), 2.00 (3H, s, $C\underline{H}_3C{=}O$), 2.02 (3H, s, $C\underline{H}_3C{=}O$), 3.01 (1H, dxd, *J* = 8.3, 8.3 Hz, $C^{2''}\underline{H}$), 3.05-3.19 (3H, m, 2x$C\underline{H}OC$, $C^{3''}\underline{H}$), 3.22 (1H, dxd, *J* = 8.7, 8.0 Hz, $C^{2'}\underline{H}$), 3.27-3.42 (5H, m, 2x$C\underline{H}CH_2OAc$, $C^{3'}\underline{H}$, $C\underline{H}_2OH$), 3.45 (1H, dxt, *J* = 9.3, 6.8 Hz, $CHOC\underline{H}_aH_bCH_2$), 3.67 (1H, dxt, *J* = 9.4, 6.6 Hz, $CHOCH_a\underline{H}_bCH_2$), 4.03 (1H, dxd, *J* = 11.6, 5.6 Hz, $C\underline{H}_aH_bOAc$), 4.04 (1H, dxd, *J* = 11.7, 6.5 Hz, $C\underline{H}_aH_bOAc$), 4.22 (1H, dxd, *J* = 12.1, 1.9 Hz, $CH_a\underline{H}_bOAc$), 4.25 (1H, dxd, *J* = 12.1, 1.5 Hz, $CH_a\underline{H}_bOAc$), 4.32 (1H, d, *J* = 7.7 Hz, $C^{1'}\underline{H}$), 4.41 (1H, d, *J* = 8.0 Hz, $C^{1''}\underline{H}$), 4.91-5.67 (5H, m, 5x$O\underline{H}$). **^{13}C NMR** (100 MHz, DMSO-d$_6$): δ_C 20.7 (2x$\underline{C}H_3C{=}O$), 25.4 ($\underline{C}H_2(CH_2)_2$), 25.5 ($\underline{C}H_2(CH_2)_2$), 28.9 ($\underline{C}H_2(CH_2)_2$), 29.0 ($\underline{C}H_2(CH_2)_2$), 29.1 ($\underline{C}H_2(CH_2)_2$), 29.3 ($CHOCH_2\underline{C}H_2$), 32.6 ($\underline{C}H_2CH_2OH$), 60.8 ($\underline{C}H_2OH$), 63.5 ($\underline{C}H_2OAc$), 63.8 ($\underline{C}H_2OAc$), 68.6 ($CHO\underline{C}H_2CH_2$), 69.6 ($\underline{C}HOC$), 69.8 ($\underline{C}HOC$), 73.3 ($\underline{C}HOC$), 73.9 ($\underline{C}HOC$), 74.8 ($\underline{C}^{2''}H$), 75.8 ($\underline{C}^{3'}H$), 75.9 ($\underline{C}^{3''}H$), 82.6 ($\underline{C}^{2'}H$), 101.1 ($\underline{C}^{1'}H$), 104.3 ($\underline{C}^{1''}H$), 170.29 ($CH_3\underline{C}{=}O$), 170.31 ($CH_3\underline{C}{=}O$). **IR** (ATR, cm^{-1}): ν_{max} = 1024, 1048, 1080, 1240 (COAc), 1703 (C=O), 1746 (C=O), 3410 (OH).

General procedure for synthesis of sophoroside amines (12)

***N*-ethyl-*N*-methyl-9-[(2'-*O*-β-D-glucopyranosyl-β-D-glucopyranosyl)oxy]nonaan-1-amine (12a):**

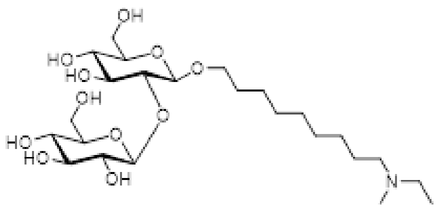

^{1}H NMR (400 MHz, DMSO-d$_6$): δ_H 0.95 (3H, t, *J* = 7.1 Hz, $CH_2C\underline{H}_3$), 1.16-1.33 (10H, m, 5x$C\underline{H}_2(CH_2)_2$), 1.31-1.44 (2H, m, $C\underline{H}_2CH_2N$), 1.45-1.58 (2H, m, $CHOCH_2C\underline{H}_2$), 2.08 (3H, s, $C\underline{H}_3N$), 2.23 (2H, txd, *J* = 7.4, 2.5 Hz, $CH_2C\underline{H}_2N$), 2.30 (2H, dxd, *J* = 7.1, 7.1 Hz $CH_3C\underline{H}_2N$), 2.99 (1H, dxd, *J* = 8.2, 8.2 Hz, $C^{2''}\underline{H}$), 3.02-3.08 (1H, m, $C\underline{H}OC$), 3.08-3.12 (2H, m, 2x$C\underline{H}OC$), 3.13-3.17 (2H, m, $C\underline{H}OC$, $C^{3''}H$), 3.20 (1H, dxd, *J* = 9.2, 8.7 Hz, $C^{2'}H$), 3.28-3.39 (1H, m, $C^{3'}\underline{H}$), 3.39-3.47 (3H, m, $CHOC\underline{H}_aH_bCH_2$, 2x$C\underline{H}_aH_bOH$), 3.57-3.70 (2H, m, 2x$CH_a\underline{H}_bOH$), 3.71-3.80 (1H, m, $CHOCH_a\underline{H}_bCH_2$), 4.26 (1H, d, *J* = 7.8 Hz, $C^{1'}\underline{H}$), 4.37 (1H, d, *J* = 7.8 Hz, $C^{1''}\underline{H}$), 4.52 (2H, br s, $CH_2O\underline{H}$), 4.86-5.07 (2H, m, 2x$O\underline{H}$), 5.13 (1H, br s, $O\underline{H}$), 5.23 (1H, br s, $O\underline{H}$), 5.54 (1H, br s, $O\underline{H}$). **^{13}C NMR** (100 MHz, DMSO-d$_6$): δ_C 12.7 ($CH_2\underline{C}H_3$), 25.9 ($\underline{C}H_2(CH_2)_2$), 27.2 ($\underline{C}H_2CH_2N$), 27.4 ($\underline{C}H_2(CH_2)_2$), 29.4 (2x$\underline{C}H_2(CH_2)_2$), 29.5 ($\underline{C}H_2(CH_2)_2$), 29.7 ($CHOCH_2\underline{C}H_2$), 41.7 ($\underline{C}H_3N$), 51.3 ($CH_3\underline{C}H_2N$), 57.1 ($CH_2\underline{C}H_2N$), 61.3 ($\underline{C}H_2OH$), 61.3 ($\underline{C}H_2OH$), 69.1 ($CHO\underline{C}H_2CH_2$), 70.2 ($\underline{C}HOC$), 70.2 ($\underline{C}HOC$), 75.3 ($\underline{C}^{2''}H$), 76.4 ($\underline{C}^{3'}H$), 76.5 ($\underline{C}^{3''}H$), 77.0 ($\underline{C}HOC$), 77.4 ($\underline{C}HOC$), 82.7 ($\underline{C}^{2'}H$), 101.7 ($\underline{C}^{1'}H$), 104.6 ($\underline{C}^{1''}H$). **HRMS (ESI):** *m/z* exact mass calculated for $C_{24}H_{47}NO_{11}$ [M+H$^+$]: 526.3149; found: 526.3221.

***N*-butyl-*N*-methyl-9-[(2'-*O*-β-D-glucopyranosyl-β-D-glucopyranosyl)oxy]nonaan-1-amine (13a):**

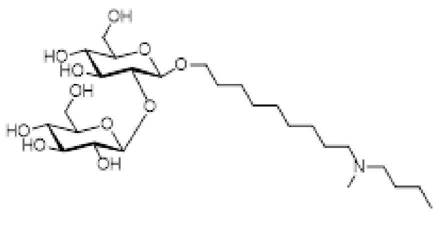

^{1}H NMR (400 MHz, DMSO-d$_6$): δ_H 0.87 (3H, t, *J* = 7.2 Hz, $CH_2C\underline{H}_3$), 1.14-1.32 (12H, m, 6x$C\underline{H}_2(CH_2)_2$, $C\underline{H}_2CH_3$), 1.31-1.44 (4H, m, 2x$C\underline{H}_2CH_2N$), 1.45-1.58 (2H, m, $CHOCH_2C\underline{H}_2$), 2.08 (3H, s, $C\underline{H}_3N$), 2.23 (4H, txd, *J* = 7.4, 2.5 Hz, 2x$CH_2C\underline{H}_2N$), 2.99 (1H, dxd, *J* = 8.2, 8.2 Hz, $C^{2''}\underline{H}$), 3.02-3.08 (1H, m, $C\underline{H}OC$), 3.08-3.12 (2H, m, 2x$C\underline{H}OC$), 3.13-3.17 (2H, m, $C\underline{H}OC$, $C^{3''}H$), 3.20 (1H, dxd, *J* = 9.2, 8.7 Hz, $C^{2'}H$), 3.32-3.39 (1H, m, $C^{3'}\underline{H}$), 3.39-3.47 (2H, m, $CHOC\underline{H}_aH_bCH_2$, $C\underline{H}_aH_bOH$), 3.49 (1H, dxd, *J* = 4.5, 4.6 Hz, $C\underline{H}_aH_bOH$), 3.57-3.70 (2H, m, 2x$CH_a\underline{H}_bOH$), 3.71-3.80 (1H, m, $CHOCH_a\underline{H}_bCH_2$), 4.26 (1H, d, *J* = 7.7 Hz, $C^{1'}\underline{H}$), 4.37 (1H, d, *J* = 7.7 Hz, $C^{1''}\underline{H}$), 4.51 (2H, br s, $CH_2O\underline{H}$), 4.77-5.37 (4H, m, 4x$O\underline{H}$), 5.50 (1H, br s, $O\underline{H}$). **^{13}C NMR** (100 MHz, DMSO-d$_6$): δ_C 14.3 ($CH_2\underline{C}H_3$), 20.5 ($\underline{C}H_2CH_3$), 25.9 ($\underline{C}H_2(CH_2)_2$), 27.2 ($\underline{C}H_2CH_2N$), 27.3 ($\underline{C}H_2CH_2N$), 29.4 (3x$\underline{C}H_2(CH_2)_2$), 29.5 ($\underline{C}H_2(CH_2)_2$), 29.7 ($CHOCH_2\underline{C}H_2$), 42.3 ($\underline{C}H_3N$), 57.3 ($CH_2\underline{C}H_2N$), 57.6 ($CH_2\underline{C}H_2N$), 61.3 ($\underline{C}H_2OH$), 61.3 ($\underline{C}H_2OH$), 69.1 ($CHO\underline{C}H_2CH_2$), 70.2 ($\underline{C}HOC$), 70.2 ($\underline{C}HOC$), 75.3 ($\underline{C}^{2''}H$), 76.4 ($\underline{C}^{3'}H$), 76.5 ($\underline{C}^{3''}H$), 77.0 ($\underline{C}HOC$), 77.4 ($\underline{C}HOC$), 82.7 ($\underline{C}^{2'}H$), 101.7 ($\underline{C}^{1'}H$), 104.6 ($\underline{C}^{1''}H$). **HRMS (ESI):** *m/z* exact mass calculated for $C_{26}H_{51}NO_{11}$ [M+H$^+$]: 554.3462; found: 554.3532.

***N*-hexyl-*N*-methyl-9-[(2'-*O*-β-D-glucopyranosyl-β-D-glucopyranosyl)oxy]nonaan-1-amine (14a):**

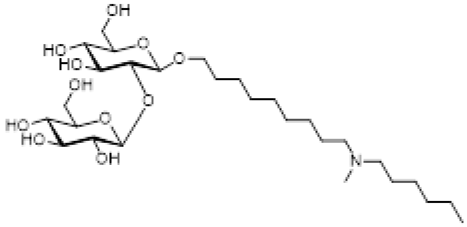

^{1}H NMR (400 MHz, DMSO-d$_6$): δ_H 0.86 (3H, t, *J* = 6.9 Hz, $CH_2C\underline{H}_3$), 1.14-1.32 (16H, m, 7x$C\underline{H}_2(CH_2)_2$, $C\underline{H}_2CH_3$), 1.32-1.42 (4H, m, 2x$C\underline{H}_2CH_2N$), 1.45-1.57 (2H, m, $CHOCH_2C\underline{H}_2$), 2.08 (3H, s, $C\underline{H}_3N$), 2.21 (4H, t, *J* = 7.2 Hz, 2x$CH_2C\underline{H}_2N$), 2.99 (1H, dxd, *J* = 8.2, 8.2 Hz, $C^{2''}\underline{H}$), 3.02-3.08 (1H, m, $C\underline{H}OC$), 3.08-3.12 (2H, m, 2x$C\underline{H}OC$), 3.13-3.17 (2H, m, $C\underline{H}OC$, $C^{3''}H$), 3.21 (1H, dxd, *J* = 9.2, 8.7 Hz, $C^{2'}H$), 3.36-3.40 (1H, m, $C^{3'}\underline{H}$), 3.39-3.47 (2H, m, $CHOC\underline{H}_aH_bCH_2$, $C\underline{H}_aH_bOH$), 3.50 (1H, dxd, *J* = 11.9, 4.3 Hz, $C\underline{H}_aH_bOH$), 3.57-3.70 (2H, m, 2x$CH_a\underline{H}_bOH$), 3.71-3.80 (1H, m, $CHOCH_a\underline{H}_bCH_2$), 4.26 (1H, d, *J* = 7.6 Hz, $C^{1'}\underline{H}$), 4.37 (1H, d, *J* = 7.8 Hz, $C^{1''}\underline{H}$), 4.52 (2H, br s, $CH_2O\underline{H}$), 4.84-5.05 (2H, m, 2x$O\underline{H}$), 5.10 (1H, br s, $O\underline{H}$) 5.20 (1H, br s, $O\underline{H}$), 5.49 (1H, br s, $O\underline{H}$). **^{13}C NMR** (100 MHz, DMSO-d$_6$): δ_C 14.4 ($CH_2\underline{C}H_3$), 22.5 ($\underline{C}H_2CH_3$), 25.9 ($\underline{C}H_2(CH_2)_2$), 27.0 ($\underline{C}H_2CH_2N$), 27.2 ($\underline{C}H_2CH_2N$), 27.2 ($\underline{C}H_2(CH_2)_2$), 27.3 ($\underline{C}H_2(CH_2)_2$), 29.4 ($\underline{C}H_2(CH_2)_2$), 29.5 (2x$\underline{C}H_2(CH_2)_2$), 29.7 ($CHOCH_2\underline{C}H_2$), 31.7 ($\underline{C}H_2(CH_2)_2$), 42.4 ($\underline{C}H_3N$), 57.6 (2x$CH_2\underline{C}H_2N$), 61.3 ($\underline{C}H_2OH$), 61.3

($\underline{C}H_2OH$), 69.1 ($CHO\underline{C}H_2CH_2$), 70.2 ($\underline{C}HOC$), 70.3 ($\underline{C}HOC$), 75.3 ($\underline{C}^{2''}H$), 76.4 ($\underline{C}^{3'}H$), 76.5 ($\underline{C}^{3''}H$), 77.0 ($\underline{C}HOC$), 77.4 ($\underline{C}HOC$), 82.7 ($\underline{C}^{2'}H$), 101.7 ($\underline{C}^{1'}H$), 104.5 ($\underline{C}^{1''}H$). **HRMS (ESI):** *m/z* exact mass calculated for $C_{28}H_{55}NO_{11}$ [$M+H^+$]: 582.3847; found: 582.3775.

***N*-methyl-*N*-ocytl-9-[(2'-*O*-β-D-glucopyranosyl-β-D-glucopyranosyl)oxy]nonaan-1-amine (15a):**

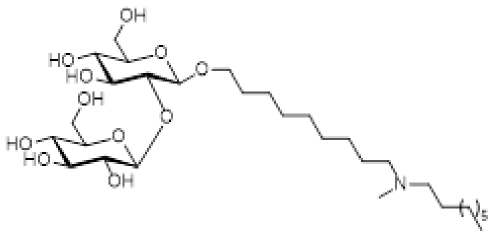

^{1}H NMR (400 MHz, DMSO-d$_6$): δ_H 0.86 (3H, t, *J* = 7.0 Hz, $CH_2C\underline{H}_3$), 1.15-1.32 (20H, m, $9xC\underline{H}_2(CH_2)_2$, $C\underline{H}_2CH_3$), 1.33-1.43 (4H, m, $2xC\underline{H}_2CH_2N$), 1.44-1.57 (2H, m, $CHOCH_2C\underline{H}_2$), 2.07 (3H, s, $C\underline{H}_3N$), 2.21 (4H, t, *J* = 7.2 Hz, $2xCH_2C\underline{H}_2N$), 2.99 (1H, dxd, *J* = 8.2, 8.2 Hz, $C^{2''}\underline{H}$), 3.02-3.08 (1H, m, $C\underline{H}OC$), 3.08-3.12 (2H, m, $2xC\underline{H}OC$), 3.13-3.17 (2H, m, $C\underline{H}OC$, $C^{3''}H$), 3.21 (1H, dxd, *J* = 9.2, 8.7 Hz, $C^{2'}H$), 3.36-3.40 (1H, m, $C^{3'}\underline{H}$), 3.40-3.47 (2H, m, $CHOC\underline{H}_aH_bCH_2$, $C\underline{H}_aH_bOH$), 3.50 (1H, dxd, *J* = 11.9, 4.3 Hz, $C\underline{H}_aH_bOH$), 3.57-3.70 (2H, m, $2xCH_a\underline{H}_bOH$), 3.71-3.80 (1H, m, $CHOCH_a\underline{H}_bCH_2$), 4.26 (1H, d, *J* = 7.9 Hz, $C^{1'}\underline{H}$), 4.37 (1H, d, *J* = 7.4 Hz, $C^{1''}\underline{H}$), 4.52 (2H, br s, $CH_2O\underline{H}$), 4.84-5.05 (2H, m, $2xO\underline{H}$), 5.10 (1H, br s, $O\underline{H}$) 5.20 (1H, br s, $O\underline{H}$), 5.49 (1H, br s, $O\underline{H}$). **^{13}C NMR** (100 MHz, DMSO-d$_6$): δ_C 14.4 ($CH_2\underline{C}H_3$), 22.5 ($\underline{C}H_2CH_3$), 25.9 ($\underline{C}H_2(CH_2)_2$), 27.1 ($\underline{C}H_2CH_2N$), 27.2 ($\underline{C}H_2CH_2N$), 27.3 ($\underline{C}H_2(CH_2)_2$), 27.4 ($\underline{C}H_2(CH_2)_2$), 29.1 ($\underline{C}H_2(CH_2)_2$), 29.3-29.6 ($4x\underline{C}H_2(CH_2)_2$), 29.7 ($CHOCH_2\underline{C}H_2$), 31.7 ($\underline{C}H_2(CH_2)_2$), 42.3 ($\underline{C}H_3N$), 57.6 ($CH_2\underline{C}H_2N$), 57.6 ($CH_2\underline{C}H_2N$), 61.3 ($\underline{C}H_2OH$), 61.4 ($\underline{C}H_2OH$), 69.1 ($CHO\underline{C}H_2CH_2$), 70.2 ($\underline{C}HOC$), 70.3 ($\underline{C}HOC$), 75.3 ($\underline{C}^{2''}H$), 76.4 ($\underline{C}^{3'}H$), 76.5 ($\underline{C}^{3''}H$), 77.0 ($\underline{C}HOC$), 77.4 ($\underline{C}HOC$), 82.7 ($\underline{C}^{2'}H$), 101.7 ($\underline{C}^{1'}H$), 104.6 ($\underline{C}^{1''}H$). **HRMS (ESI):** *m/z* exact mass calculated for $C_{30}H_{59}NO_{11}$ [$M+H^+$]: 610.4162; found: 610.4088.

***N*-dodecyl-*N*-ocytl-9-[(2'-*O*-β-D-glucopyranosyl-β-D-glucopyranosyl)oxy]nonaan-1-amine (16a):**

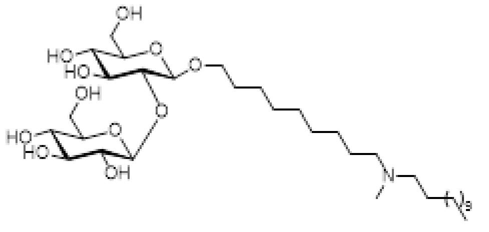

^{1}H NMR (400 MHz, DMSO-d$_6$): δ_H 0.86 (3H, t, *J* = 7.0 Hz, $CH_2C\underline{H}_3$), 1.15-1.33 (28H, m, $13xC\underline{H}_2(CH_2)_2$, $C\underline{H}_2CH_3$), 1.33-1.43 (4H, m, $2xC\underline{H}_2CH_2N$), 1.44-1.57 (2H, m, $CHOCH_2C\underline{H}_2$), 2.09 (3H, s, $C\underline{H}_3N$), 2.21 (4H, t, *J* = 7.2 Hz, $2xCH_2C\underline{H}_2N$), 2.99 (1H, dxd, *J* = 8.2, 8.2 Hz, $C^{2''}\underline{H}$), 3.02-3.08 (1H, m, $C\underline{H}OC$), 3.08-3.12 (2H, m, $2xC\underline{H}OC$), 3.13-3.17 (2H, m, $C\underline{H}OC$, $C^{3''}H$), 3.20 (1H, dxd, *J* = 9.2, 8.7 Hz, $C^{2'}H$), 3.36-3.40 (1H, m, $C^{3'}\underline{H}$), 3.40-3.47 (2H, m, $CHOC\underline{H}_aH_bCH_2$, $C\underline{H}_aH_bOH$), 3.50 (1H, dxd, *J* = 11.9, 4.3 Hz, $C\underline{H}_aH_bOH$), 3.57-3.70 (2H, m, $2xCH_a\underline{H}_bOH$), 3.71-3.80 (1H, m, $CHOCH_a\underline{H}_bCH_2$), 4.26 (1H, d, *J* = 7.6 Hz, $C^{1'}\underline{H}$), 4.37 (1H, d, *J* = 7.9 Hz, $C^{1''}\underline{H}$), 4.51 (2H, br s, $CH_2O\underline{H}$), 4.82-5.03 (2H, m, $2xO\underline{H}$), 5.11 (1H, br s, $O\underline{H}$) 5.21 (1H, br s, $O\underline{H}$), 5.50 (1H, br s, $O\underline{H}$). **^{13}C NMR** (100 MHz, DMSO-d$_6$): δ_C 14.4 ($CH_2\underline{C}H_3$), 22.5 ($\underline{C}H_2CH_3$), 25.9 ($\underline{C}H_2(CH_2)_2$), 27.1 ($\underline{C}H_2CH_2N$), 27.2 ($\underline{C}H_2CH_2N$), 27.3 ($\underline{C}H_2(CH_2)_2$), 27.4 ($\underline{C}H_2(CH_2)_2$), 29.1 ($\underline{C}H_2(CH_2)_2$), 29.3-29.6 ($8x\underline{C}H_2(CH_2)_2$), 29.7 ($CHOCH_2\underline{C}H_2$), 31.7 ($\underline{C}H_2(CH_2)_2$), 42.3 ($\underline{C}H_3N$), 57.5 ($CH_2\underline{C}H_2N$), 57.6 ($CH_2\underline{C}H_2N$), 61.3 ($\underline{C}H_2OH$), 61.4 ($\underline{C}H_2OH$), 69.1 ($CHO\underline{C}H_2CH_2$), 70.2 ($\underline{C}HOC$), 70.3 ($\underline{C}HOC$), 75.3 ($\underline{C}^{2''}H$), 76.4 ($\underline{C}^{3'}H$), 76.5 ($\underline{C}^{3''}H$), 77.0 ($\underline{C}HOC$), 77.4 ($\underline{C}HOC$), 82.8 ($\underline{C}^{2'}H$), 101.8 ($\underline{C}^{1'}H$), 104.6 ($\underline{C}^{1''}H$). **HRMS (ESI):** *m/z* exact mass calculated for $C_{34}H_{67}NO_{11}$ [$M+H^+$]: 666.4785; found: 666.4714.

***N*-methyl-*N*-octadecyl-9-[(2'-*O*-β-D-glucopyranosyl-β-D-glucopyranosyl)oxy]nonanal-1-amine (17a):**

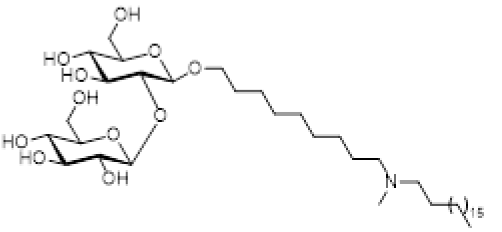

^{1}H NMR (400 MHz, DMSO-d$_6$): δ_H 0.86 (3H, t, *J* = 6.8 Hz, $CH_2C\underline{H}_3$), 1.12-1.33 (40H, m, 19x$C\underline{H}_2(CH_2)_2$, $C\underline{H}_2CH_3$), 1.33-1.43 (4H, m, 2x$C\underline{H}_2CH_2N$), 1.43-1.57 (2H, m, $CHOCH_2C\underline{H}_2$), 2.09 (3H, s, $C\underline{H}_3N$), 2.21 (4H, t, *J* = 7.3 Hz, 2x$CH_2C\underline{H}_2N$), 2.99 (1H, dxd, *J* = 8.2, 8.2 Hz, $C^{2''}\underline{H}$), 3.02-3.08 (1H, m, $C\underline{H}OC$), 3.08-3.12 (2H, m, 2x$C\underline{H}OC$), 3.13-3.17 (2H, m, $C\underline{H}OC$, $C^{3''}H$), 3.21 (1H, dxd, *J* = 9.2, 8.7 Hz, $C^{2'}H$), 3.36-3.40 (1H, m, $C^{3'}\underline{H}$), 3.40-3.47 (2H, m, $CHOC\underline{H_a}H_bCH_2$, $C\underline{H}_aH_bOH$), 3.50 (1H, dxd, *J* = 11.9, 4.3 Hz, $C\underline{H}_aH_bOH$), 3.57-3.70 (2H, m, 2x$CH_a\underline{H_b}OH$), 3.71-3.80 (1H, m, $CHOCH_a\underline{H_b}CH_2$), 4.26 (1H, d, *J* = 7.6 Hz, $C^{1'}\underline{H}$), 4.29-4.35 (1H, br s, $O\underline{H}$), 4.37 (1H, d, *J* = 7.9 Hz, $C^{1''}\underline{H}$), 4.46-4.56 (2H, m, 2x$CH_2O\underline{H}$), 4.93 (2H, m, 2x$O\underline{H}$), 5.08 (1H, br s, $O\underline{H}$) 5.19 (1H, br s, $O\underline{H}$), 5.48 (1H, br s, $O\underline{H}$). **^{13}C NMR** (100 MHz, DMSO-d$_6$): δ_C 14.4 ($CH_2\underline{C}H_3$), 22.5 ($\underline{C}H_2CH_3$), 26.0 ($\underline{C}H_2(CH_2)_2$), 27.1 ($\underline{C}H_2CH_2N$), 27.2 ($\underline{C}H_2CH_2N$), 27.3 ($\underline{C}H_2(CH_2)_2$), 27.4 ($\underline{C}H_2(CH_2)_2$), 29.1 ($\underline{C}H_2(CH_2)_2$), 29.3-29.6 (14x$\underline{C}H_2(CH_2)_2$), 29.7 ($CHOCH_2\underline{C}H_2$), 31.7 ($\underline{C}H_2(CH_2)_2$), 42.3 ($\underline{C}H_3N$), 57.6 ($CH_2\underline{C}H_2N$), 57.6 ($CH_2\underline{C}H_2N$), 61.3 ($\underline{C}H_2OH$), 61.4 ($\underline{C}H_2OH$), 69.1 ($CHO\underline{C}H_2CH_2$), 70.2 ($\underline{C}HOC$), 70.3 ($\underline{C}HOC$), 75.3 ($\underline{C}^{2''}H$), 76.4 ($\underline{C}^{3'}H$), 76.5 ($\underline{C}^{3''}H$), 77.0 ($\underline{C}HOC$), 77.4 ($\underline{C}HOC$), 82.8 ($\underline{C}^{2'}H$), 101.8 ($\underline{C}^{1'}H$), 104.6 ($\underline{C}^{1''}H$). **HRMS (ESI):** *m/z* exact mass calculated for $C_{40}H_{79}NO_{11}$ [M+H$^+$]: 750.5722; found: 750.5653.

***N*,*N*-dimethyl-*N*-ethyl-9-[(2'-*O*-β-D-glucopyranosyl-β-D-glucopyranosyl)oxy]nonaan-1-ammonium iodide (18a):**

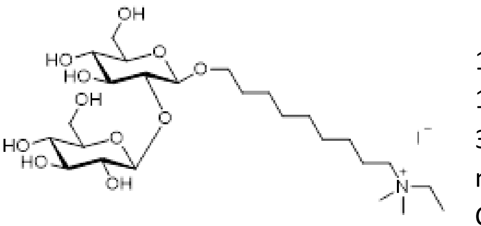

^{1}H NMR (400 MHz, DMSO-d$_6$): δ_H 1.20-1.25 (3H, m, $CH_2C\underline{H}_3$), 1.24-1.38 (10H, m, 5x$C\underline{H}_2(CH_2)_2$), 1.44-1.56 (2H, m, $CHOCH_2C\underline{H}_2$), 1.55-1.70 (2H, m, $C\underline{H}_2CH_2N$), 2.96-3.04 (1H, m, $C^{2''}\underline{H}$), 2.99 (6H, s, $C\underline{H}_3N$), 3.04-3.08 (1H, m, $C\underline{H}OC$), 3.08-3.13 (1H, m, $C\underline{H}OC$), 3.12-3.17 (3H, m, $C\underline{H}OC$, $C^{3''}\underline{H}$), 3.17-3.25 (3H, m, $C^{2'}\underline{H}$, $C\underline{H}_2N$), 3.28-3.35 (2H, m, $C\underline{H}_2N$), 3.33-3.39 (1H, m, $C^{3'}\underline{H}$), 3.41-3.53 (3H, m, 2x$C\underline{H}_aH_bOH$, $CHOC\underline{H}_aH_bCH_2$), 3.58-3.70 (2H, m, 2x$CH_a\underline{H_b}OH$), 3.71-3.82 (1H, m, $CHOCH_a\underline{H_b}CH_2$), 4.27 (1H, d, *J* = 7.7 Hz, $C^{1'}\underline{H}$), 4.30-4.35 (1H, m, $O\underline{H}$), 4.37 (1H, dxd, *J* = 8.3, 4.0 Hz, $C^{1''}\underline{H}$), 4.50 (1H, t, *J* = 5.7, $O\underline{H}$), 4.81-4.99 (2H, m, $O\underline{H}$), 5.03-5.10 (1H, m, $O\underline{H}$) 5.17 (1H, br s, $O\underline{H}$), 5.44 (1H, dxd, , *J* = 9.3, 3.3 $O\underline{H}$). **^{13}C NMR** (100 MHz, DMSO-d$_6$): δ_C 8.2 ($CH_2\underline{C}H_3$), 22.1 ($\underline{C}H_2CH_2N$), 25.9 ($\underline{C}H_2(CH_2)_2$), 26.3 ($\underline{C}H_2(CH_2)_2$), 28.9 ($\underline{C}H_2(CH_2)_2$), 29.2 ($\underline{C}H_2(CH_2)_2$), 29.3 ($\underline{C}H_2(CH_2)_2$), 29.7 ($CHOCH_2\underline{C}H_2$), 49.8 (2x$\underline{C}H_3N$), 58.9 ($N\underline{C}H_2CH_3$), 61.3 (2x$\underline{C}H_2OH$), 62.9 ($CH_2\underline{C}H_2N$), 69.1 ($CHO\underline{C}H_2CH_2$), 70.2 ($\underline{C}HOC$), 70.3 ($\underline{C}HOC$), 75.3 ($\underline{C}^{2''}H$), 76.4 ($\underline{C}^{3'}H$), 76.5 ($\underline{C}^{3''}H$), 77.0 ($\underline{C}HOC$), 77.4 ($\underline{C}HOC$), 82.7 ($\underline{C}^{2'}H$), 101.7 ($\underline{C}^{1'}H$), 104.5 ($\underline{C}^{1''}H$). **HRMS (ESI):** *m/z* exact mass calculated for $C_{25}H_{50}NO_{11}$ [M-I$^-$]: 540.3378; found: 540.3365.

***N*-butyl-*N*,*N*-dimethyl-9-[(2'-*O*-β-D-glucopyranosyl-β-D-glucopyranosyl)oxy]nonaan-1-ammonium iodide (19a):**

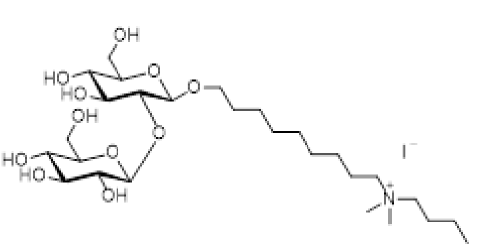

^{1}H NMR (400 MHz, DMSO-d$_6$): δ_H 0.94 (3H, t, *J* = 6.2 Hz, $CH_2C\underline{H}_3$), 1.13-1.40 (12H, m, 5x$C\underline{H}_2(CH_2)_2$, $C\underline{H}_2CH_3$), 1.42-1.57 (2H, m, $CHOCH_2C\underline{H}_2$), 1.56-1.75 (4H, m, 2x$C\underline{H}_2CH_2N$), 2.97-3.04 (1H, m, $C^{2''}\underline{H}$), 2.98 (6H, s, 2x$C\underline{H}_3N$), 3.02-3.08 (1H, m, $C\underline{H}OC$), 3.08-3.12 (2H, m, 2x$C\underline{H}OC$), 3.12-3.17 (2H, m, $C\underline{H}OC$, $C^{3''}\underline{H}$), 3.17-3.28 (5H, m, $C^{2'}\underline{H}$, 2x$C\underline{H}_2N$), 3.39-3.40 (1H, m, $C^{3'}\underline{H}$), 3.40-3.54 (3H, m, $C\underline{H}_aH_bOH$, $CHOC\underline{H}_aH_bCH_2$), 3.57-3.71 (2H, m, 2x$CH_a\underline{H_b}OH$), 3.71-3.82 (1H, m, $CHOCH_a\underline{H_b}CH_2$), 4.26 (1H, d, *J* = 7.4 Hz, $C^{1'}\underline{H}$), 4.30-4.36 (1H, m, $O\underline{H}$), 4.38 (1H, dxd, *J* = 7.9 Hz, $C^{1''}\underline{H}$), 4.50 (1H, t, *J* = 5.8, $O\underline{H}$), 4.83-4.99 (2H, m, $O\underline{H}$), 5.00-5.08 (1H, m, $O\underline{H}$) 5.17 (1H, d, *J* = 2.7, $O\underline{H}$), 5.44 (1H, d, *J* = 3.1, $O\underline{H}$). **^{13}C NMR** (100 MHz,

DMSO-d$_6$): δ_C 13.9 (CH$_2$CH$_3$), 19.7 (CH$_2$CH$_3$), 22.1 (CH$_2$CH$_2$N), 24.2 (CH$_2$CH$_2$N), 25.9 (CH$_2$(CH$_2$)$_2$), 26.2 (CH$_2$(CH$_2$)$_2$), 28.9 (CH$_2$(CH$_2$)$_2$), 29.2 (CH$_2$(CH$_2$)$_2$), 29.3 (CH$_2$(CH$_2$)$_2$), 29.7 (CHOCH$_2$CH$_2$), 50.4 (2xCH$_3$N), 61.3 (2xCH$_2$OH), 63.3 (CH$_2$CH$_2$N), 63.3 (CH$_2$CH$_2$N), 69.1 (CHOCH$_2$CH$_2$), 70.2 (CHOC), 70.3 (CHOC), 75.3 (C$^{2''}$H), 76.4 (C$^{3'}$H), 76.5 (C$^{3''}$H), 77.0 (CHOC), 77.4 (CHOC), 82.7 (C$^{2'}$H), 101.7 (C$^{1'}$H), 104.5 (C$^{1''}$H). **HRMS (ESI):** *m/z* exact mass calculated for $C_{27}H_{54}NO_{11}$ [M-I$^-$]: 568.3691; found: 568.3689.

***N*,*N*-dimethyl-*N*-hexyl-9-[(2’-*O*-β-D-glucopyranosyl-β-D-glucopyranosyl)oxy]nonaan-1-ammonium iodide (20a): (2.08 ppm acetonitrile is present)**

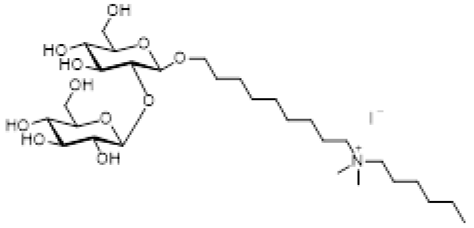

^{1}H NMR (400 MHz, DMSO-d$_6$): δ_H 0.88 (3H, t, *J* = 6.2 Hz, CH$_2$CH$_3$), 1.12-1.44 (16H, m, 7xCH$_2$(CH$_2$)$_2$, CH$_2$CH$_3$), 1.42-1.57 (2H, m, CHOCH$_2$CH$_2$), 1.56-1.75 (4H, m, 2xCH$_2$CH$_2$N), 2.97-3.04 (1H, m, C$^{2''}$H), 3.00 (6H, s, 2xCH$_3$N), 3.02-3.08 (1H, m, CHOC), 3.08-3.12 (2H, m, 2xCHOC), 3.12-3.17 (2H, m, CHOC, C$^{3''}$H), 3.19-3.31 (5H, m, C$^{2'}$H, 2xCH$_2$N), 3.34-3.39 (1H, m, C$^{3'}$H), 3.39-3.55 (3H, m, 2xCH$_a$H$_b$OH, CHOCH$_a$H$_b$CH$_2$) 3.56-3.71 (2H, m, 2xCH$_a$H$_b$OH), 3.71-3.84 (1H, m, CHOCH$_a$H$_b$CH$_2$), 4.27 (1H, d, *J* = 7.4 Hz, C$^{1'}$H), 4.28-4.35 (1H, m, OH), 4.38 (1H, dxd, *J* = 7.7, 2.7 Hz, C$^{1''}$H), 4.48 (1H, t, *J* = 6.1, OH), 4.78-4.98 (2H, m, OH), 5.02 (1H, br s, OH) 5.16 (1H, br s, OH), 5.42 (1H, br s, OH). **^{13}C NMR** (100 MHz, DMSO-d$_6$): δ_C 14.3 (CH$_2$CH$_3$), 22.1 (2xCH$_2$CH$_2$N), 22.3 (CH$_2$CH$_3$), 25.9 (CH$_2$(CH$_2$)$_2$), 26.2 (2xCH$_2$(CH$_2$)$_2$), 28.9 (CH$_2$(CH$_2$)$_2$), 29.2 (CH$_2$(CH$_2$)$_2$), 29.3 (CH$_2$(CH$_2$)$_2$), 29.7 (CHOCH$_2$CH$_2$), 31.1 (CH$_2$(CH$_2$)$_2$), 50.4 (2xCH$_3$N), 61.3 (2xCH$_2$OH), 63.4 (2xCH$_2$CH$_2$N), 69.1 (CHOCH$_2$CH$_2$), 70.2 (CHOC), 70.3 (CHOC), 75.3 (C$^{2''}$H), 76.4 (C$^{3'}$H), 76.5 (C$^{3''}$H), 77.0 (CHOC), 77.4 (CHOC), 82.6 (C$^{2'}$H), 101.7 (C$^{1'}$H), 104.5 (C$^{1''}$H). **HRMS (ESI):** *m/z* exact mass calculated for $C_{29}H_{58}NO_{11}$ [M-I$^-$]: 596.4004; found: 596.4004.

***N*,*N*-dimethyl-*N*-octyl-9-[(2’-*O*-β-D-glucopyranosyl-β-D-glucopyranosyl)oxy]nonaan-1-ammonium iodide (21a): (2.08 ppm acetonitrile peak is present)**

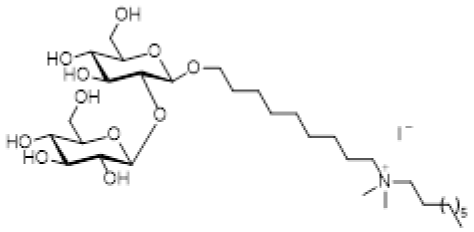

^{1}H NMR (400 MHz, DMSO-d$_6$): δ_H 0.88 (3H, t, *J* = 6.5 Hz, CH$_2$CH$_3$), 1.08-1.42 (20H, m, 9xCH$_2$(CH$_2$)$_2$, CH$_2$CH$_3$), 1.42-1.57 (2H, m, CHOCH$_2$CH$_2$), 1.58-1.73 (4H, m, 2xCH$_2$CH$_2$N), 2.97-3.04 (1H, m, C$^{2''}$H), 3.00 (6H, s, 2xCH$_3$N), 3.02-3.08 (1H, m, CHOC), 3.08-3.12 (2H, m, 2xCHOC), 3.12-3.17 (2H, m, CHOC, C$^{3''}$H), 3.18-3.28 (5H, m, C$^{2'}$H, 2xCH$_2$N), 3.30-3.41 (1H, m, C$^{3'}$H), 3.38-3.55 (3H, m, 2xCH$_a$H$_b$OH, CHOCH$_a$H$_b$CH$_2$), 3.56-3.71 (2H, m, 2xCH$_a$H$_b$OH), 3.71-3.84 (1H, m, CHOCH$_a$H$_b$CH$_2$), 4.27 (1H, d, *J* = 7.6 Hz, C$^{1'}$H), 4.28-4.36 (1H, m, OH), 4.38 (1H, d, *J* = 7.4 Hz, C$^{1''}$H), 4.49 (1H, t, *J* = 5.3, OH), 4.83-4.99 (2H, m, OH), 5.04 (1H, d, *J* = 4.1, OH) 5.16 (1H, d, *J* = 2.5, OH), 5.43 (1H, d, *J* = 2.9, OH). **^{13}C NMR** (100 MHz, DMSO-d$_6$): δ_C 14.4 (CH$_2$CH$_3$), 22.1 (2xCH$_2$CH$_2$N), 22.5 (CH$_2$CH$_3$), 25.9 (CH$_2$(CH$_2$)$_2$), 26.2 (2xCH$_2$(CH$_2$)$_2$), 28.9 (3xCH$_2$(CH$_2$)$_2$), 29.2 (CH$_2$(CH$_2$)$_2$), 29.3 (CH$_2$(CH$_2$)$_2$), 29.7 (CHOCH$_2$CH$_2$), 31.6 (CH$_2$(CH$_2$)$_2$), 50.4 (2xCH$_3$N), 61.3 (2xCH$_2$OH), 63.4 (2xCH$_2$CH$_2$N), 69.1 (CHOCH$_2$CH$_2$), 70.2 (CHOC), 70.3 (CHOC), 75.3 (C$^{2''}$H), 76.4 (C$^{3'}$H), 76.5 (C$^{3''}$H), 77.0 (CHOC), 77.4 (CHOC), 82.6 (C$^{2'}$H), 101.7 (C$^{1'}$H), 104.5 (C$^{1''}$H). **HRMS (ESI):** *m/z* exact mass calculated for $C_{31}H_{62}NO_{11}$ [M-I$^-$]: 624.4317; found: 624.4314.

***N*,*N*-dimethyl-*N*-dodecyl-9-[(2’-*O*-β-D-glucopyranosyl-β-D-glucopyranosyl)oxy]nonaan-1-ammonium iodide (22a): (2.08 ppm acetone or acetonitrile is present)**

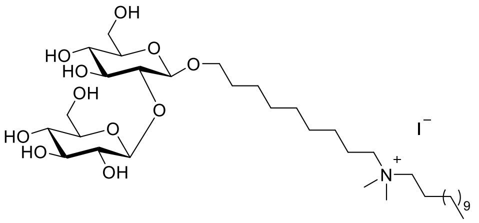

^{1}H NMR (400 MHz, DMSO-d_6): δ_H 0.86 (3H, t, *J* = 6.8 Hz, $CH_2C\underline{H}_3$), 1.14-1.42 (28H, m, 13x$C\underline{H}_2(CH_2)_2$, $C\underline{H}_2CH_3$), 1.42-1.57 (2H, m, $CHOCH_2C\underline{H}_2$), 1.57-1.73 (4H, m, 2x$C\underline{H}_2CH_2N$), 2.96-3.03 (1H, m, $C^{2''}\underline{H}$), 2.99 (6H, s, 2x$C\underline{H}_3N$), 3.03-3.08 (1H, m, $C\underline{H}OC$), 3.08-3.12 (2H, m, 2x$C\underline{H}OC$), 3.12-3.18 (2H, m, $C\underline{H}OC$, $C^{3''}\underline{H}$), 3.18-3.27 (5H, m, $C^{2'}\underline{H}$, 2x$C\underline{H}_2N$), 3.32-3.54 (4H, m, $C^{3'}\underline{H}$, 2x$C\underline{H}_aH_bOH$, $CHOC\underline{H}_aH_bCH_2$), 3.56-3.70 (2H, m, 2x$CH_a\underline{H}_bOH$), 3.70-3.80 (1H, m, $CHOCH_a\underline{H}_bCH_2$), 4.26 (1H, d, *J* = 7.6 Hz, $C^{1'}\underline{H}$), 4.28-4.36 (1H, m, $O\underline{H}$), 4.37 (1H, dxd, *J* = 7.7, 2.7 Hz, $C^{1''}\underline{H}$), 4.49 (1H, m, $O\underline{H}$), 4.83-4.97 (2H, m, $O\underline{H}$), 5.03 (1H, m, $O\underline{H}$) 5.16 (1H, m, $O\underline{H}$), 5.43 (1H, m, $O\underline{H}$). **^{13}C NMR** (100 MHz, DMSO-d_6): δ_C 14.4 ($CH_2\underline{C}H_3$), 22.1 (2x$\underline{C}H_2CH_2N$), 22.5 ($\underline{C}H_2CH_3$), 25.9 ($\underline{C}H_2(CH_2)_2$), 26.2 (2x$\underline{C}H_2(CH_2)_2$), 28.9 ($\underline{C}H_2(CH_2)_2$), 29.1-29.6 (8x$\underline{C}H_2(CH_2)_2$), 29.7 ($CHOCH_2\underline{C}H_2$), 31.7 ($\underline{C}H_2(CH_2)_2$), 50.4 (2x$\underline{C}H_3N$), 61.3 (2x$\underline{C}H_2OH$), 63.4 (2x$CH_2\underline{C}H_2N$), 69.1 ($CHO\underline{C}H_2CH_2$), 70.2 ($\underline{C}HOC$), 70.3 ($\underline{C}HOC$), 75.3 ($\underline{C}^{2''}H$), 76.4 ($\underline{C}^{3'}H$), 76.5 ($\underline{C}^{3''}H$), 77.0 ($\underline{C}HOC$), 77.4 ($\underline{C}HOC$), 82.6 ($\underline{C}^{2'}H$), 101.7 ($\underline{C}^{1'}H$), 104.5 ($\underline{C}^{1''}H$). **HRMS (ESI):** *m/z* exact mass calculated for $C_{35}H_{70}NO_{11}$ [M-I$^-$]: 680.4943; found: 680.4932.

***N*,*N*-dimethyl- *N*-octadecyl-9-[(2'-*O*-β-D-glucopyranosyl-β-D-glucopyranosyl)oxy]nonaan-1-ammonium iodide (23a):**

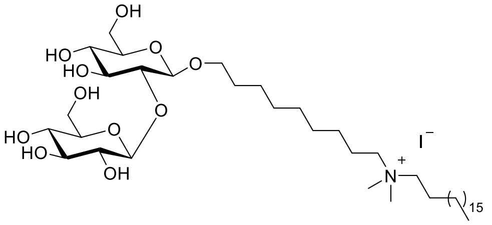

^{1}H NMR (400 MHz, DMSO-d_6): δ_H 0.86 (3H, t, *J* = 6.8 Hz, $CH_2C\underline{H}_3$), 1.05-1.40 (40H, m, 18x$C\underline{H}_2(CH_2)_2$, $C\underline{H}_2CH_3$), 1.43-1.57 (2H, m, $CHOCH_2C\underline{H}_2$), 1.57-1.73 (4H, m, 2x$C\underline{H}_2CH_2N$), 2.97-3.04 (1H, m, $C^{2''}\underline{H}$), 2.99 (6H, s, 2x$C\underline{H}_3N$), 3.02-3.08 (1H, m, $C\underline{H}OC$), 3.08-3.12 (2H, m, 2x$C\underline{H}OC$), 3.12-3.17 (2H, m, $C\underline{H}OC$, $C^{3''}\underline{H}$), 3.17-3.26 (5H, m, $C^{2'}\underline{H}$, 2x$C\underline{H}_2N$), 3.38-3.55 (4H, m, $C^{3'}\underline{H}$, $C\underline{H}_aH_bOH$, $CHOC\underline{H}_aH_bCH_2$), 3.58-3.71 (2H, m, 2x$CH_a\underline{H}_bOH$), 3.71-3.81 (1H, m, $CHOCH_a\underline{H}_bCH_2$), 4.26 (1H, d, *J* = 7.6 Hz, $C^{1'}\underline{H}$), 4.37 (1H, dxd, *J* = 7.7, 2.7 Hz, $C^{1''}\underline{H}$), 4.34 (1H, m, $O\underline{H}$), 4.50 (1H, t, *J* = 6.1, $O\underline{H}$), 4.90 (1H, d, *J* = 4.2, $O\underline{H}$) 4.95 (1H, d, *J* = 3.7, $O\underline{H}$), 5.05 (1H, d, *J* = 4.6, $O\underline{H}$) 5.17 (1H, d, *J* = 3.3, $O\underline{H}$), 5.44 (1H, d, *J* = 3.3, $O\underline{H}$). **^{13}C NMR** (100 MHz, DMSO-d_6): δ_C 14.4 ($CH_2\underline{C}H_3$), 22.1 (2x$\underline{C}H_2CH_2N$), 22.5 ($\underline{C}H_2CH_3$), 25.9 ($\underline{C}H_2(CH_2)_2$), 26.2 (2x$\underline{C}H_2(CH_2)_2$), 28.9 ($\underline{C}H_2(CH_2)_2$), 29.1-29.6 (14x$\underline{C}H_2(CH_2)_2$), 29.7 ($CHOCH_2\underline{C}H_2$), 31.7 ($\underline{C}H_2(CH_2)_2$), 50.4 (2x$\underline{C}H_3N$), 61.3 (2x$\underline{C}H_2OH$), 63.4 (2x$CH_2\underline{C}H_2N$), 69.1 ($CHO\underline{C}H_2CH_2$), 70.2 ($\underline{C}HOC$), 70.3 ($\underline{C}HOC$), 75.3 ($\underline{C}^{2''}H$), 76.4 ($\underline{C}^{3'}H$), 76.5 ($\underline{C}^{3''}H$), 77.0 ($\underline{C}HOC$), 77.4 ($\underline{C}HOC$), 82.6 ($\underline{C}^{2'}H$), 101.7 ($\underline{C}^{1'}H$), 104.5 ($\underline{C}^{1''}H$). **HRMS (ESI):** *m/z* exact mass calculated for $C_{41}H_{82}NO_{11}$ [M-I$^-$]: 764.5887; found: 764.5883.

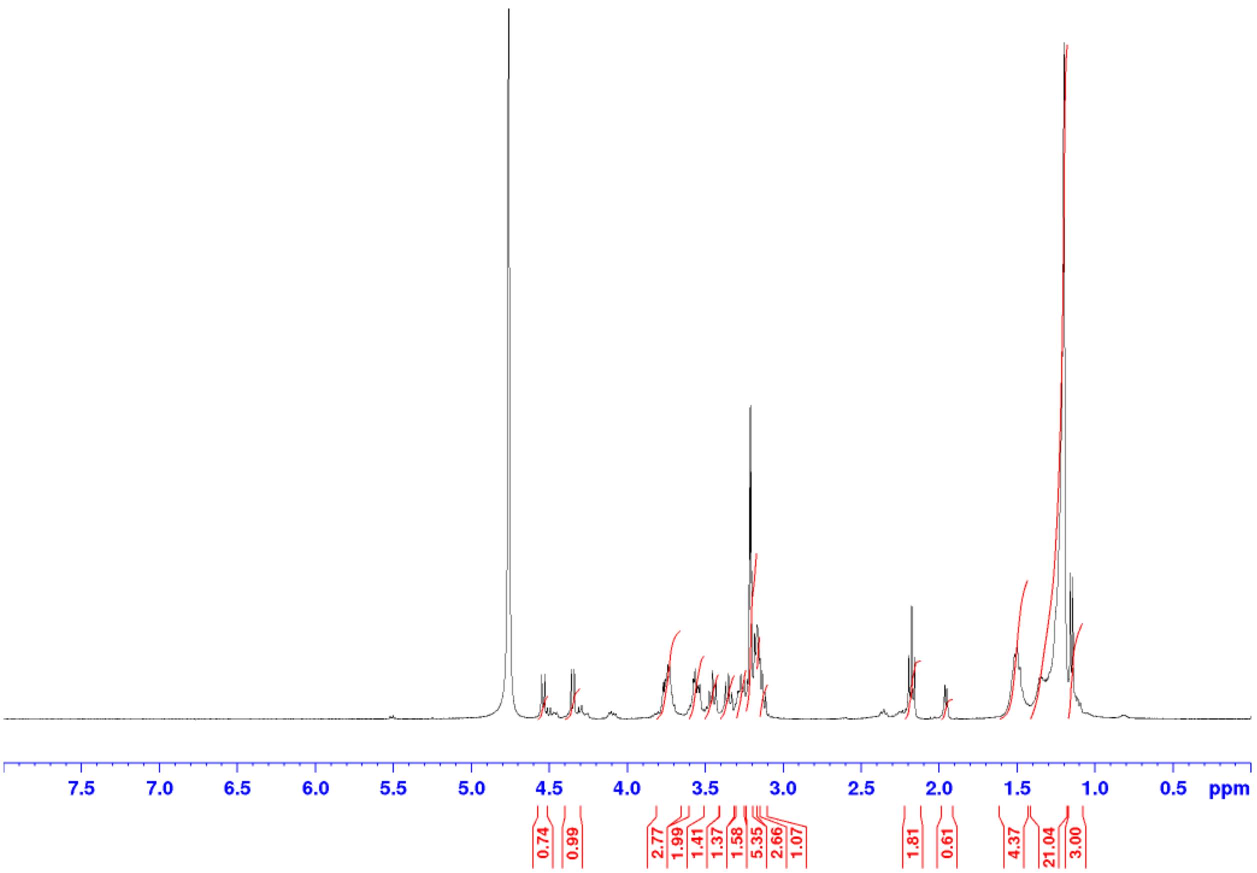

Figure 2. ^{1}**H-NMR 6**

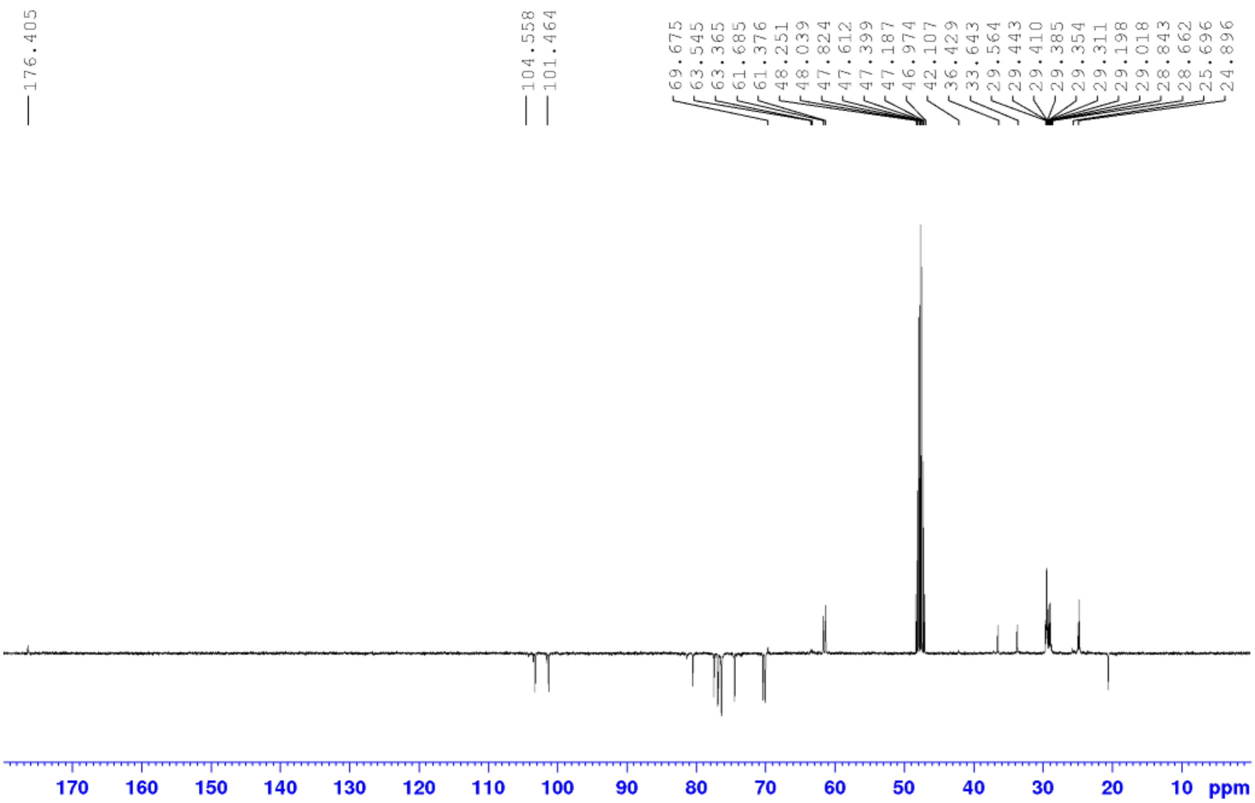

Figure 3. ^{13}C-NMR 6

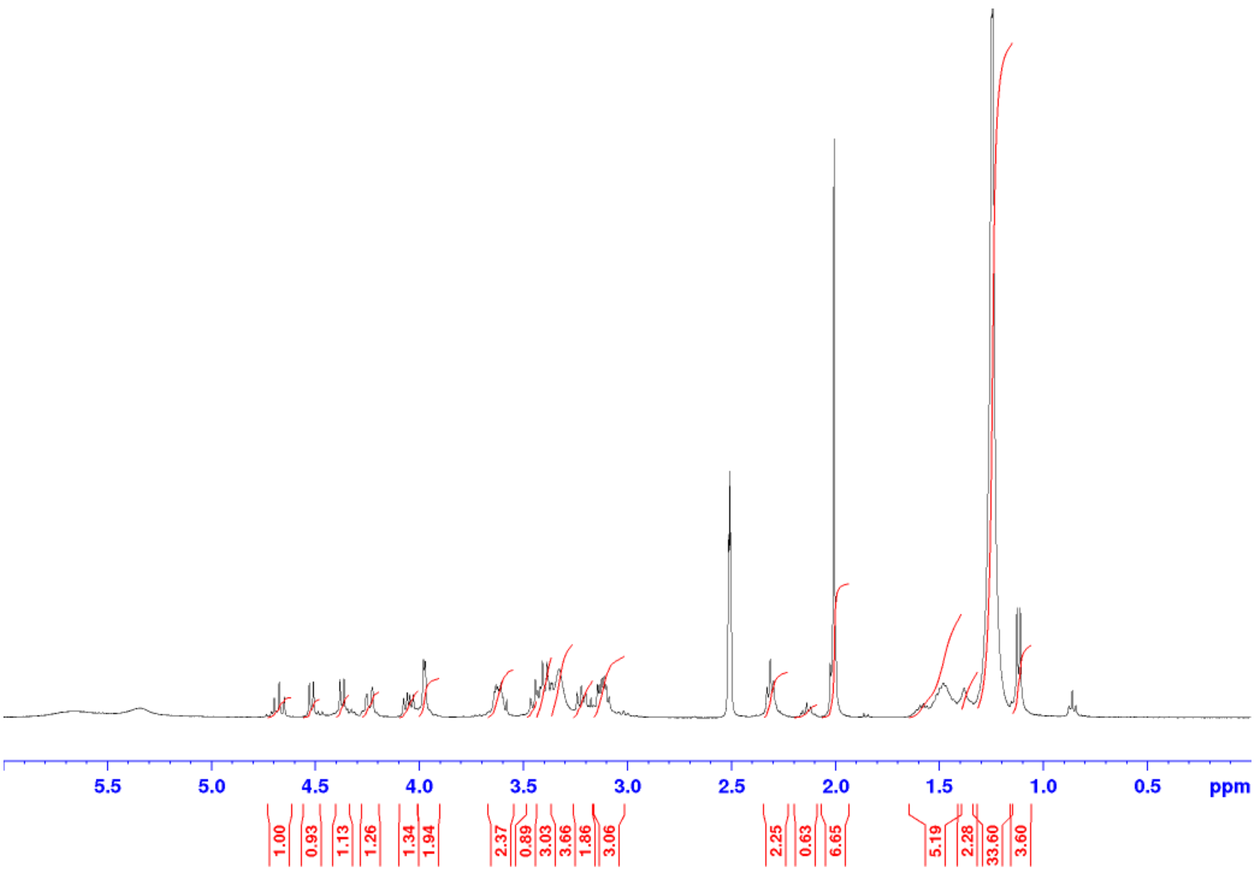

Figure 4. ^{1}**H- NMR 7**

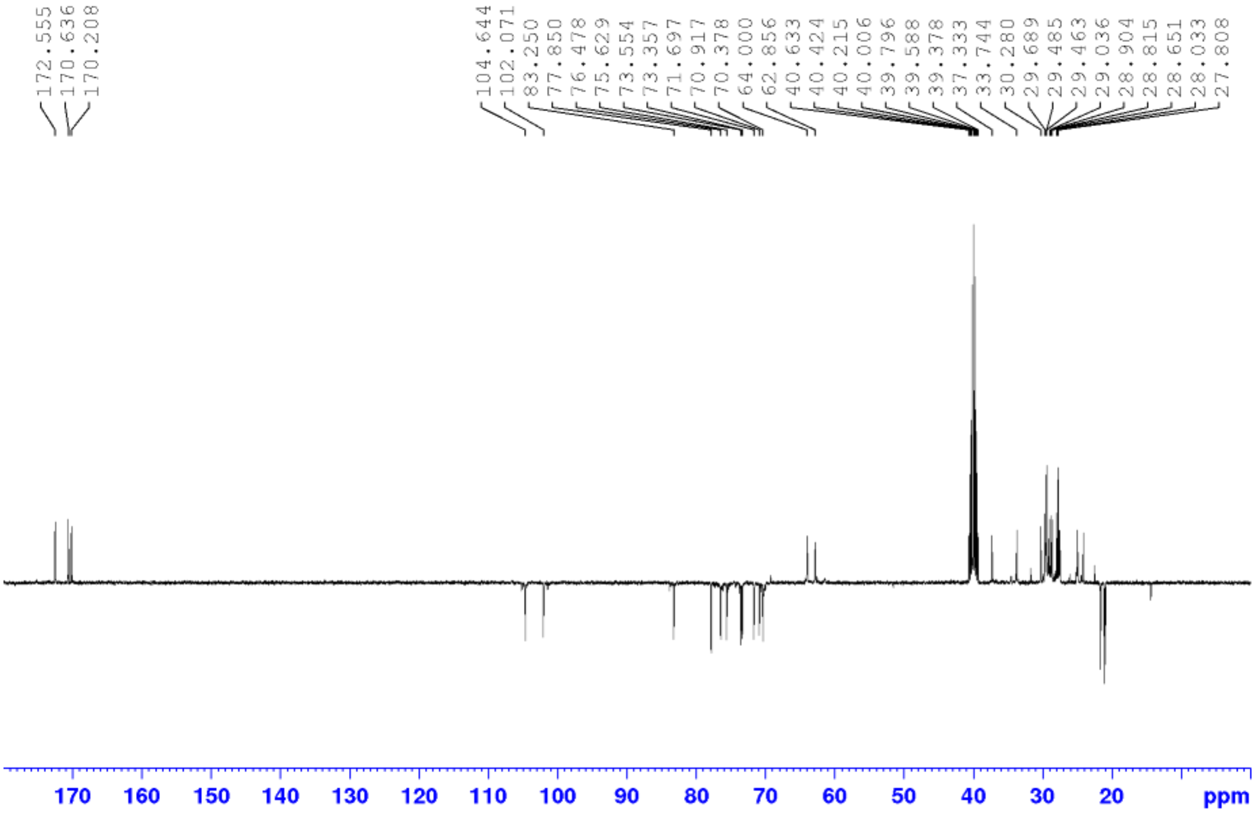

Figure 5. **^{13}C- NMR 7**

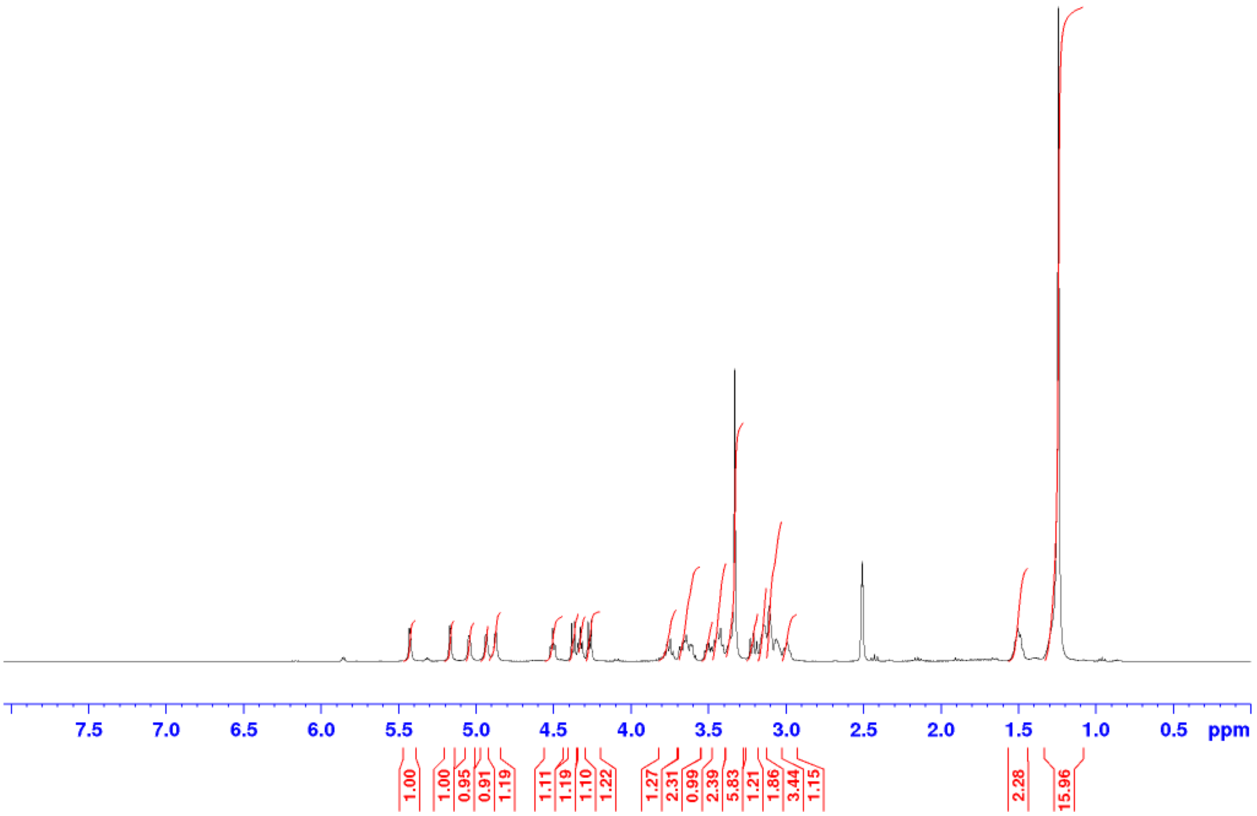

Figure 6. ^{1}H- NMR 8a

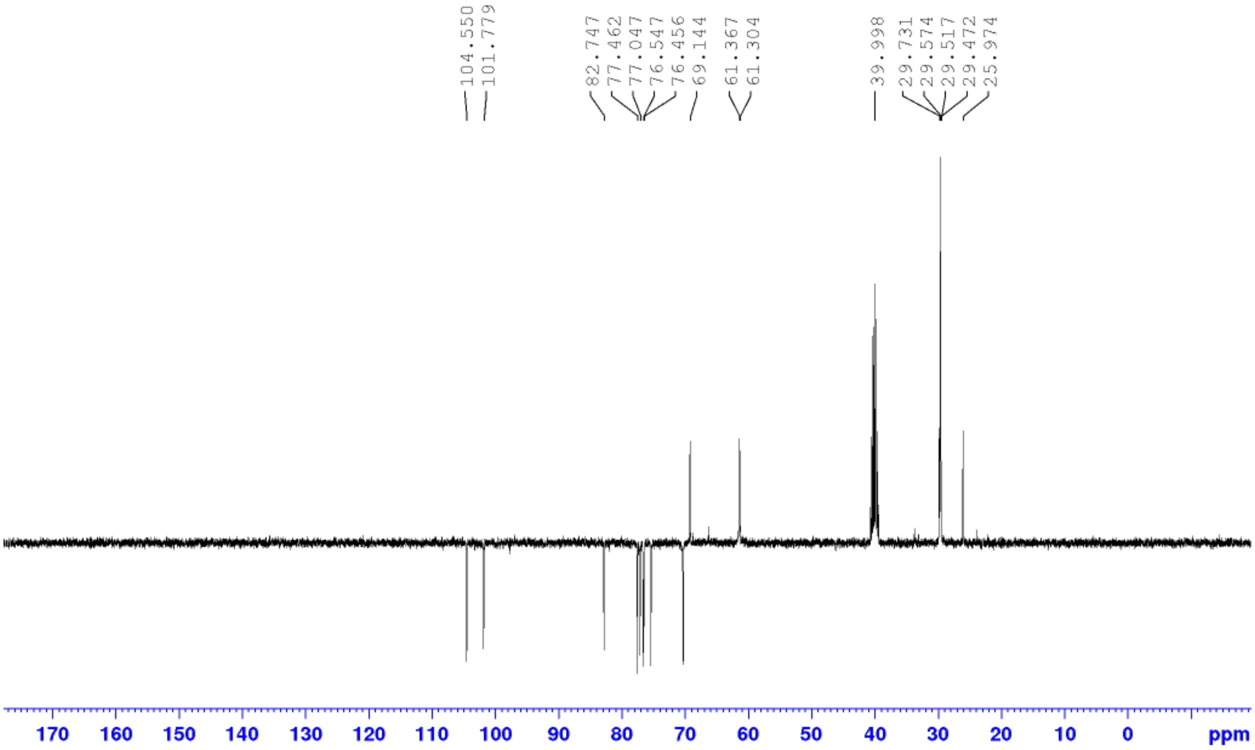

Figure 7. ^{13}C**- NMR 8a**

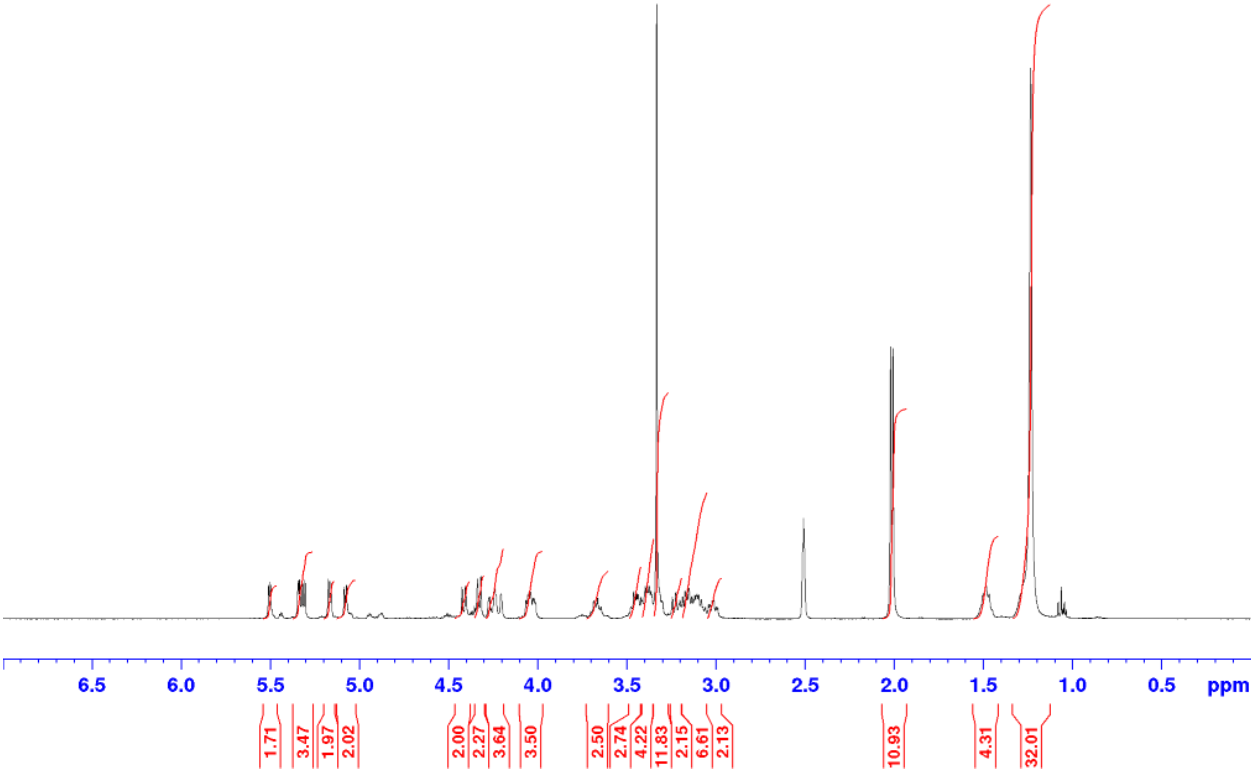

Figure 8. ^{1}**H-NMR 8b**

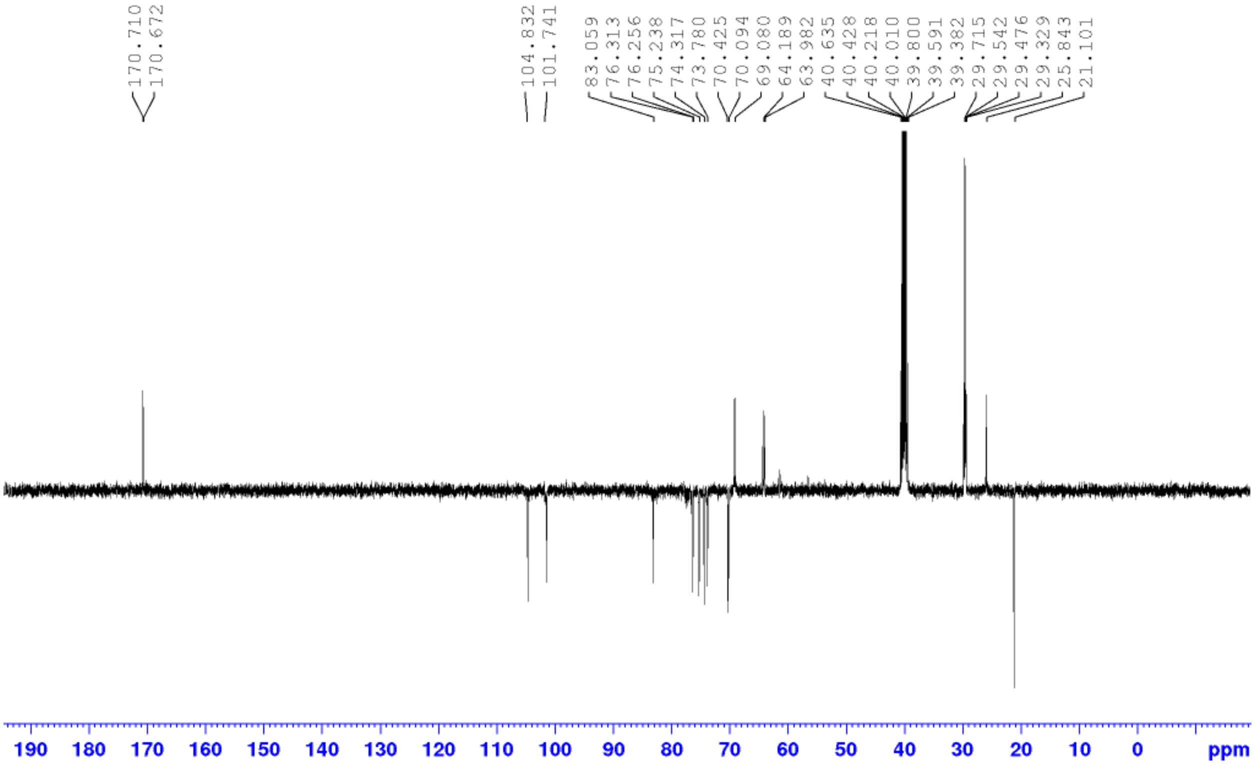

Figure 9. ^{13}**C-NMR 8b**

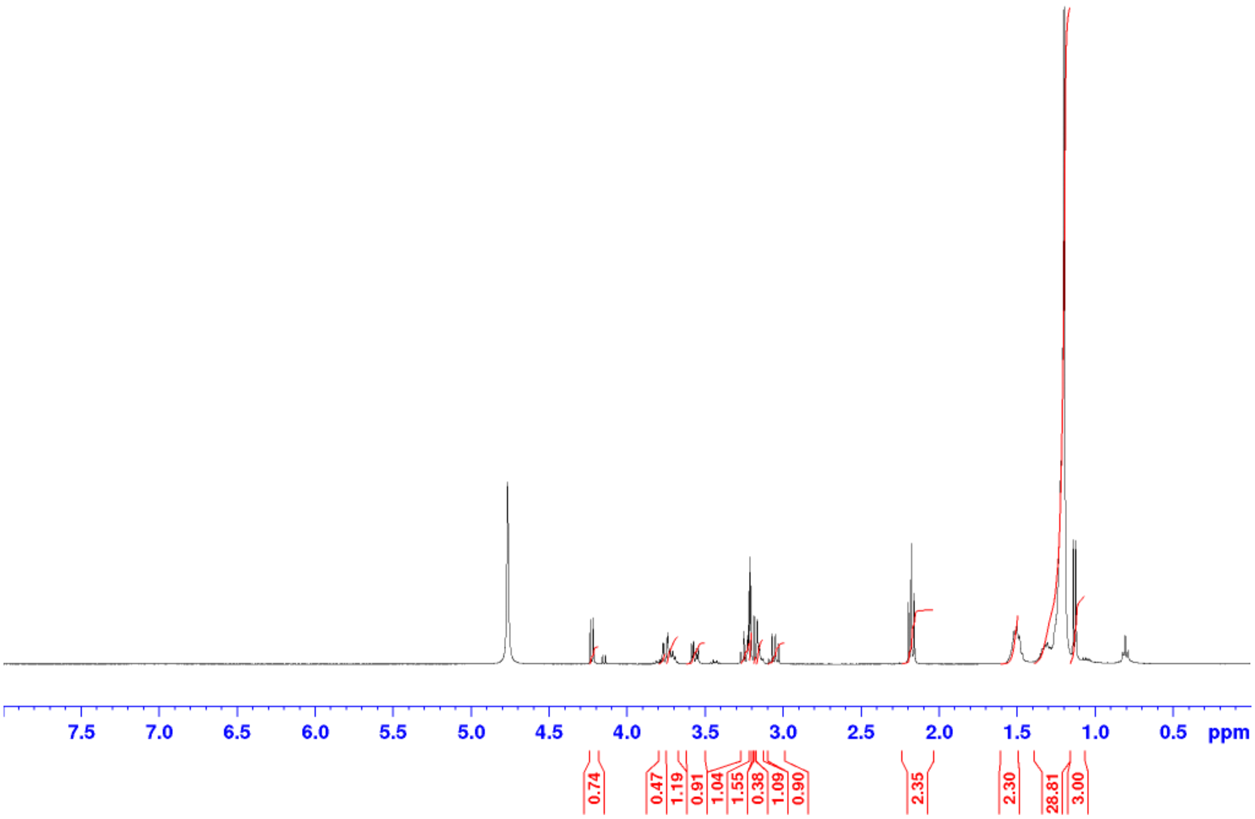

Figure 10. ^{1}**H-NMR 9a**

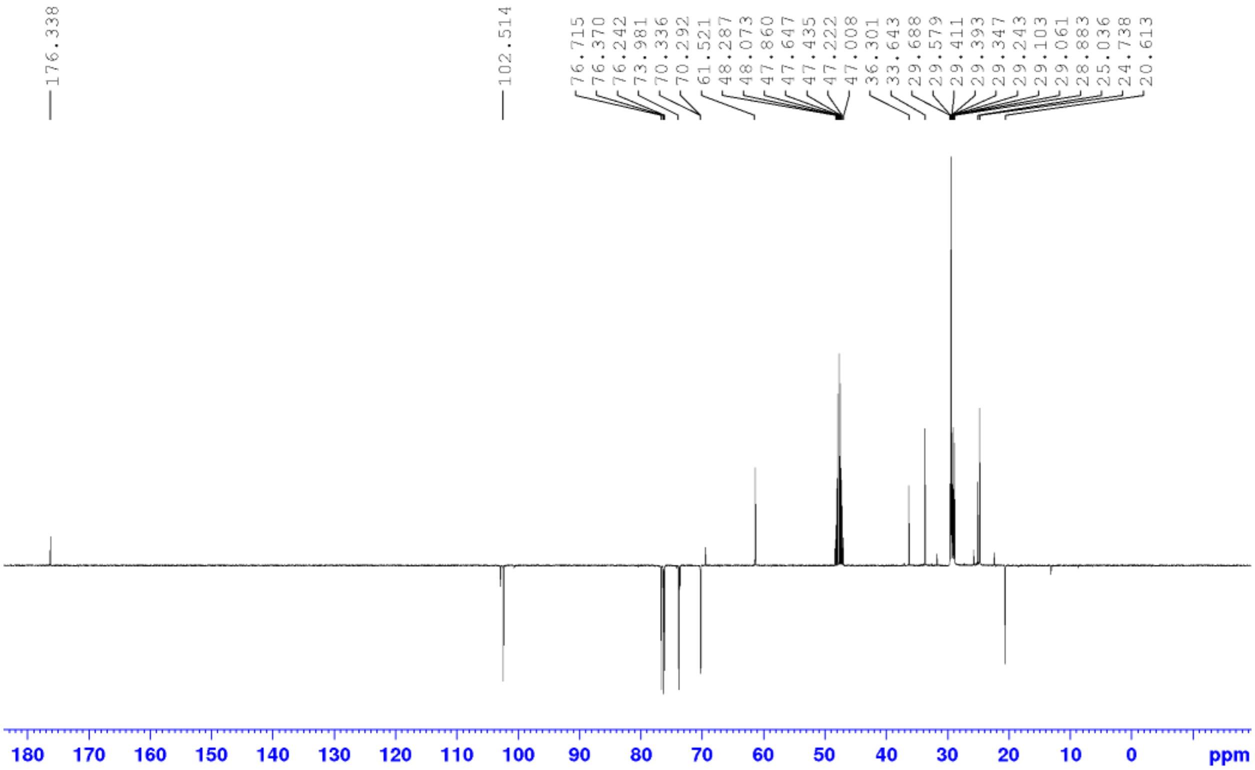

Figure 11. ^{13}**C-NMR 9a**

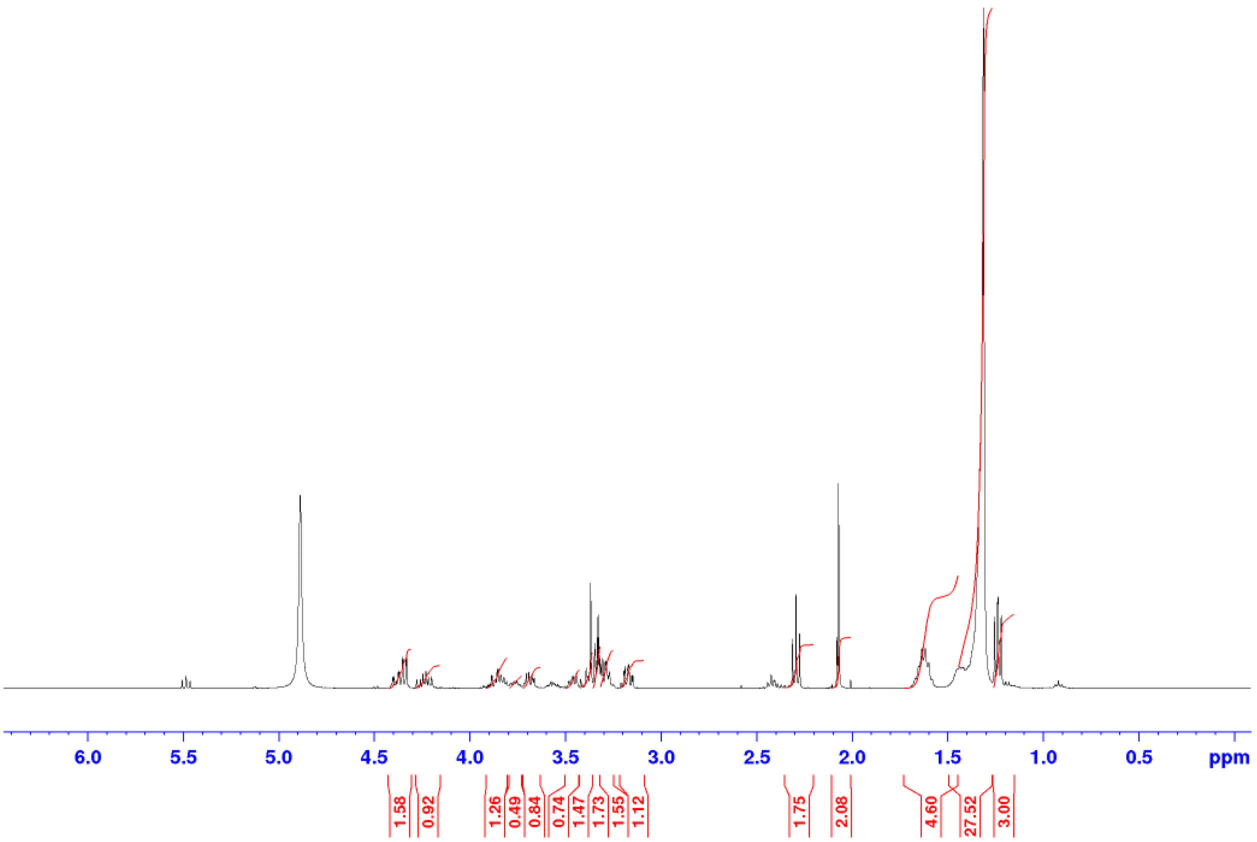

Figure 12. ^{1}**H-NMR 9b**

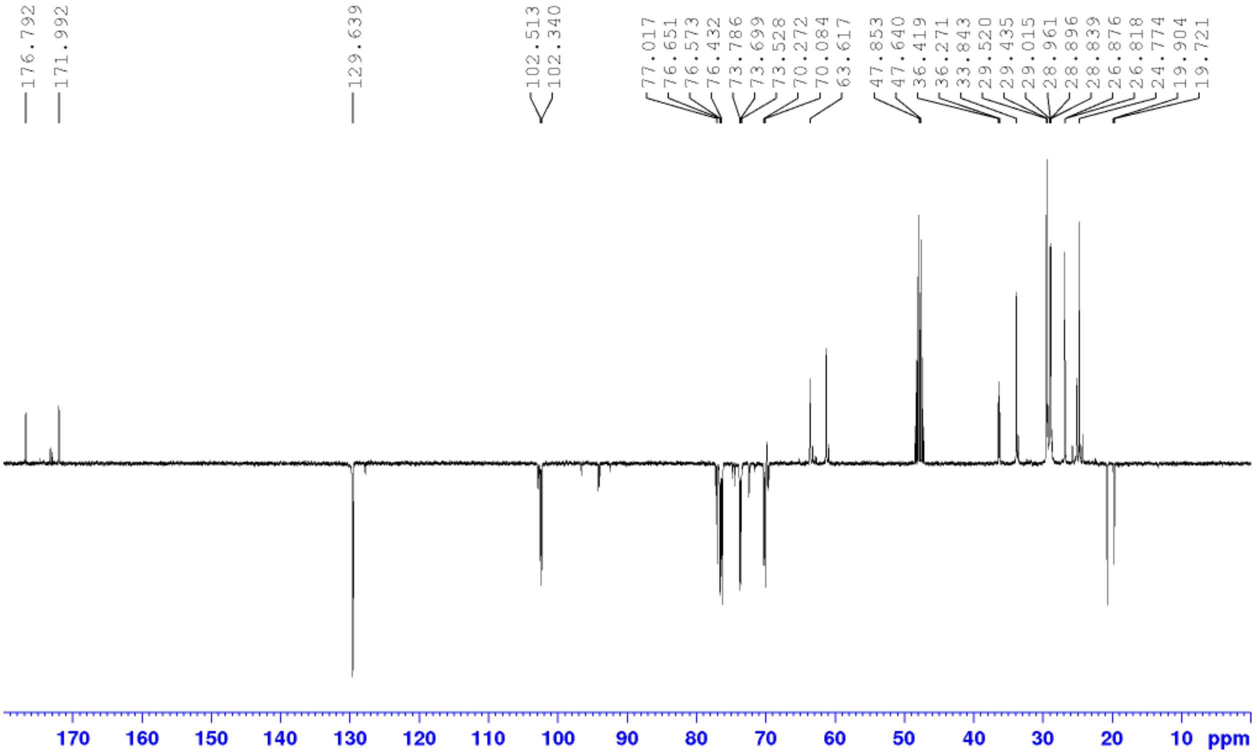

Figure 13. **^{13}C-NMR 9b**

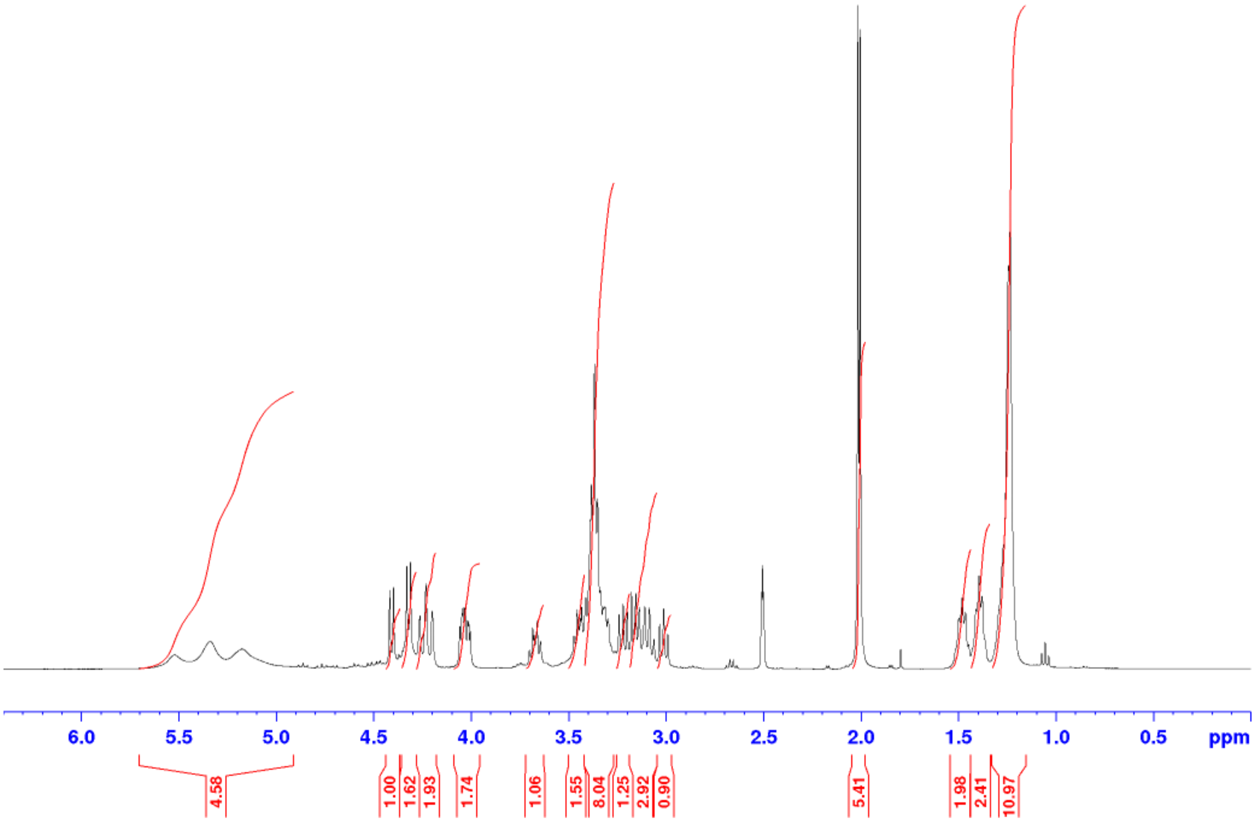

Figure 14. **^{1}H-NMR 11b**

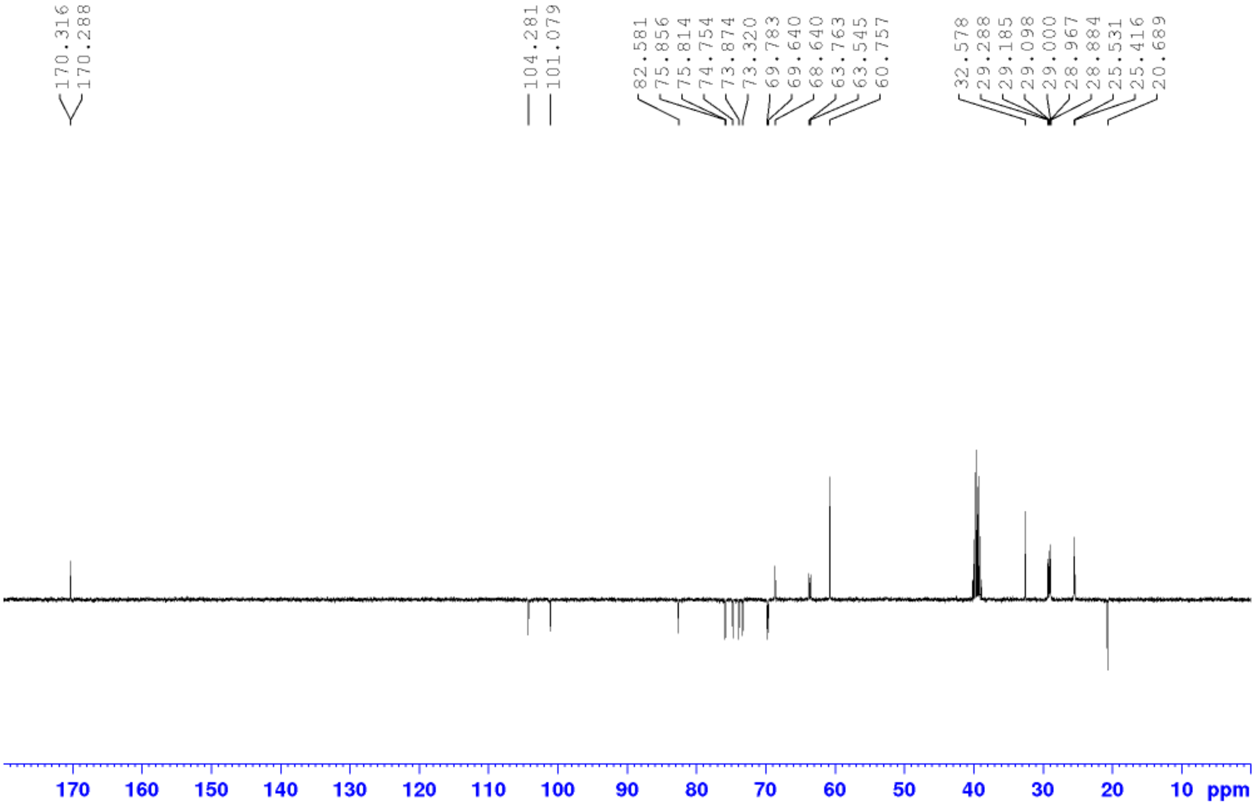

Figure 15. **^{1}H-NMR 11b**

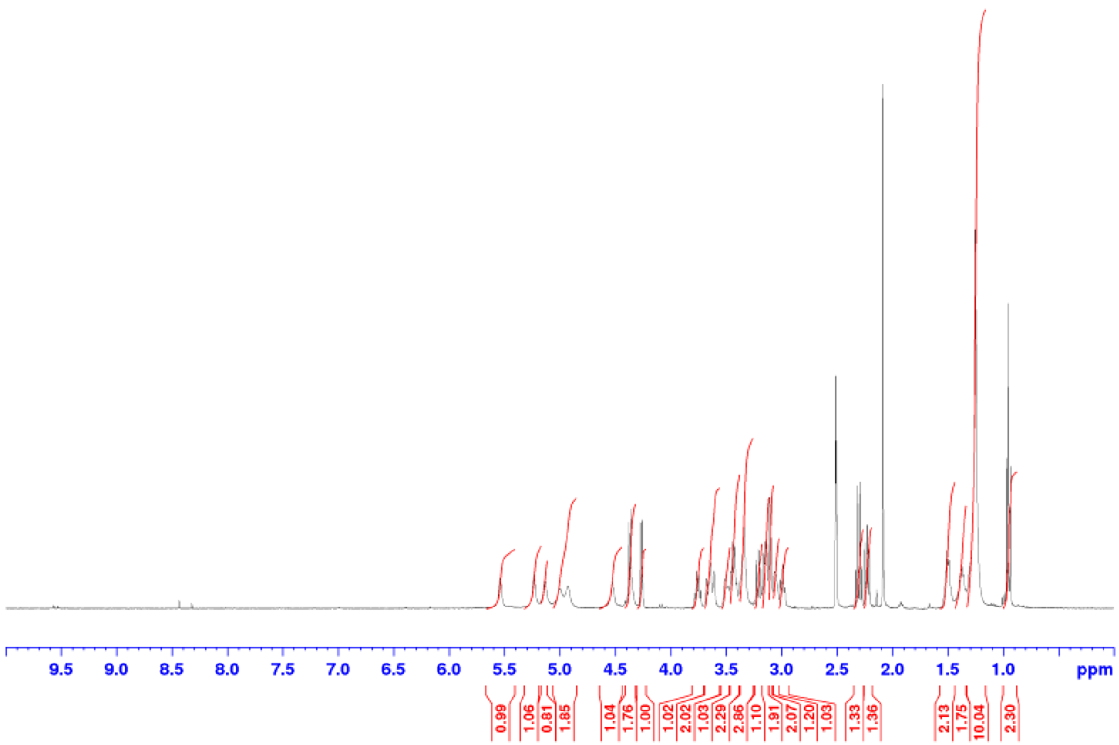

Figure 16. ^{1}H-NMR 12a

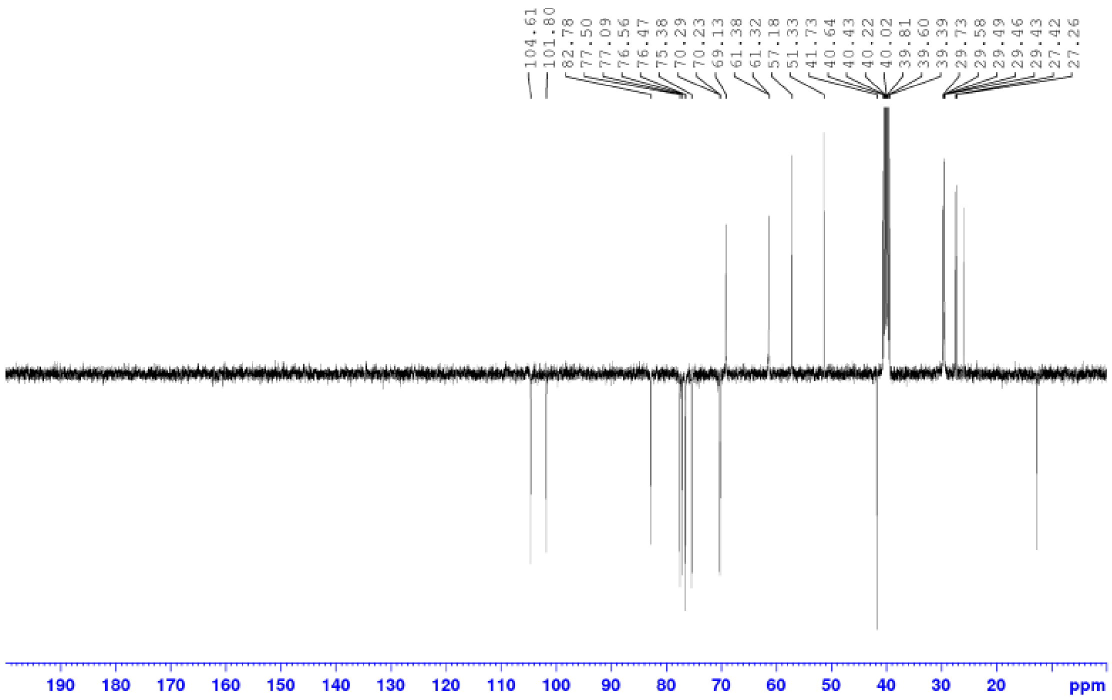

Figure 17. ^{13}**C-NMR 12a**

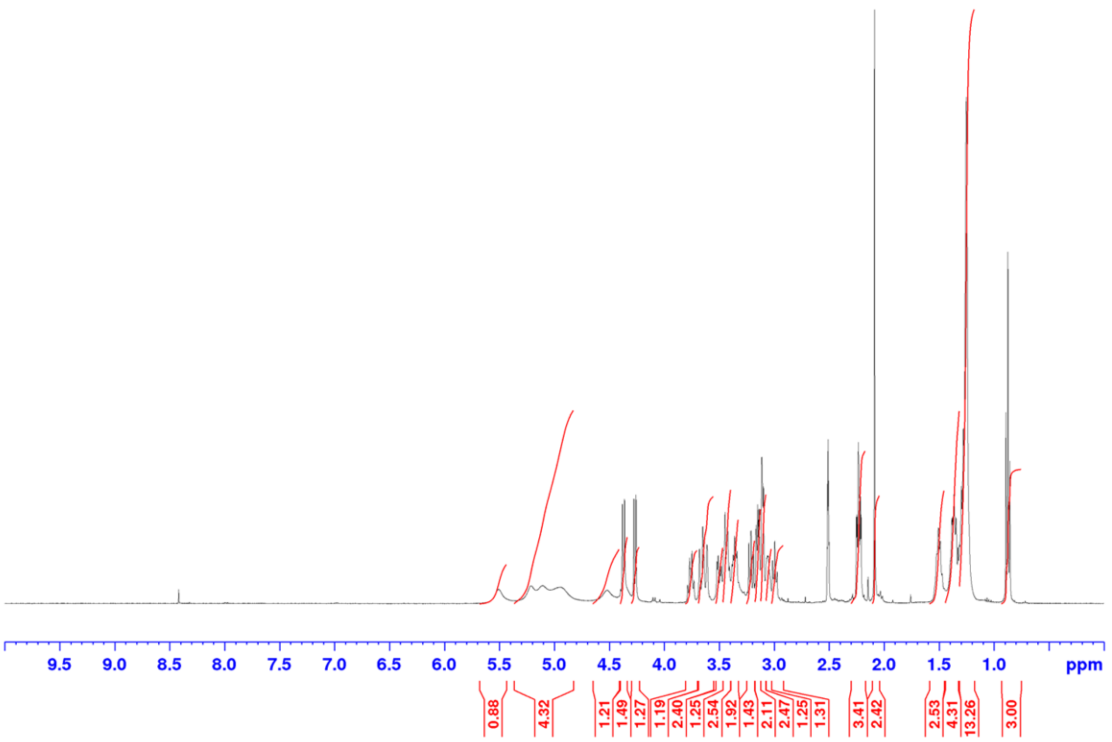

Figure 18. ^{1}**H-NMR 13a**

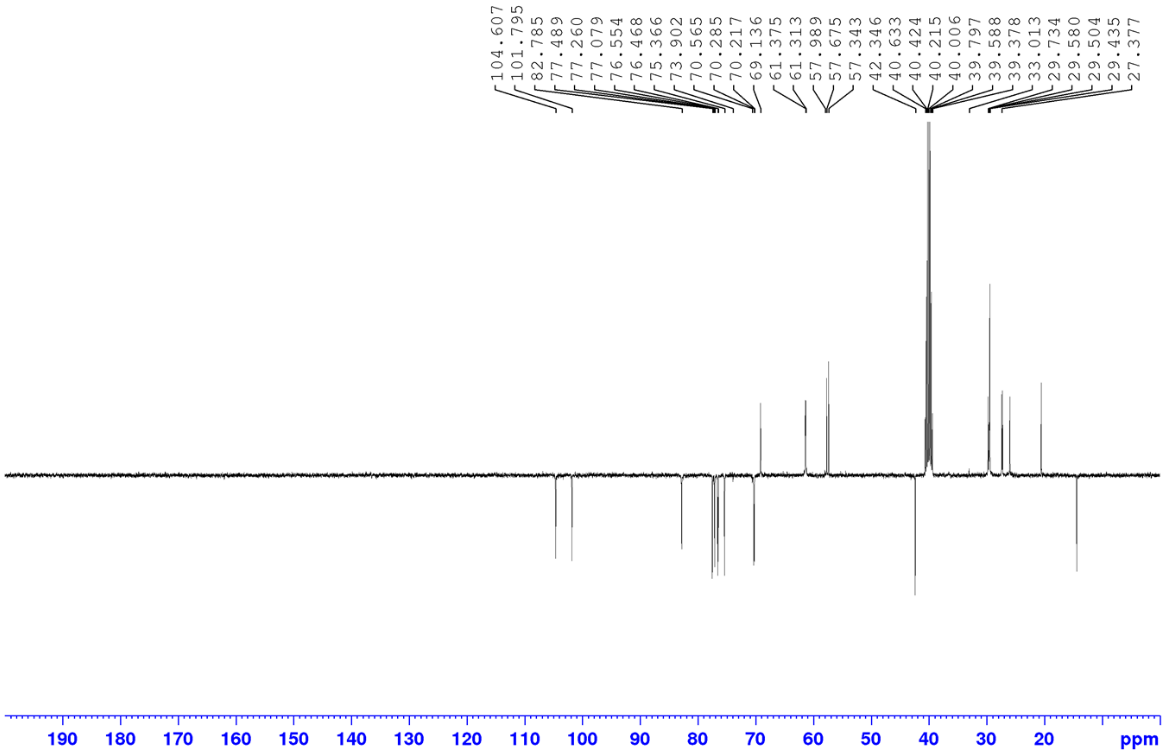

Figure 19. ^{13}**C-NMR of 13a**

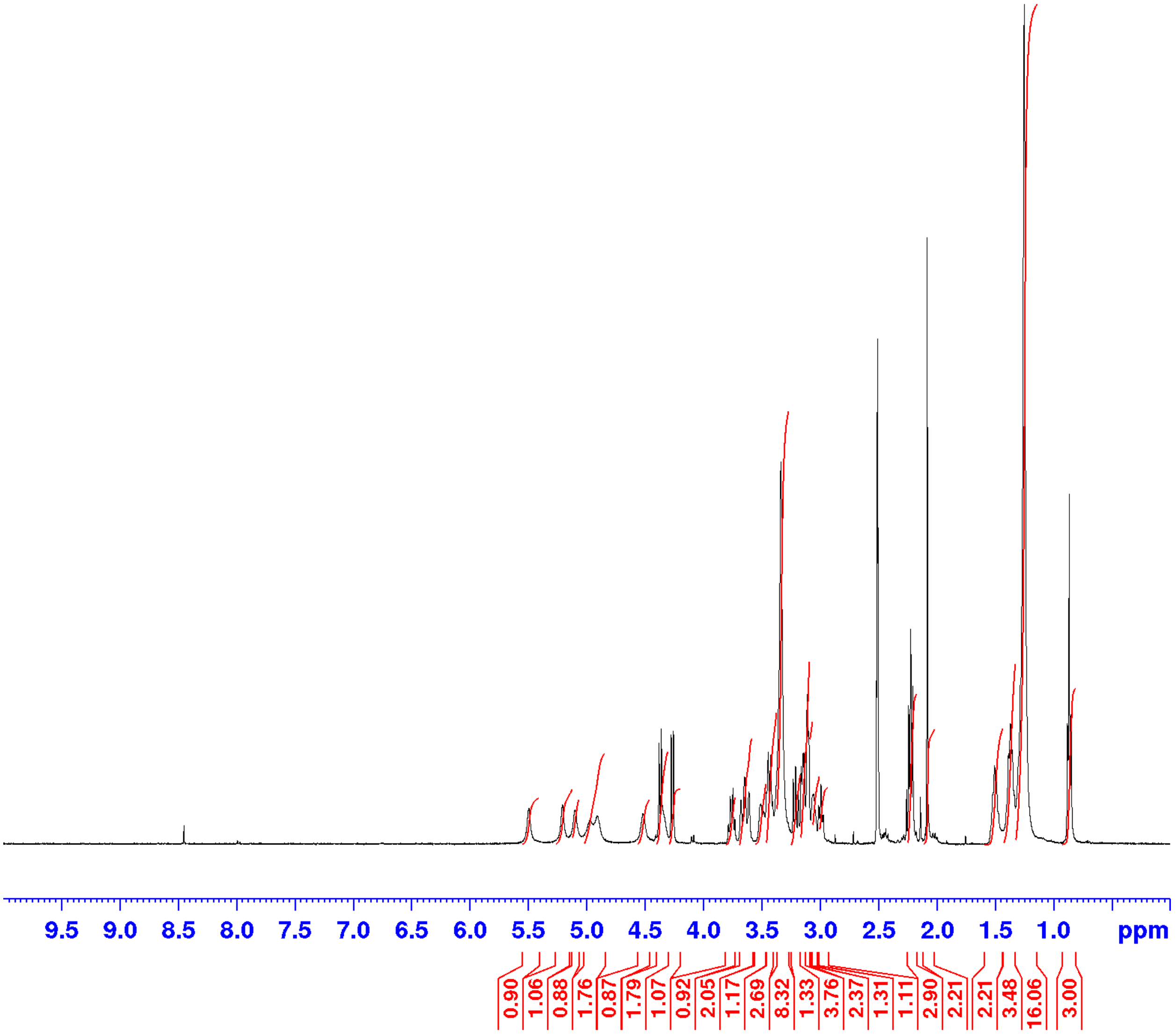

Figure 20. ^{1}H-NMR **14a**

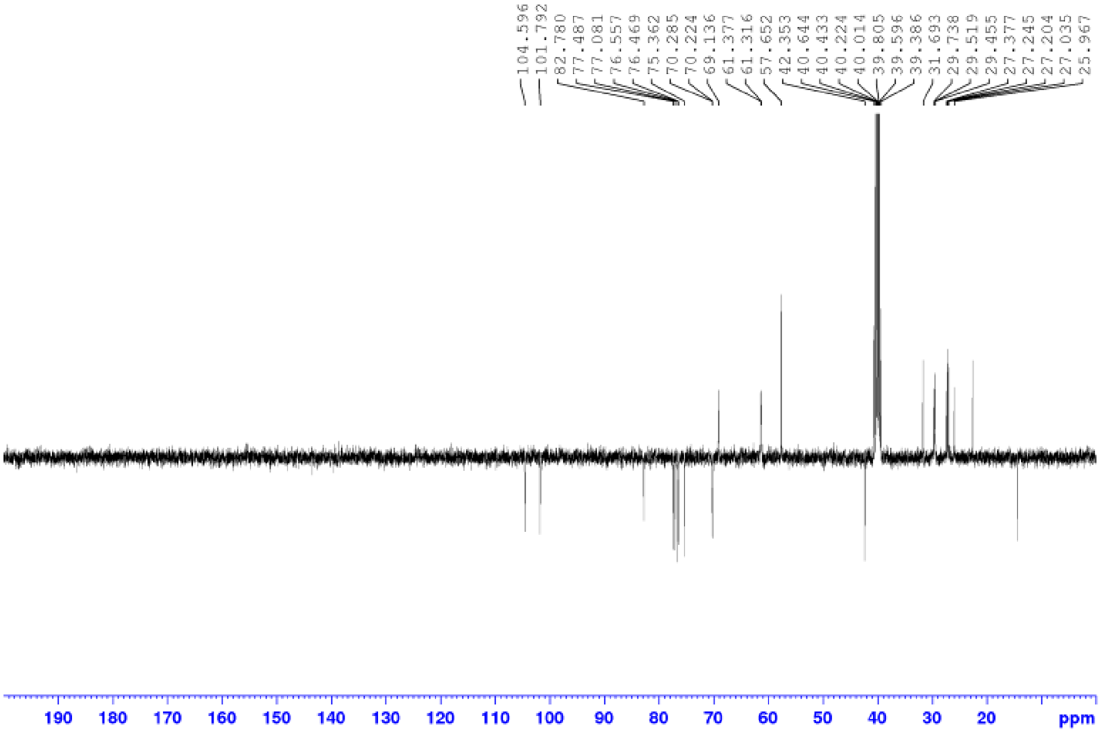

Figure 21. ^{13}**C-NMR 14a**

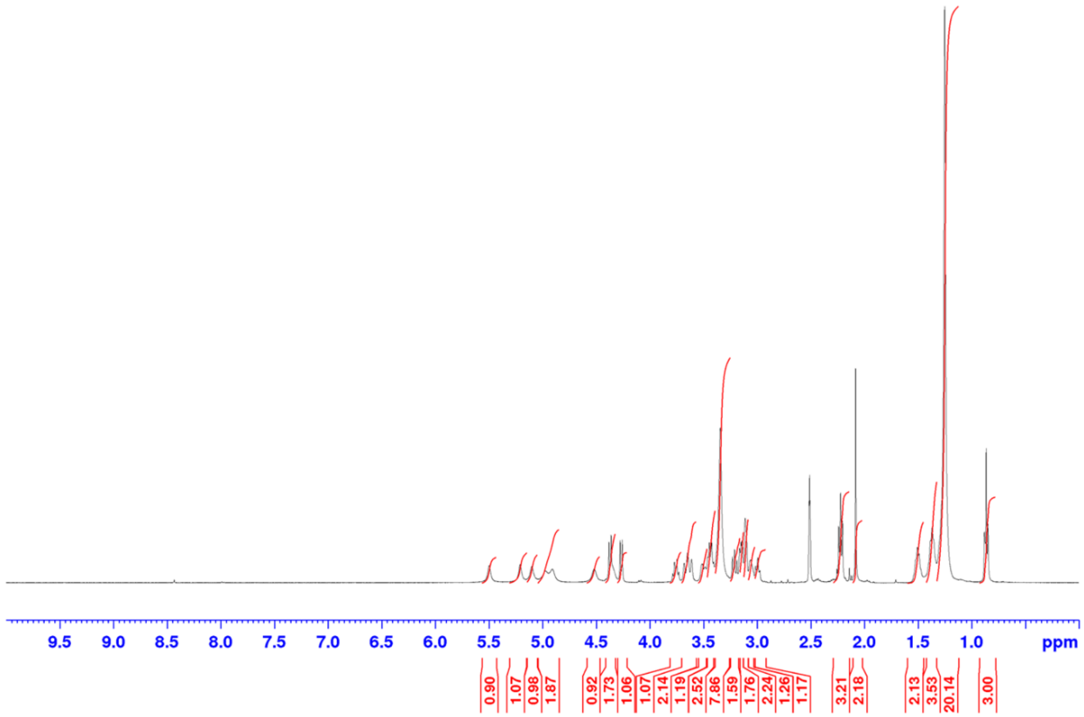

Figure 22. ^{1}**H-NMR 15a**

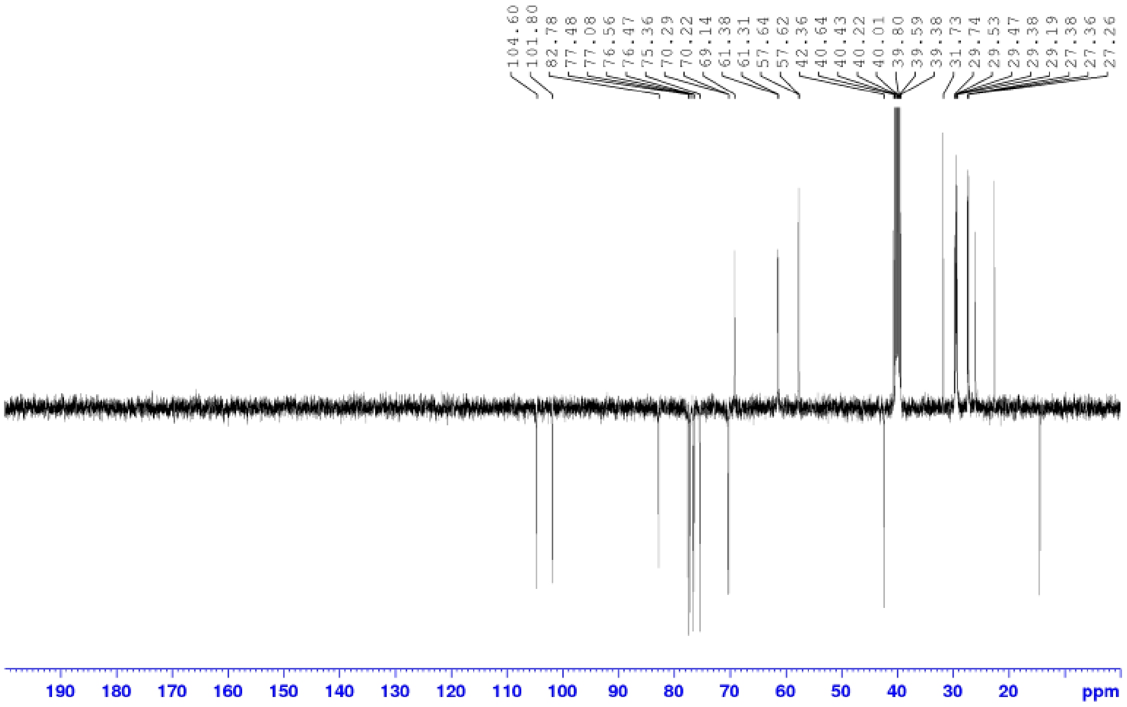

Figure 23. ^{13}**C-NMR 15a**

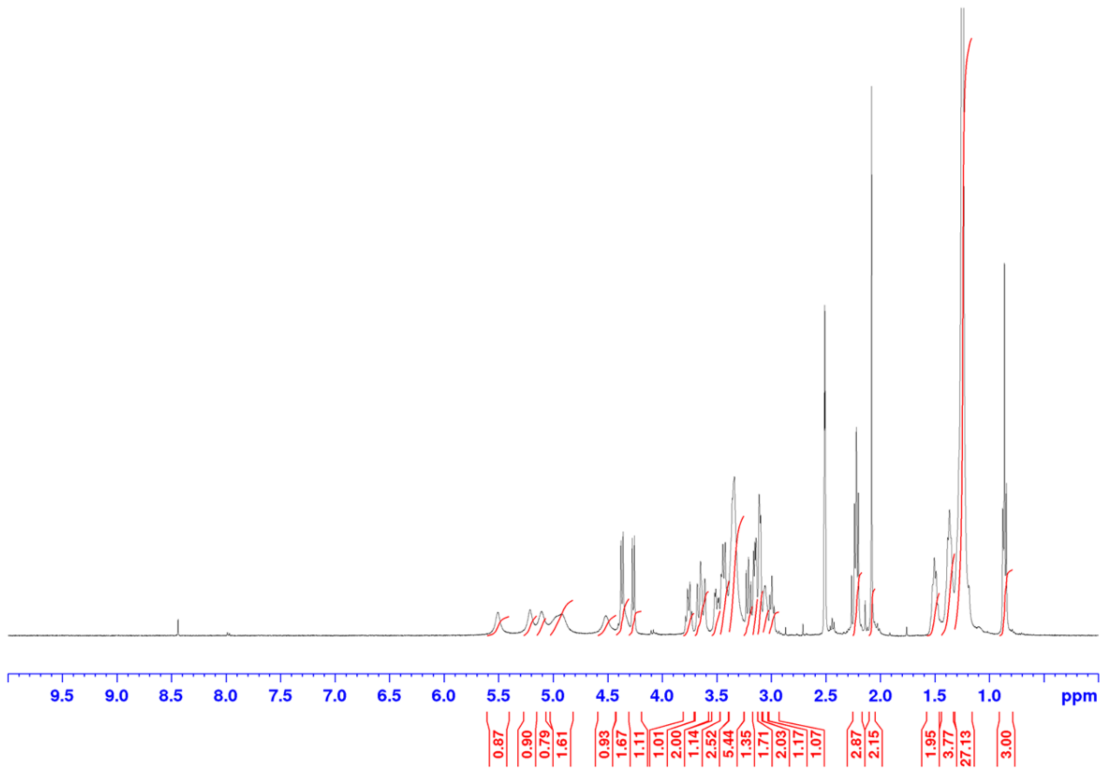

Figure 24. ^{1}H-NMR 16a

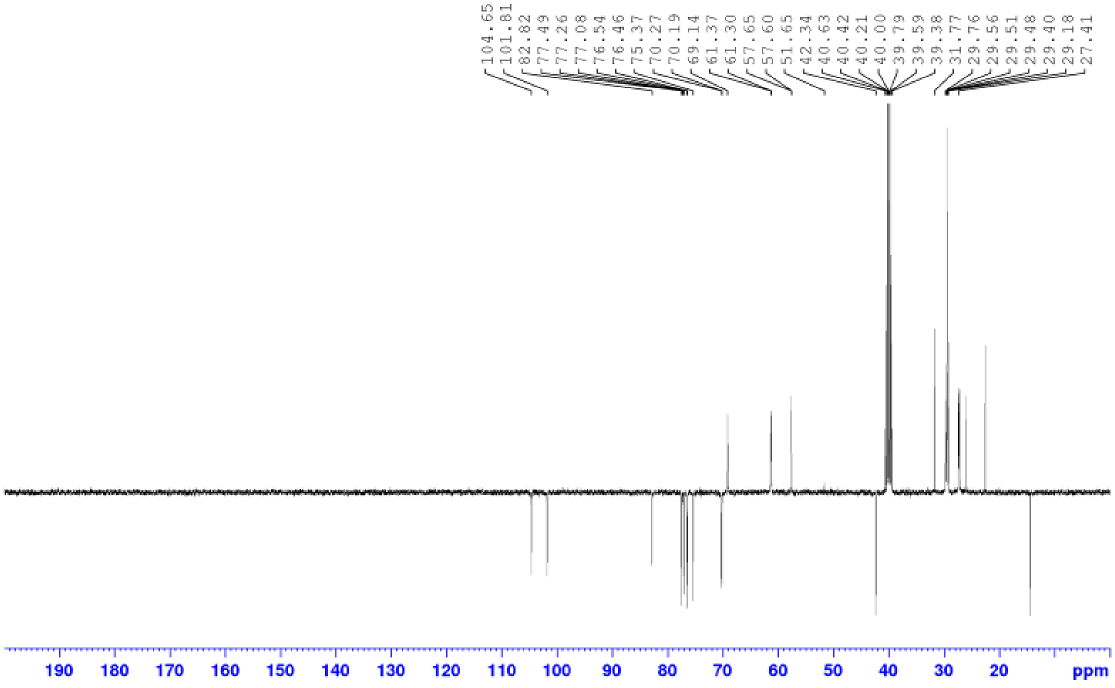

Figure 25. ^{13}**C-NMR 16a**

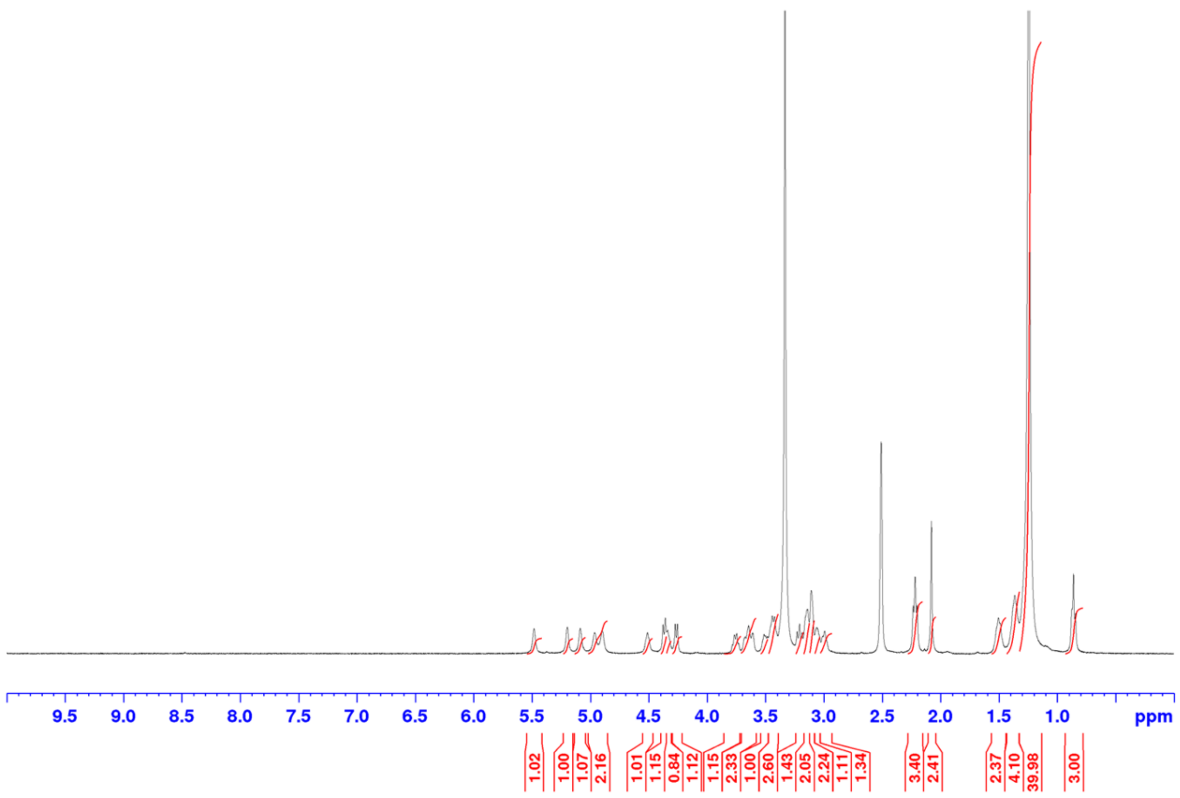

Figure 26. ^{1}H-NMR **17a**

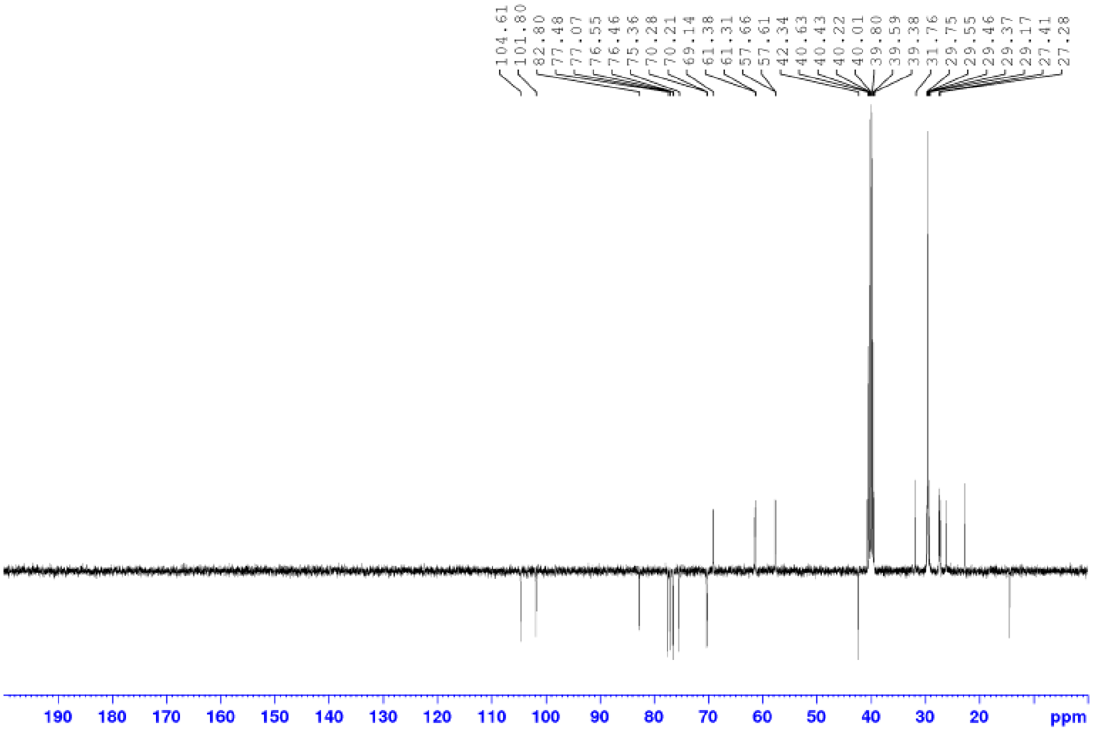

Figure 27. ^{13}**C-NMR 17a**

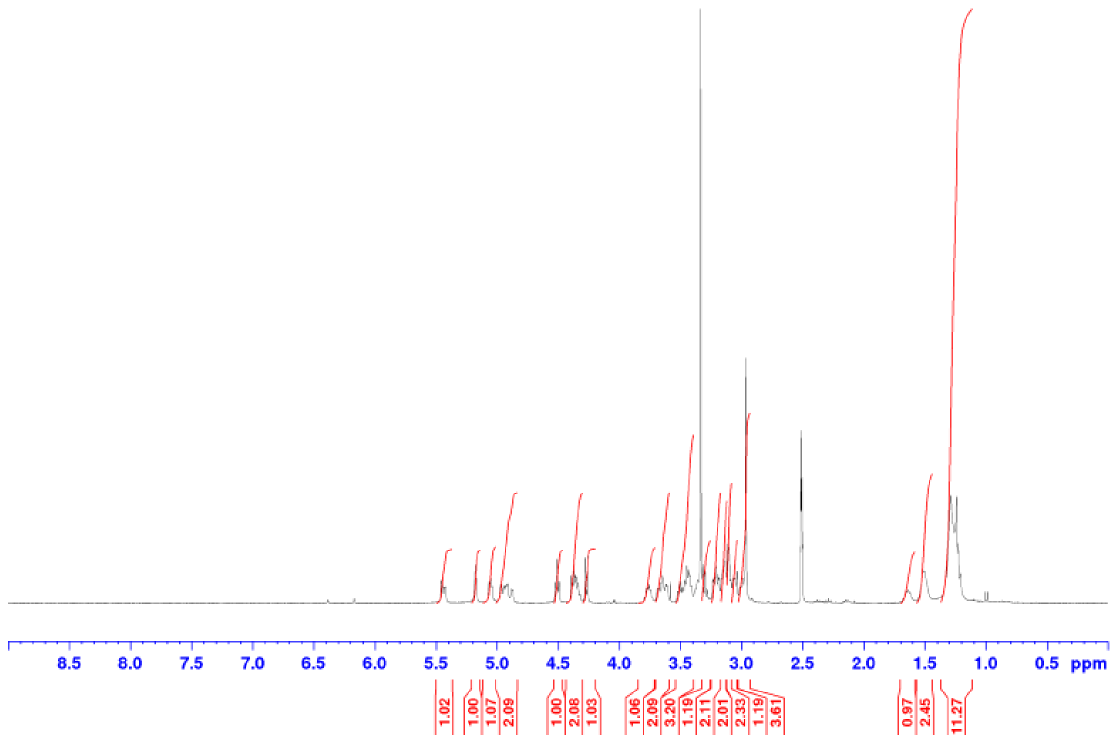

Figure 28. ^{1}**H-NMR 18a**

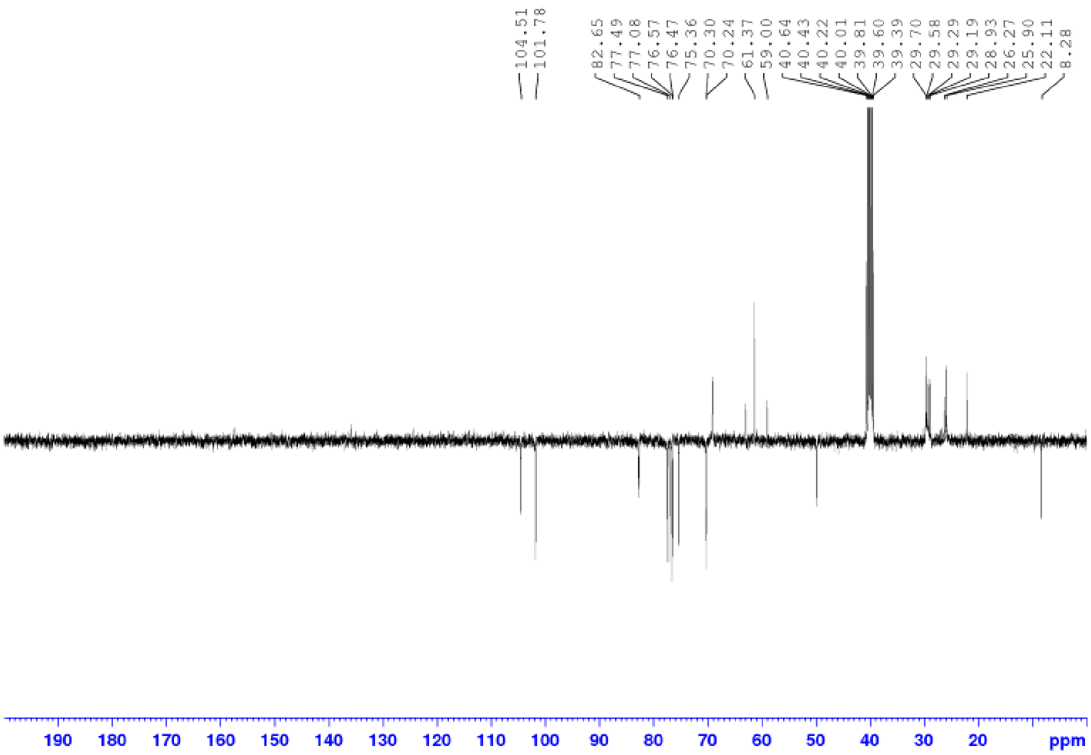

Figure 29. ^{13}**C-NMR 18a**

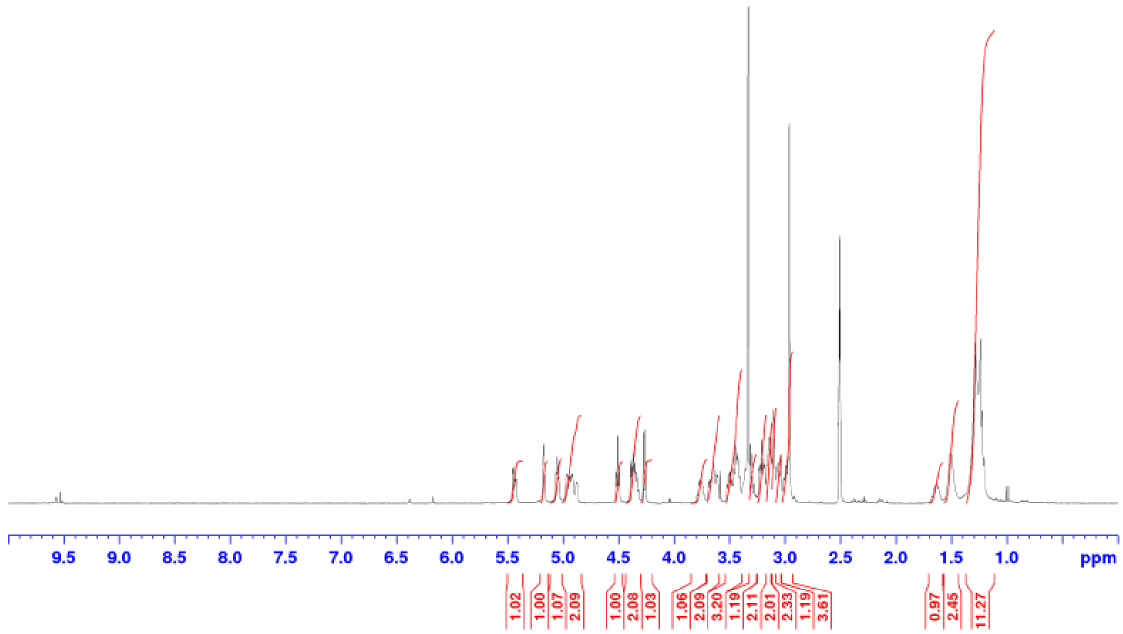

Figure 30. ^{1}H-NMR 19a

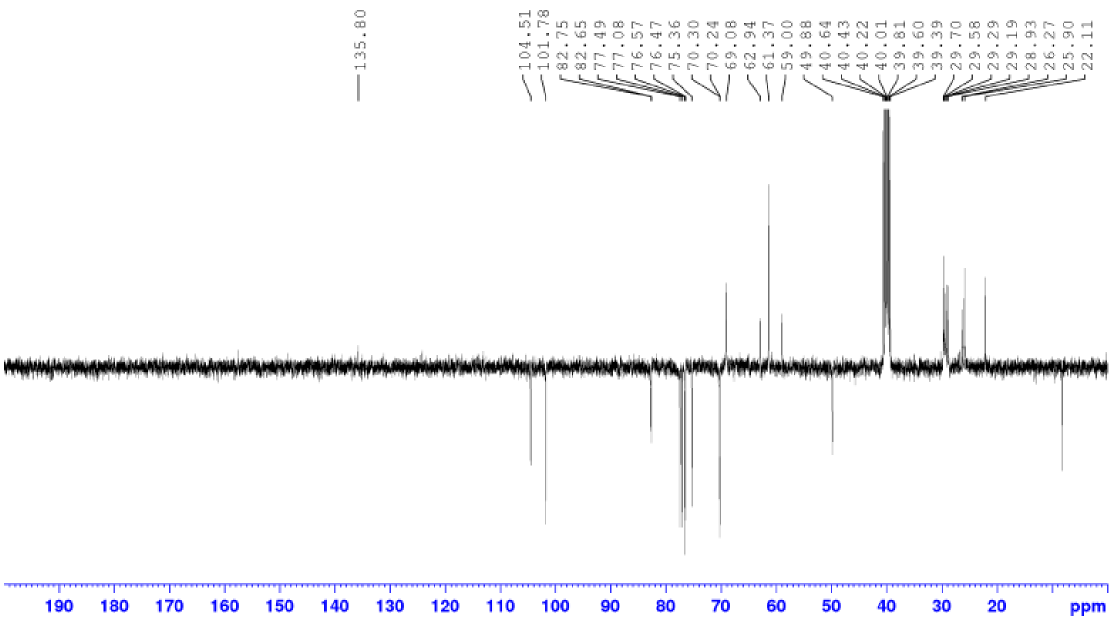

Figure 31. ^{13}**C-NMR 19a**

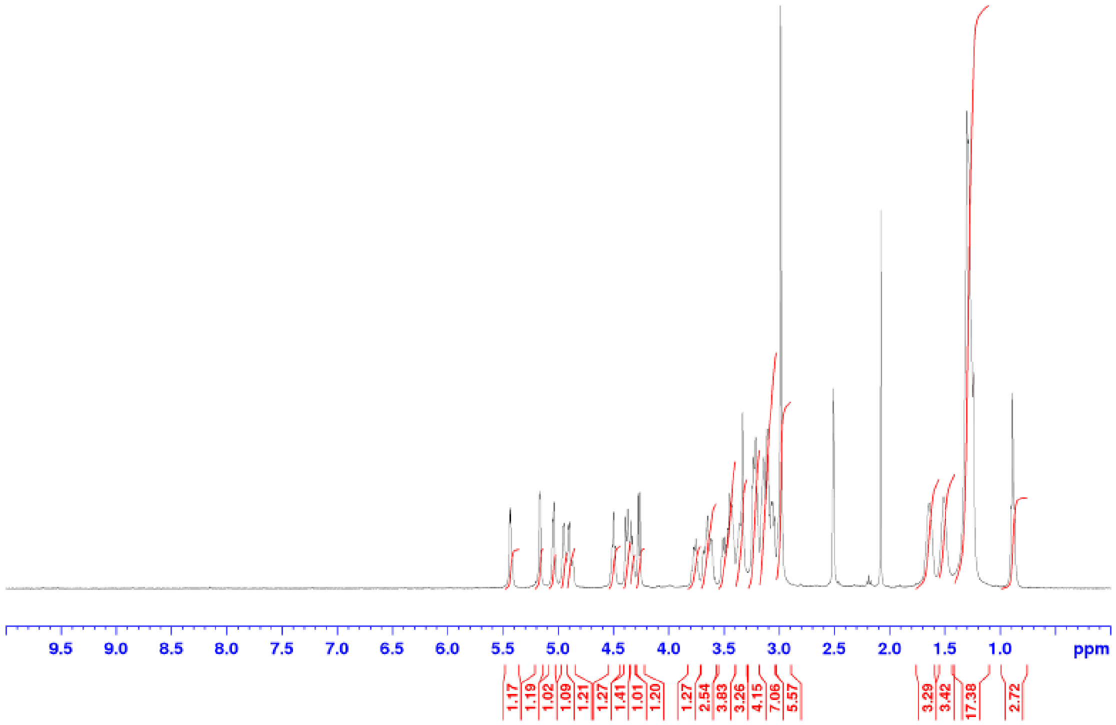

Figure 32. ^{1}H-NMR **20a**

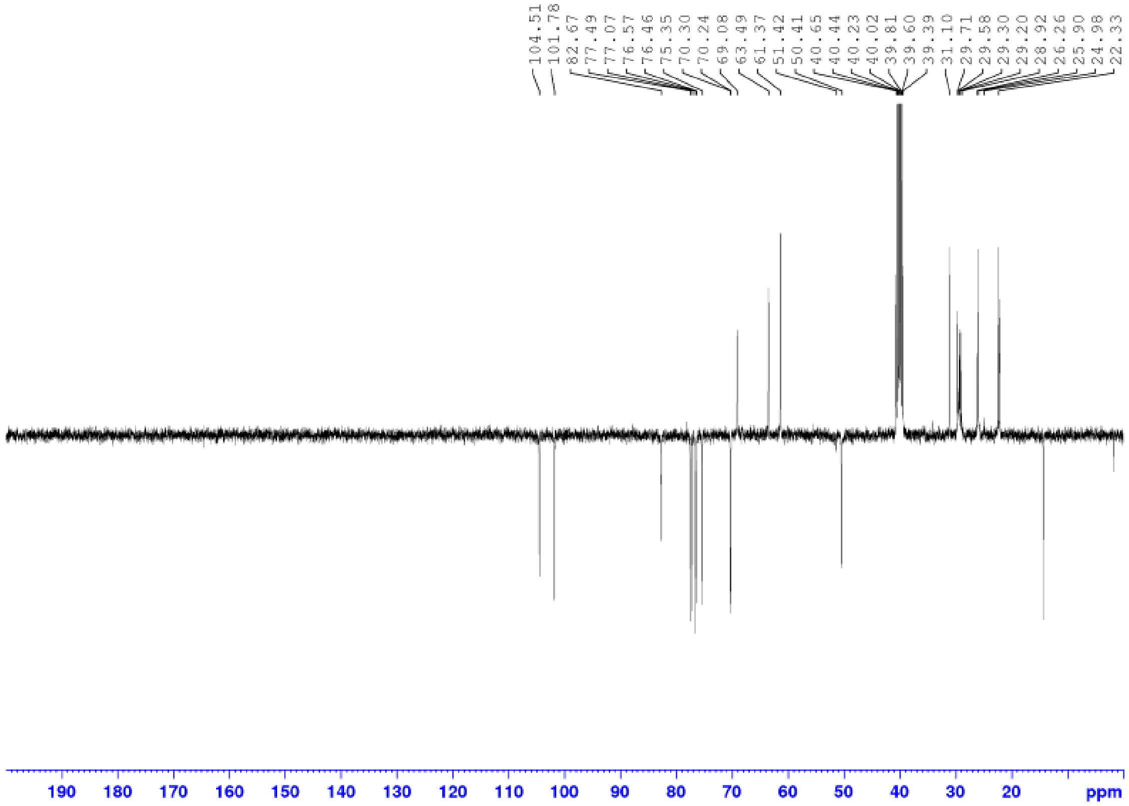

Figure 33. ^{13}**C-NMR 20a**

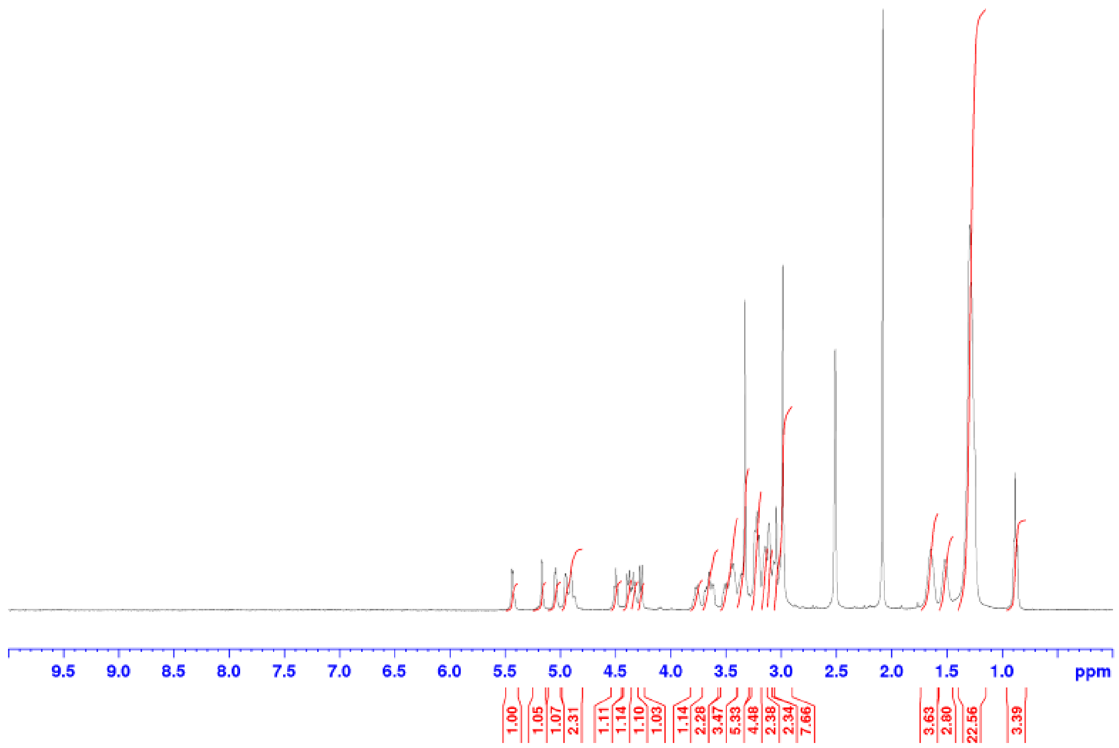

Figure 34. ^{1}**H-NMR 21a**

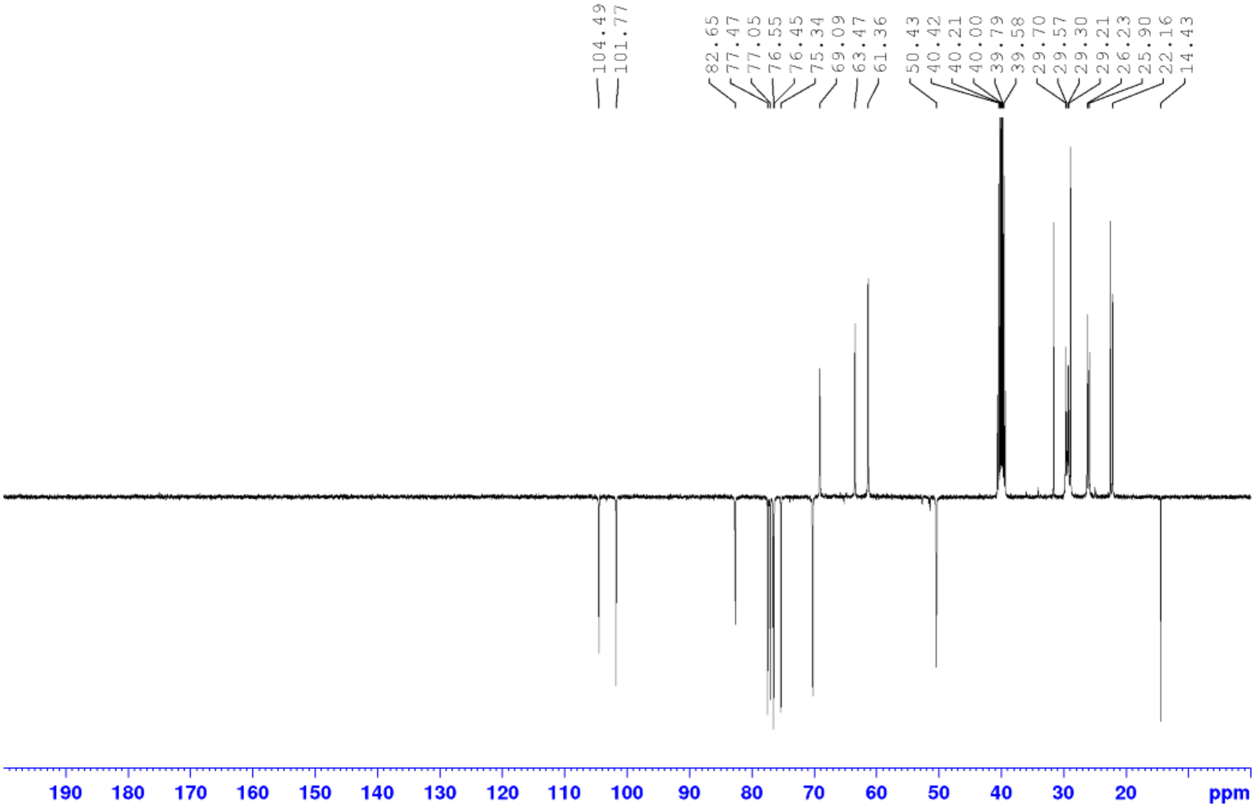

Figure 35. ^{13}C-NMR 21a

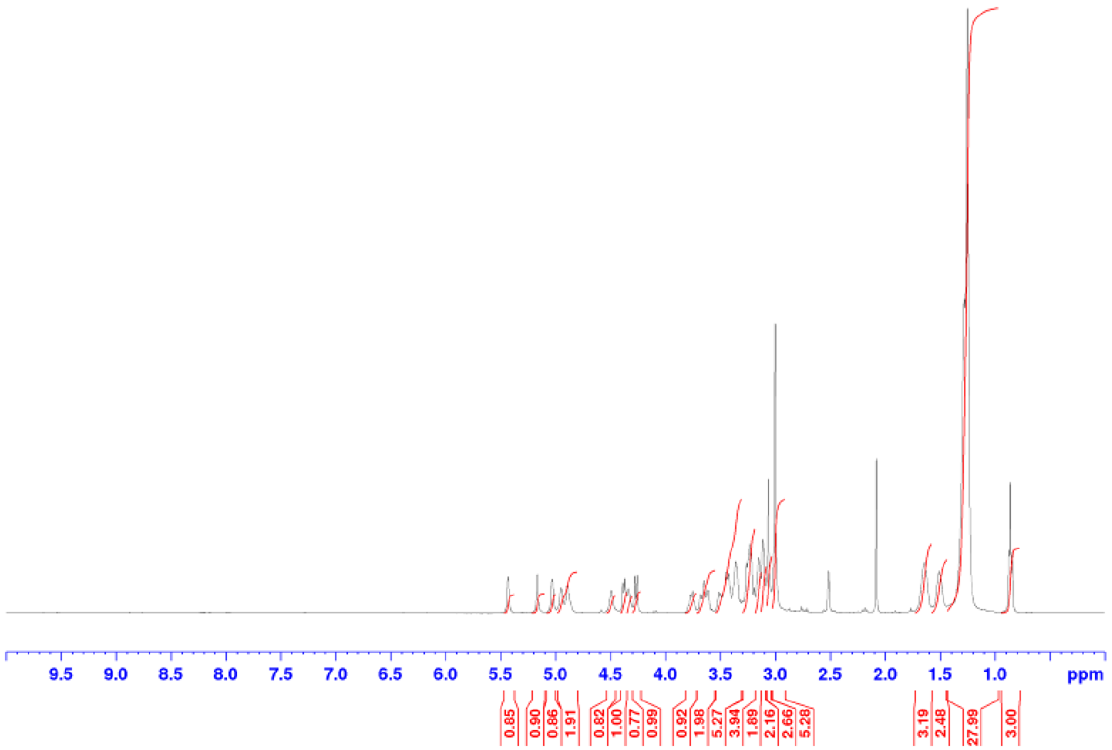

Figure 36. ^{1}**H-NMR 22a**

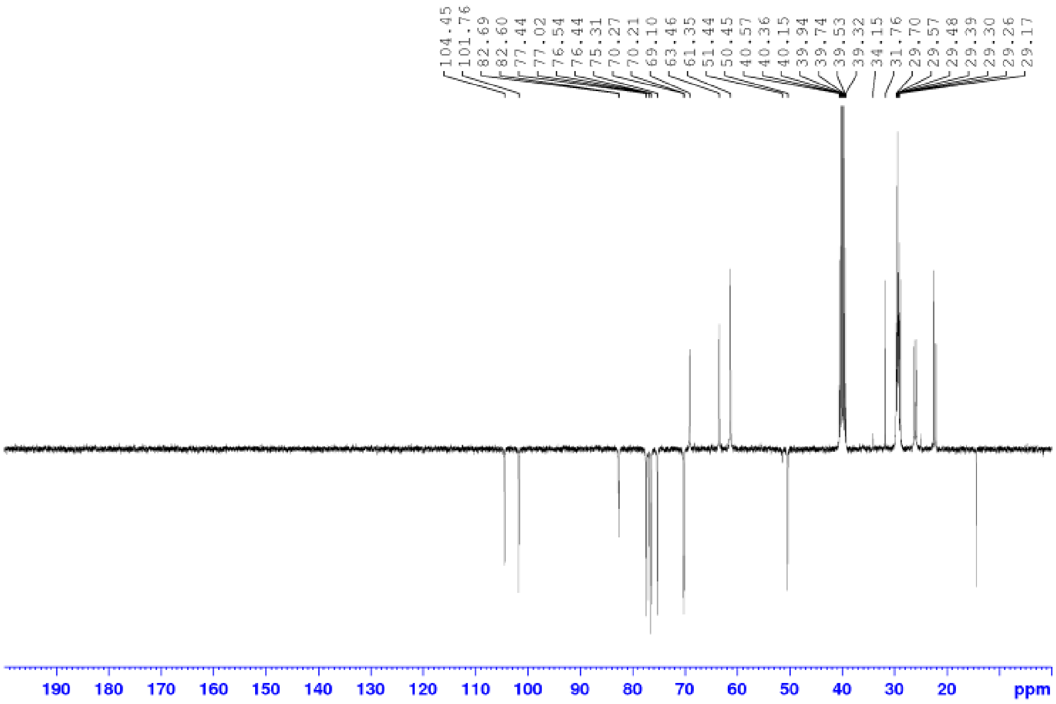

Figure 37. ^{13}**C-NMR 22a**

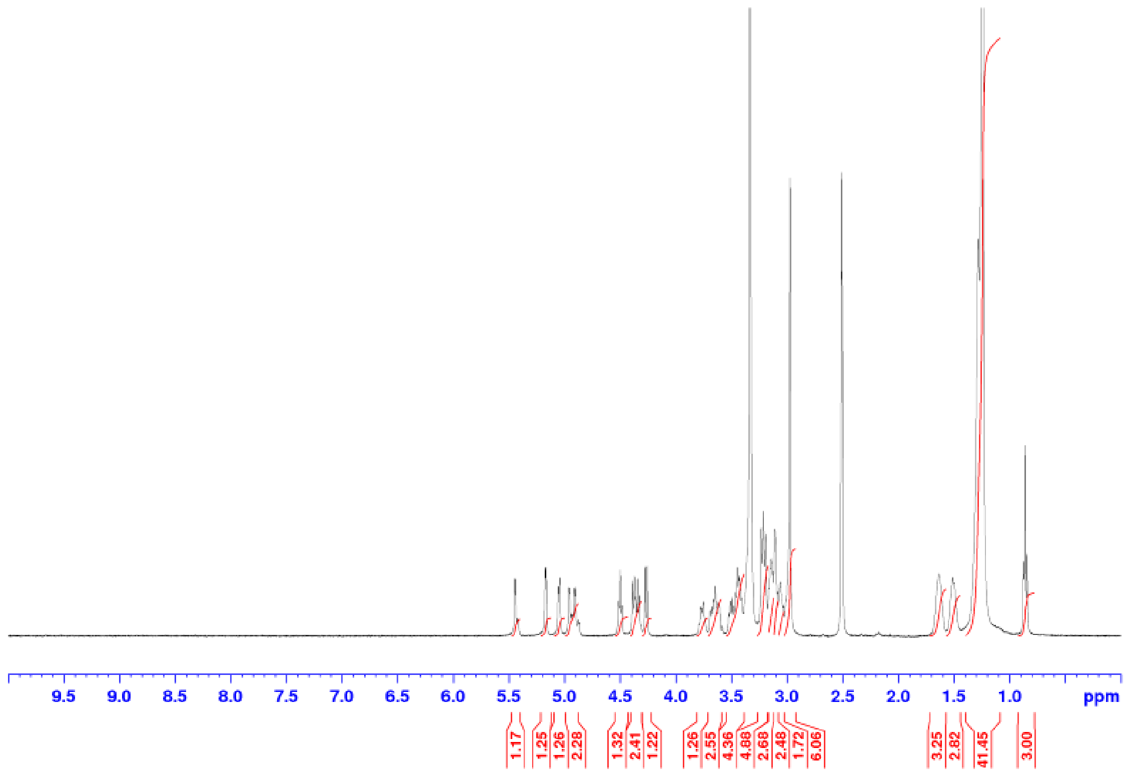

Figure 38. ^{1}**H-NMR 23a**

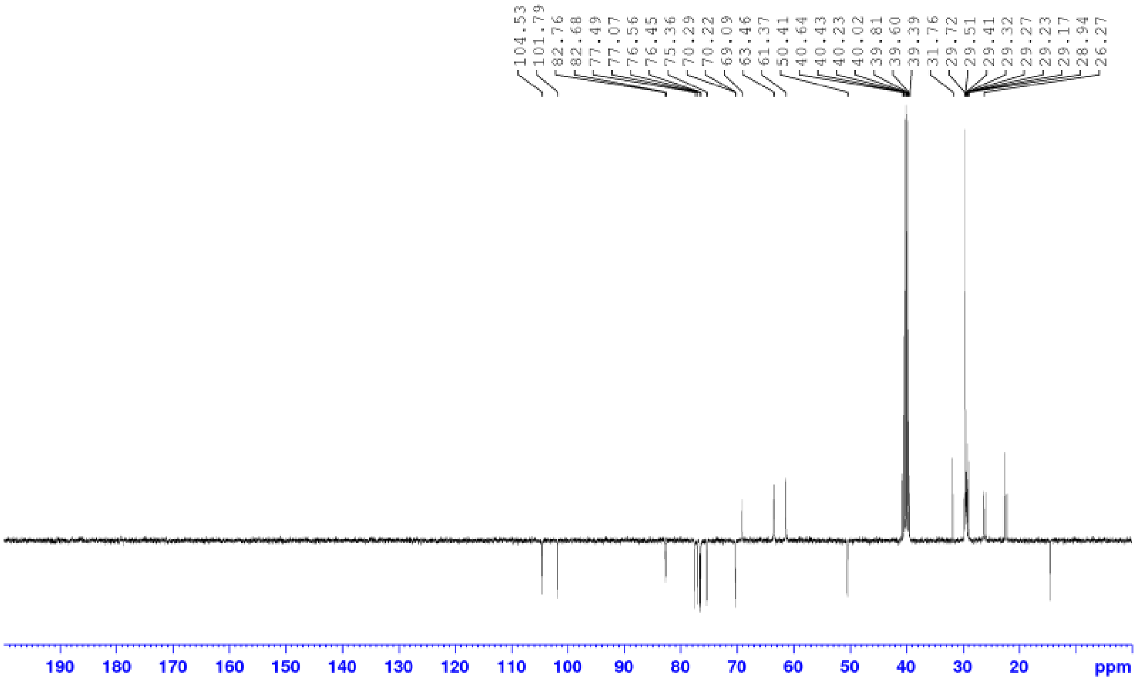

Figure 39. ^{13}C-NMR 23a
